# ACCORD: A Multicentre, Seamless, Phase 2 Adaptive Randomisation Platform Study to Assess the Efficacy and Safety of Multiple Candidate Agents for the Treatment of COVID-19 in Hospitalised Patients: A structured summary of a study protocol for a randomised controlled trial

**DOI:** 10.1186/s13063-020-04584-9

**Published:** 2020-07-31

**Authors:** Tom Wilkinson, Rupert Dixon, Clive Page, Miles Carroll, Gareth Griffiths, Ling-Pei Ho, Anthony De Soyza, Timothy Felton, Keir E. Lewis, Karen Phekoo, James D. Chalmers, Anthony Gordon, Lorcan McGarvey, Jillian Doherty, Robert C. Read, Manu Shankar-Hari, Nuria Martinez-Alier, Michael O’Kelly, Graeme Duncan, Roelize Walles, James Sykes, Charlotte Summers, Dave Singh, on behalf of the ACCORD Collaborators

**Affiliations:** 1 University of Southampton, Southampton, Hampshire UK; 2 NIHR Southampton Biomedical Research Centre, Southampton, UK; 3 IQVIA, Reading, UK; 4 King’s College London, London, UK; 5 Public Health England, London, UK; 6 NIHR Oxford Biomedical Research Centre, University of Oxford, Oxford, UK; 7 Population Health Sciences Institute, Faculty of Medical Sciences, Newcastle University, Newcastle upon Tyne, UK; 8 Manchester NIHR Biomedical Research Centre, University of Manchester, Manchester, UK; 9 Swansea University, Swansea, UK; 10 University Hospital Southampton NHS Foundation Trust, Southampton, UK; 11 University of Dundee, Dundee, UK; 12 Imperial College London, London, UK; 13 Queen’s University Belfast, Belfast, UK; 14 Evelina London Children’s Hospital Guy’s and St Thomas’ Hospital NHS Foundation Trust, London, UK; 15 IQVIA, Dublin, Ireland; 16 IQVIA, Edinburgh, UK; 17 University of Cambridge, Cambridge, UK; 18 University of Manchester, Manchester, UK

**Keywords:** COVID-19, randomised, platform study, master protocol, phase II

## Abstract

**Objectives:**

**Stage 1:** To evaluate the safety and efficacy of candidate agents as add-on therapies to standard of care (SoC) in patients hospitalised with COVID-19 in a screening stage.

**Stage 2:** To confirm the efficacy of candidate agents selected on the basis of evidence from Stage 1 in patients hospitalised with COVID-19 in an expansion stage.

**Trial design:**

ACCORD is a seamless, Phase 2, adaptive, randomised controlled platform study, designed to rapidly test candidate agents in the treatment of COVID-19. Designed as a master protocol with each candidate agent being included via its own sub-protocol, initially randomising equally between each candidate and a single contemporaneous SoC arm (which can adapt into 2:1). Candidate agents currently include bemcentinib, MEDI3506, acalabrutinib, zilucoplan and nebulised heparin. For each candidate a total of 60 patients will be recruited in Stage 1. If Stage 1 provides evidence of efficacy and acceptable safety the candidate will enter Stage 2 where a total of approximately 126 patients will be recruited into each study arm sub-protocol. Enrollees and outcomes will not be shared across the Stages; the endpoint, analysis and sample size for Stage 2 may be adjusted based on evidence from Stage 1. Additional arms may be added as new potential candidate agents are identified via candidate agent specific sub-protocols.

**Participants:**

The study will include hospitalised adult patients (≥18 years) with confirmed SARS-CoV-2 infection, the virus that causes COVID-19, that clinically meet Grades 3 (hospitalised – mild disease, no oxygen therapy), Grades 4 (hospitalised, oxygen by mask or nasal prongs) and 5 (hospitalised, non-invasive ventilation or high flow oxygen) of the WHO Working Group on the Clinical Characteristics of COVID-19 9-point category ordinal scale.

Participants will be recruited from England, Northern Ireland, Wales and Scotland.

**Intervention and comparator:**

Comparator is current standard of care (SoC) for the treatment of COVID-19. Current candidate experimental arms include bemcentinib, MEDI3506, acalabrutinib, zilucoplan and nebulised heparin with others to be added over time. Bemcentinib could potentially reduce viral infection and blocks SARS-CoV-2 spike protein; MEDI3506 is a clinic-ready anti-IL-33 monoclonal antibody with the potential to treat respiratory failure caused by COVID; acalabrutinib is a BTK inhibitor which is anti-viral and anti-inflammatory; zilucoplan is a complement C5 inhibitor which may block the severe inflammatory response in COVID-19 and; nebulised heparin has been shown to bind with the spike protein. ACCORD is linked with the UK national COVID therapeutics task force to help prioritise candidate agents.

**Main outcomes:**

Time to sustained clinical improvement of at least 2 points (from randomisation) on the WHO 9-point category ordinal scale, live discharge from the hospital, or considered fit for discharge (a score of 0, 1, or 2 on the ordinal scale), whichever comes first, by Day 29 (this will also define the “responder” for the response rate analyses).

**Randomisation:**

An electronic randomization will be performed by Cenduit using Interactive Response Technology (IRT). Randomisation will be stratified by baseline severity grade. Randomisation will proceed with an equal allocation to each arm and a contemporaneous SoC arm (e.g. 1:1 if control and 1 experimental arm; 1:1:1 if two experimental candidate arms etc) but will be reviewed as the trial progresses and may be changed to 2:1 in favour of the candidate agents.

**Blinding (masking):**

The trial is open label and no blinding is currently planned in the study.

**Numbers to be randomised (sample size):**

This will be in the order of 60 patients per candidate agent for Stage 1, and 126 patients for Stage 2. However, sample size re-estimation may be considered after Stage 1. It is estimated that up to 1800 patients will participate in the overall study.

**Trial Status:**

Master protocol version ACCORD-2-001 - Master Protocol (Amendment 1) 22^nd^ April 2020, the trial has full regulatory approval and recruitment is ongoing in the bemcentinib (first patient recruited 6/5/2020), MEDI3506 (first patient recruited 19/5/2020), acalabrutinib (first patient recruited 20/5/2020) and zilucoplan (first patient recruited 19/5/2020) candidates (and SoC). The recruitment dates of each arm will vary between candidate agents as they are added or dropped from the trial, but will have recruited and reported within a year.

**Trial registration:**

EudraCT 2020-001736-95, registered 28^th^ April 2020.

**Full protocol:**

The full protocol (Master Protocol with each of the candidate sub-protocols) is attached as an additional file, accessible from the Trials website (Additional file 1). In the interest in expediting dissemination of this material, the familiar formatting has been eliminated; this Letter serves as a summary of the key elements of the full protocol.

## Supplementary information

**Additional file 1.**

## Data Availability

When the database from each Candidate sub-protocol is locked, analysed and published we will make the data available to the academic community via www.clinicalstudydatarequest.com.

