# Supplementary material for: ACCORD: A Multicentre, Seamless, Phase 2 Adaptive Randomisation Platform Study to Assess the Efficacy and Safety of Multiple Candidate Agents for the Treatment of COVID-19 in Hospitalised Patients: A structured summary of a study protocol for a randomised controlled trial
Source: Trials. 2020 Jul 31;21:691. doi: 10.1186/s13063-020-04584-9 (PMC7393340; doi:10.1186/s13063-020-04584-9)
Supplement: Supplementary file 1 — Additional file 1. [file 13063_2020_4584_MOESM1_ESM.zip › ACCORD-2 MEDI3506_SubprotocolR0.pdf]

TITLE PAGE

Master Protocol Title: ACCORD-2: A Multicentre, Seamless, Phase 2 Adaptive Randomisation Platform Study to Assess the Efficacy and Safety of Multiple Candidate Agents for the Treatment of COVID-19 in Hospitalised Patients

Sub-protocol Number: ACCORD-2-003

Sub-protocol for Candidate Agent MEDI3506

Agent Owner Identifier: D9184C00001

Study Phase: 2

Sponsor Name: University Hospital Southampton NHS Foundation Trust

Legal Registered Address: **Southampton General Hospital**
Level E, Laboratory & Pathology Block, SCBR - MP138
Tremona Road
Southampton SO16 6YD, UK

Regulatory Agency Identifying Number(s): EudraCT 2020-001736-95

IRAS Number: **282769**

RHM Number: **MED1711**

Date of Sub-protocol: 29 April 2020

Version: Protocol Amendment 01, Final

Chief Investigator Signatory:

I have read this sub-protocol in its entirety and agree to conduct the study accordingly:

	**29/04/2020**
**Professor Tom Wilkinson MA Cantab MBBS PhD FRCP**	**Date**
**Professor of Respiratory Medicine and Honorary NHS Consultant Physician**	

TABLE OF CONTENTS

TABLE OF TABLES **5**

PROTOCOL AMENDMENT HISTORY **6**

1.0 SUB-PROTOCOL SUMMARY **7**
1.1 Overview of Sub-protocol **7**
1.2 Schedule of Activities **8**

2.0 BACKGROUND/RATIONALE IN SUPPORT OF MEDI3506 IN COVID-19 .... **12**
2.1 Dose Justification for MEDI3506 .... **13**

3.0 STUDY POPULATION **15**
3.1 Enrolment and Screening **15**
3.2 Eligibility Criteria **15**
3.2.1 Inclusion Criteria 15
3.2.2 Exclusion Criteria 15

4.0 STUDY ASSESSMENTS AND PROCEDURES **16**
4.1 Safety Assessments **16**
4.1.1 Adverse Events 16
4.1.2 Immunogenicity Assessments 17
4.2 Pharmacokinetic Assessments **17**

5.0 STUDY TREATMENT **18**
5.1 Treatment Plan **18**
5.2 Study Drug Administration **18**
5.3 Dose Modifications and Toxicity Management **18**
5.4 Concomitant Medications **18**
5.5 Study Drug Information **18**
5.5.1 Preparation/Handling/Storage/Accountability of Interventions 19
5.5.2 Investigational Product Inspection 19
5.5.3 Dose Preparation Steps 19
5.5.4 Treatment Administration 20
5.5.5 Monitoring of Dose Administration 20

6.0 REFERENCES **21**

7.0 APPENDICES **22**
Appendix 1 Abbreviations **23**

Appendix 2 Contraceptive Guidance 24
Appendix 3 Signature of Investigator 26

TABLE OF TABLES

Table 1 Investigational Product 18

PROTOCOL AMENDMENT HISTORY

Protocol Amendment 01 (dated 29 April 2020) replaces the original sub-protocol for MEDI3506 (dated 24 April 2020).

The amendment incorporates the following main changes:

- Clarification around second dose of MEDI3506 that can be administered on Day 15.
- Inclusion criterion regarding contraception updated (and corresponding appendix added).
- Exclusion criterion regarding history of heart failure added.
- Additional text regarding management of adverse events.
- Severe uncontrolled gastrointestinal adverse events added to the list of adverse events of special interest.

1.0 SUB-PROTOCOL SUMMARY

1.1 Overview of Sub-protocol

In addition to the Master Protocol, this sub-protocol contains information specific to the candidate agent MEDI3506, including a MEDI3506-specific Schedule of Activities (SoA) (Section 1.2). The background of the candidate agent and dose justification are explained (Section 2.0). One additional inclusion criterion and one additional exclusion criterion are added (Section 3.2.1 and 3.2.2, respectively). Safety assessments, potential risks, and adverse events of special interest (AESIs) for MEDI3506 are detailed (Section 4.1), and pharmacokinetic (PK) sample assessments have been added to the SoA (Section 1.2) and Section 4.2. The formulation and dose administration for MEDI3506 are also explained (Section 5.0). Additional details in regard to concomitant medications (Section 5.4) are included.

1.2 Schedule of Activities

	Screening	Baseline					
**Day (± Window)**	**Day -1, or Day 1**	**Day 1**	**Daily Until Hospital Discharge**	**Day 15[a] (±2 days)**	**Day 29[a] (±3 days)**	**Day 60 (±4 days) (Follow-up)**	**Day 90 (±6 days) (End of Study)**
ELIGIBILITY							
Informed consent	X						
Demographics	X						
Relevant medical history[b]	X						
Review of SARS-CoV-2 diagnostic tests	X						
Inclusion and exclusion criteria	X						
12-lead Electrocardiogram	X						
STUDY INTERVENTION							
Randomisation		X					
Administration of MEDI3506		X		X[c]			
Treatment with SoC		X	X				
STUDY PROCEDURES							
Clinical frailty score	X						
Diagnostic imaging (X-ray and/or computed tomography)	X						
Physical examination (including presenting symptoms, height, weight)	X						
Targeted physical examination (focused on lung auscultation)			X				
Vital signs, including temperature, pulse rate, blood pressure, respiratory rate, SpO_2		X[d]	X	X	X		

	Screening	Baseline					
**Day (± Window)**	**Day -1, or Day 1**	**Day 1**	**Daily Until Hospital Discharge**	**Day 15[a] (±2 days)**	**Day 29[a] (±3 days)**	**Day 60 (±4 days) (Follow-up)**	**Day 90 (±6 days) (End of Study)**
Clinical assessments[e]		X[d]	X	X	X		
Targeted medication review (including use of vasopressors)		X[d]	X	X	X		
Adverse event evaluation		X	X	X	X	X	X
Disease-related co-infection evaluation (including microbiologic/infectious agent assessment/results; bacteria, viral, fungi)		X	X				
Survival status		X	X	X	X	X	X
Blood gases and FiO_2 at worse PO_2[f]	X	X	X	X			
SAFETY LABORATORY							
Haematology, chemistry, liver function tests, coagulation[g]	X[h]	X[d,i]	Days 3, 5, 8, 11 (all ±1 day) while hospitalised				
NT-proBNP	X[h]	X[d,i]	Days 3, 5, 8, 11 (all ±1 day) while hospitalised				
Pregnancy test for females of childbearing potential	X[h]						X
RESEARCH LABORATORY							
Blood (SST) for exploratory inflammatory cytokine analysis		X	Day8	X	X		
Blood (sodium heparin tube) for PBMC phenotyping[j]		X	Day 8	X	X		

	**Screening**	**Baseline**					
**Day (± Window)**	**Day -1, or Day 1**	**Day 1**	**Daily Until Hospital Discharge**	**Day 15[a] (±2 days)**	**Day 29[a] (±3 days)**	**Day 60 (±4 days) (Follow-up)**	**Day 90 (±6 days) (End of Study)**
Blood (EDTA) for SARS-CoV-2 PCR (qualitative and quantitative)		X	Days 3, 5, 8, 11 (all ±1 day) while hospitalised	X	X		
Oropharyngeal/nasal swab for SARS-CoV-2 PCR (qualitative and quantitative)		X	Days 3, 5, 8, 11 (all ±1 day) while hospitalised	X	X		
Saliva for SARS-CoV-2 PCR (qualitative and quantitative)		X	Days 3, 5, 8, 11 (all ±1 day) while hospitalised	X	X		
Blood (SST) for SARS-CoV-2 serology research (host response)		X	Day 8	X	X	X	
Blood (PAXGENE) for transcriptome analysis (host genome)[k]		X	Day 8	X			
Blood (EDTA) host genome (host DNA)[k]		X					
Mid-turbinate nasal swab viral genome[k]		X					
Blood sample for MEDI3506 PK		X[l]	Days 3, 5, 8, 11 (all ±1 day) while hospitalised	X[m]	X[n]	X[n]	X[n]
Blood sample for MEDI3506 ADA		X[o]			X or discharge if before Day 29		X

ADA=antidrug antibody; EDTA=ethylenediaminetetraacetic acid; FiO_2=fractional inspired oxygen; IV=intravenous; NT-proBNP=N-terminal pro hormone B-type natriuretic peptide; PBMC=peripheral blood mononuclear cell; PCR=polymerase chain reaction; PK=pharmacokinetic(s); PO_2= partial pressure of oxygen; RT PCR=reverse transcription polymerase chain reaction; SARS-CoV-2=Severe acute respiratory syndrome coronavirus 2; SOC=standard of care; SpO_2=oxygen saturation; SST=serum separator tube.

[a] These visits will be performed even if a patient has already been discharged. If discharged prior to scheduled visit, in-person visits are preferred, but recognising that quarantine and other factors may limit the patient's ability to return to the clinic, these visits may be conducted by telephone or with a home visit by study staff. For visits conducted by telephone, it will not be possible to perform some scheduled assessments (eg, vital signs). The Day 29 assessments will also be performed, where possible, for patients who discontinue the study prematurely.

[b] Medical history includes estimated date and time of first symptoms and number of co-morbidities (eg, respiratory, cardiovascular, metabolic, malignancy, endocrine, gastrointestinal, immunologic, renal).

[c] A second dose of 300 mg IV MEDI3506 will be administered if the patient becomes invasively ventilated on, or before, Day 15 but after randomisation and remains invasively ventilated on Day 15.

[d] Baseline assessments should be performed prior to study drug administration.

[e] Includes ordinal score, National Early Warning Score 2 (NEWS2), oxygen requirement, noninvasive or invasive ventilator requirement, including start and stop of low- or high-flow oxygen supply or of any form of ventilation etc.

[f] Capillary if arterial line in situ or arterial blood gases are not measured that day as part of usual clinical care.

[g] For parameters, see Table 5 of the master protocol.

[h] Laboratory tests performed in the 48 hours prior to enrolment will be accepted for determination of eligibility.

[i] Any laboratory tests performed as part of routine clinical care within the specified visit window can be used for safety laboratory testing.

[j] Samples collected for immediate laboratory processing and frozen storage.

[k] Samples collected dependent on capacity of study centre, need for reduced study burden on staff, and potentially limited access to patients.

[l] Blood sample for PK on Day 1 collected pre-administration and 15 minutes post-administration of study intervention.

[m] If patient receives the second dose on Day 15, blood sample for PK on Day 15 collected pre-dose and 15 minutes post-administration of study intervention.

[n] Only if second dose given on Day 15.

[o] Sample collected pre- administration of study intervention.

2.0 BACKGROUND/RATIONALE IN SUPPORT OF MEDI3506 IN COVID-19

COVID-19 pneumonia is characterised by lung vascular endothelium and airway and alveolar epithelial damage, resulting in cytokine release, and widespread pulmonary and systemic inflammation. In severe cases, this results in alveolar oedema, hypoxaemia, acute respiratory distress syndrome, and death. COVID-19 is cytopathic, which results in release of Interleukin (IL)-33 from pulmonary epithelial and endothelial cells to drive and amplify the cycle of inflammation and cell damage.

Lung histology of patients with severe acute respiratory syndrome coronavirus (SARS-CoV) highlights secondary haemophagocytis lymphohistiocytosis (sHLH) as a feature of severe disease.[1] Many patients with severe COVID-19 disease have clinical features consistent with sHLH, which is recognised to induce and/or amplify pulmonary and systemic inflammation.[1]

IL-33 is an upstream alarmin cytokine that is rapidly released from damaged and dying lung epithelial and endothelial cells.[2,4] It is released by pulmonary epithelial cells infected with human rhinovirus, respiratory syncytial virus, and viral influenza.[2,5] Animal models of acute and chronic lung injury are similarly associated with elevated IL-33 and upregulation of type 1/2 cytokines (eg, IL-6) and preclinical studies show IL-33 blockade can attenuate inflammation and improve lung function and symptoms.[6,7,8] Preclinical data indicate that IL-33 is a crucial amplifier of inflammation mediated by sHLH.[9,10]

Two Phase 2 studies support a role for anti-IL-33 therapy in severe asthma, demonstrating that targeting IL-33 significantly improves lung function compared with placebo (NCT03387852) and reduces blood eosinophils (NCT03469934). MEDI3506 is a fully human immunoglobulin G1 monoclonal antibody (developed by AstraZeneca, the candidate agent owner) that binds to full length and mature forms of human IL-33 with exceptionally high affinity, thereby preventing IL-33 binding to soluble and membrane-bound forms of Suppression of Tumorigenicity 2 (ST2), also known as IL-1RL1 (refer to MEDI3506 Investigator Brochure, Doc ID-004230484).

IL-33 drives pulmonary inflammation through ST2 which is expressed by several inflammatory cell types including mast cells, type1 and 2 innate lung cells, macrophages and endothelial cells. The IL-33/ST2 signalling pathway leads to production of inflammatory cytokines such as IL-6 and granulocyte-macrophage colony-stimulating factor by these cell types. The IL-33/ST2 pathway is also implicated in the development of sHLH. Consequently, by blocking IL-33 mediated/ST2 signalling, MEDI3506 has potential to stop the cycle of pulmonary injury found in COVID-19 pneumonia and thereby prevent progression of acute respiratory failure.

2.1 Dose Justification for MEDI3506

One Phase 1 clinical study of MEDI3506 (Study D9180C00001) has been completed. Study D9180C00001 was a first-in-human, randomised, placebo-controlled, blinded (investigator and participant blinded; sponsor unblinded) clinical study in 88 participants (Part I: single ascending dose (SAD) in 56 healthy volunteers with a history of mild atopy; Part II: multiple ascending dose (MAD) in 24 participants with mild chronic obstructive pulmonary disease; Part III: single dose in 8 healthy Japanese volunteers) to evaluate the safety, tolerability, PK, and immunogenicity of MEDI3506. MEDI3506 was found to be generally safe and well tolerated, and there were no safety concerns following administration of 300 mg MEDI3506 intravenous (IV) to a total of 12 participants in Parts I and III of the study. In Part 2 of the study, COPD patients received 3 doses of MEDI3506 subcutaneously at 14-day intervals; overall adverse events appear similar between MEDI3506 and placebo groups. One subject experienced an adverse event leading to discontinuation of investigational product and 3 subjects experienced serious adverse events (of which 2 were fatal; non-small cell carcinoma of lung, and coronary artery thrombosis). All 4 of these events were considered not related to investigational product by the Investigator. Further details are given in the current version of the Investigator's Brochure.

In the present study, MEDI3506 will be administered to patients as a single 300 mg IV dose. A second dose of 300 mg IV MEDI3506 will be administered if the patient becomes invasively ventilated on, or before, Day 15 but after randomisation and remains invasively ventilated on Day 15. A dose of 300 mg MEDI3506 IV is predicted to have a safety margin of > 47-fold for both maximum observed concentration and area under the concentration-time curve (AUC) over exposures at the no observed adverse effect level (150 mg/kg) based on a 4-week Good Laboratory Practice toxicology study.

In the Phase 1 study, exposure to a single 300 mg IV dose of MEDI3506 in terms of maximum concentration at steady-state was 87.3 and 81 μg/mL, and area under the concentration-time curve from zero to infinity ($AUC_{0\text{-inf}}$) was 746 and 556 μg/mL*day for healthy participants with a history of mild atopy (ie, SAD 7 cohort) and healthy Japanese participants (ie, Japanese single dose [J-SD] cohort), respectively. The clearance for the 300 mg IV doses was 0.406 and 0.542 L/day for the SAD 7 and J-SD cohorts, respectively. The mean terminal half-life of MEDI3506 ranged from 11.7 to 17.3 days. Linear PK of MEDI3506 in that study was observed.

The optimal serum concentration for MEDI3506 efficacy in COVID-19 is not known. Based on a mouse lung damage model and Phase 1 clinical PK data, a MEDI3506 dose of 300 mg IV is predicted to suppress IL-33 levels > 99% at peak drug concentration in sputum based on PK/PD modelling. At this dose, the IL-33 suppression in sputum is predicted to be maintained at approximately 98%. If patients become invasively ventilated on, or before, Day 15 but after randomisation and remain invasively ventilated on Day 15 (ie, do not show an improvement in health status following the initial dose), a second dose of MEDI3506 will be administered on that

day, as this time point aligns with the approximate terminal half-life of MEDI3506. Although levels of IL-33 in the lungs of invasively ventilated patients are not known, it is hypothesised that there will be ongoing epithelial and endothelial cell damage in these patients. Therefore, a second dose of MEDI3506 may show an additional benefit.

3.0 STUDY POPULATION

3.1 Enrolment and Screening

Enrolment, screening and first dose of MEDI3506 must take place with 24 hours of admission to hospital.

3.2 Eligibility Criteria

Overall inclusion and exclusion criteria are presented in Sections 5.1 and 5.2 of the Master Protocol, respectively. The following sections detail variations to those criteria that are specific to this sub-protocol.

3.2.1 Inclusion Criteria

Inclusion criterion 3 from the Master Protocol, related to contraception, will be replaced with the following:

3. a) Male subjects:

- A male subject must agree to use contraception as detailed in Appendix 2 of this protocol during the treatment period and for at least 12 weeks after the last dose of study treatment and refrain from donating sperm during this period.

b) Female subjects:

- A female subject is eligible to participate if she is not pregnant (see Appendix 2), not breastfeeding, and at least 1 of the following conditions applies:

  i) Not a woman of childbearing potential (WOCBP) as defined in Appendix 2.
  OR
  ii) A WOCBP who agrees to follow the contraceptive guidance in Appendix 2 during the treatment period and for at least 12 weeks after the last dose of study treatment.

3.2.2 Exclusion Criteria

Additional exclusion criteria that are specific to the sub-protocol are as follows:

X1. Active tuberculosis defined as requiring current treatment for tuberculosis.
X2. A known family history of heart failure defined as either of the following:
  a. ≥ 2 first degree relatives with clinically significant heart failure, or
  b. ≥ 1 first degree relative with heart failure known to be heritable (eg, hypertrophic cardiomyopathy), unless inheritance was previously excluded by genetic testing.

4.0 STUDY ASSESSMENTS AND PROCEDURES

Safety data from this sub-protocol will be reviewed by the Independent Data and Safety Monitoring Committee (IDMC), for additional details refer to the Master Protocol, Section 9.7.2. The IDMC will review data on an ongoing basis to ensure patient safety (Refer to IDMC Charter).

In addition to the study assessments and procedures described in Section 8.0 of the Master Protocol, assessments specific to the sub-protocol will be performed as described in the following sections. No further efficacy assessments specific to the sub-protocol will be performed. The SoA for this sub-protocol is presented in Section 1.2.

4.1 Safety Assessments

Safety data exchange details will be described in a safety handling plan, a safety data exchange agreement or the equivalent to ensure timely transfer of data to AstraZeneca.

4.1.1 Adverse Events

Refer to Section 8.4 and Appendix 4 in the Master Protocol for information on definitions and procedures for recording, evaluating, follow-up, and reporting of AEs and SAEs.

All AEs should be managed according to physician judgement and applicable local/national guidelines. Section 7.1 of the Master Protocol describes the criteria (including AEs) where a patient should be withdrawn from study treatment, ie, where a second dose of MEDI3506 should not be administered when it otherwise would be according to Section 5.2.

4.1.1.1 Potential Risks

MEDI3506 is not registered as a marketed therapy in any indication. There are no identified risks associated with MEDI3506. Potential risks of clinical significance for this study based on mechanism of action are opportunistic infections. In addition, sST2 is a prognostic biomarker of cardiac overload and chronic heart failure. It is not clear if elevated sST2 is an epiphenomenon, or if sST2 contributes to pathology by acting as a decoy receptor and lowering concentrations of free IL-33 in tissues. However, the mechanism linking elevated sST2 to heart failure is unclear. For further details on potential risks, refer to the most current version of the Investigator’s Brochure.

4.1.1.2 Adverse Events of Special Interest

The following are defined as AESIs for MEDI3506 in this protocol:

- Serious hypersensitivity (including Type 1 to 4 hypersensitivity reactions), eg, anaphylaxis and other severe allergic reactions, as well as immune complex disease.
- Hepatic function abnormality meeting the definition of Hy’s law.

- Cardiac events (including angina, myocardial infarction, congestive heart failure, symptomatic atherosclerotic vascular disease, cor pulmonale, or arrhythmia).
- Serious infections (including opportunistic infections and viral reactivations), eg, herpes simplex virus/varicella zoster virus, Epstein Barr virus/cytomegalovirus, tuberculosis, and all other opportunistic infections listed in the Guidelines for the Prevention and Treatment of Opportunistic Infections in Adults and Adolescents with Human Immunodeficiency Virus.[11]
- New malignancy.
- Severe uncontrolled gastrointestinal adverse events

The AESIs defined above will be monitored by the IDMC (ACCORD protocol) carefully in order to characterize any association with the use of this investigational product.

4.1.2 Immunogenicity Assessments

Blood samples for determination of antidrug antibody in serum will be collected as specified in the SoA (Section 1.2). Samples will be collected, labelled, stored, and shipped as detailed in the laboratory manual.

4.2 Pharmacokinetic Assessments

Blood samples will be collected for measurement of serum concentrations of MEDI3506 as specified in the SoA (Section 1.2). A maximum of 5 samples are scheduled to be taken during the study. Samples will be collected, labelled, stored, and shipped as detailed in the laboratory manual.

5.0 STUDY TREATMENT

5.1 Treatment Plan

See SoA (Section 1.2 and Section 5.2).

5.2 Study Drug Administration

MEDI3506 will be administered to participants as a single 300 mg IV dose. A second dose of 300 mg IV MEDI3506 will be administered if the patient becomes invasively ventilated on, or before, Day 15 but after randomisation and remains invasively ventilated on Day 15.

5.3 Dose Modifications and Toxicity Management

Not applicable.

5.4 Concomitant Medications

Refer to Section 6.5 of the Master Protocol.

Live vaccines should not be used until 12 weeks after last dose of MEDI3506

5.5 Study Drug Information

Table 1 Investigational Product

**Intervention Name**	**MEDI3506**
Type	Biologic
Dose Formulation	Supplied in 2R vial with nominal 1 mL of MEDI3506 containing 20 mM L-histidine/L-histidine-hydrochloride, 220 mM L-arginine-hydrochloride, 0.03% (w/v) polysorbate 80, pH 5.5
Unit Dose Strength	150 mg/mL
Dosage Level	300 mg
Route of Administration	IV
Use	Experimental
IMP and NIMP	IMP
Sourcing	AstraZeneca
Packaging and Labelling	Single vial kits with a unique number that is printed on all labels within the kit (ie, the outer carton label and the label of the vial within the carton). Labels will be prepared in accordance with Good Manufacturing practice and local regulatory requirements.

IMP=investigational medicinal product; IV=intravenous; NIMP=non-investigational medicinal product.

5.5.1 Preparation/Handling/Storage/Accountability of Interventions

1. The investigator or designee must confirm appropriate temperature conditions have been maintained during transit for all study intervention received and any discrepancies are reported and resolved before use of the study intervention.
2. Only participants enrolled in the study may receive study intervention and only authorised site staff may supply or administer study intervention. All study intervention must be stored in a secure, environmentally-controlled, and monitored (manual or automated) area in accordance with the labelled storage conditions.

5.5.2 Investigational Product Inspection

Each vial allocated for dose preparation should be inspected. MEDI3506 is supplied as a clear to opalescent sterile liquid.

If there are any defects noted with the investigational product, the investigator and site monitor should be notified immediately. Refer to the Product Complaint section in the Investigational Medicinal Product manual for further instructions.

5.5.3 Dose Preparation Steps

MEDI3506 was found to be compatible with polypropylene plastic syringes and administration set with a 0.2 micron in-line filter for IV administration.

MEDI3506 does not contain preservatives and any unused portion must be discarded. Preparation of study intervention is to be performed aseptically.

Total (in use) storage time from needle puncture of the study intervention vial to start of administration should not exceed 4 hours at room temperature or 24 hours at 2°C to 8°C (36°F to 46°F). If storage time exceeds these limits, a new dose must be prepared from new vials.

A vial should be used only once to prepare a single dose.

The dose preparation steps are as follows:

1. Approximately 2 mL volume of MEDI3506 will be obtained by pooling the contents of 2 MEDI3506 vials (ie, 2 vials for 300 mg dose) into a 10-mL syringe (a new needle must be used for withdrawing study intervention from each vial). Add 8 mL of saline (final volume 10 mL).
2. Gently mix the MEDI3056 and saline in the syringe. The syringe should not be shaken or vigorously agitated.
3. Administer drug by IV injection over 1 to 2 minutes using an IV administration set with 0.2 micron in-line filter.
4. After administration of drug, flush IV line with 5 mL saline.

5.5.4 Treatment Administration

The first day of dosing is considered Day 1. Each patient must receive the entire volume of study intervention solution. Study intervention will be administered by IV push over a duration of no more than 3 minutes, inclusive of flush.

5.5.5 Monitoring of Dose Administration

If signs and symptoms indicative of an infusion reaction occur, the infusion will be stopped or slowed according to the judgment of the investigator.

As with any biologic product, allergic reactions to dose administration are possible. Therefore, appropriate drugs and medical equipment to treat acute anaphylactic reactions must be immediately available, and study personnel must be trained to recognise and treat anaphylaxis.

6.0 REFERENCES

1. Pei F, Zheng J, Gao ZF, et al. Lung pathology and pathogenesis of severe acute respiratory syndrome: a report of six full autopsies. Zhonghua Bing Li Xue Za Zhi 2005;34(10):656-60.
2. Mehta P, McAuley DF, Brown M et al. COVID-19: consider cytokine storm syndromes and immunosuppression. Lancet 2020;395(10229):1033-4.
3. Kearley J, Silver JS, Sanden C, et al. Cigarette smoke silences innate lymphoid cell function and facilitates an exacerbated type I interleukin-33-dependent response to infection. Immunity. 2015;42(3):566-79.
4. Martin NT, Martin MU. Interleukin 33 is a guardian of barriers and a local alarmin. Nat Immunol. 2016;17(2):122-31.
5. Jackson DJ, Makrinioti H, Rana BM, et al. IL-33-dependent type 2 inflammation during rhinovirus-induced asthma exacerbations in vivo. Am J Respir Crit Care Med. 2014;190(12):1373-82.
6. Allinne J, Scott G, Lim WK, et al. IL-33 blockade affects mediators of persistence and exacerbation in a model of chronic airway inflammation. J Allergy Clin Immunol. 2019;144(6):1624-37 e10.
7. Lin H, Fu J, Wang C, et al. Inflammation elevated IL-33 originating from the lung mediates inflammation in acute lung injury. Clinical Immunology 2016;173:32-43
8. Zhang Y, Lv R, Hu X, et al. The Role of IL-33 on LPS-Induced Acute Lung Injury in Mice. Inflammation 207;40:285–294.
9. Burn TN, Weaver L, Rood JE, et al, Genetic Deficiency of Interferon-γ Reveals Interferon-γ-Independent Manifestations of Murine Hemophagocytic Lymphohistiocytosis. Arthritis Rheumatol. 2020;72(2):335-47.
10. Rood JE, Rao S, Paessler M, et al. ST2 contributes to T-cell hyperactivation and fatal hemophagocytic lymphohistiocytosis in mice. Blood 2016;127(4):426-35.
11. NIH. Guidelines for the prevention and treatment of opportunistic infections in adults and adolescents with HIV. https://aidsinfo.nih.gov/contentfiles/lvguidelines/adult_oi.pdf. Published 2019. Accessed 10 April 2020.

7.0 APPENDICES

Appendix 1 Abbreviations

Abbreviation	Definition
AE	Adverse event
AESI	Adverse event of special interest
AUC	Area under the concentration time curve
CRO	Contract research organization
IDMC	Independent Data and Safety Monitoring Committee
IL	Interleukin
IV	Intravenous
J-SD	Japanese single dose
MAD	Multiple ascending dose
PD	Pharmacodynamic(s)
PK	Pharmacokinetic(s)
SAD	Single ascending dose
SARS-CoV	Severe acute respiratory syndrome coronavirus
sHLH	Secondary haemophagocytis lymphohistiocytosis
SoA	Schedule of Activities
ST2	Suppression of tumorigenicity 2
WOCBP	Woman of childbearing potential

Appendix 2 Contraceptive Guidance

Definitions:

Woman of Childbearing Potential (WOCBP)

A woman is considered fertile following menarche and until becoming postmenopausal unless permanently sterile (see below).

Women in the following categories are not considered WOCBP

1. Premenarchal
2. Premenopausal female with 1 of the following:
   a) Documented hysterectomy.
   b) Documented bilateral salpingectomy.
   c) Documented bilateral oophorectomy.
   Note: Documentation can come from the study center personnel's: review of the subject's medical records, medical examination, or medical history interview.
3. Postmenopausal female:
   a) A postmenopausal state is defined as no menses for 12 months without an alternative medical cause. A high follicle stimulating hormone (FSH) level in the postmenopausal range may be used to confirm a postmenopausal state in women not using hormonal contraception or hormonal replacement therapy (HRT). However, in the absence of 12 months of amenorrhea, a single FSH measurement is insufficient.
   b) Females on HRT and whose menopausal status is in doubt will be required to use 1 of the non-estrogen hormonal highly effective contraception methods if they wish to continue their HRT during the study. Otherwise, they must discontinue HRT to allow confirmation of postmenopausal status before study enrollment.

Contraception Guidance

Male subjects

- Male subjects with female partners of childbearing potential are eligible to participate if they agree to ONE of the following:
  - Are abstinent from penile-vaginal intercourse as their usual and preferred lifestyle (abstinent on a long-term and persistent basis) and agree to remain abstinent.
  - Agree to use a male condom plus partner use of a contraceptive method with a failure rate of <1% per year as described in the table below when having penile-vaginal intercourse with a woman of childbearing potential who is not currently pregnant.
- In addition, male subjects must refrain from donating sperm for the duration of the study and for 12 weeks after the last dose of study treatment.

- Male subjects with a pregnant or breastfeeding partner must agree to remain abstinent from penile-vaginal intercourse or use a male condom during each episode of penile penetration for the duration of the study and for 12 weeks after the last dose of study treatment.

Female subjects

Female subjects of childbearing potential are eligible to participate if they agree to use a highly effective method of contraception consistently and correctly as described in the table below.

Highly Effective Contraceptive Methods

**Highly Effective Contraceptive Methods That Are User Dependent** [a]<br>*Failure rate of <1% per year when used consistently and correctly.*
Combined (oestrogen and progestogen containing) hormonal contraception associated with inhibition of ovulation[b]<br>• Oral.<br>• Intravaginal.<br>• Transdermal.
Progestogen only hormonal contraception associated with inhibition of ovulation<br>• Oral.<br>• Injectable.
**Highly Effective Methods That Are User Independent** [a]
Implantable progestogen only hormonal contraception associated with inhibition of ovulation[b]<br>• Intrauterine device (IUD).<br>• Intrauterine hormone-releasing system (IUS).<br>Bilateral tubal occlusion.
**Vasectomized partner**<br>*A vasectomized partner is a highly effective birth control method provided that the partner is the sole male sexual partner of the WOCBP and the absence of sperm has been confirmed. If not, an additional highly effective method of contraception should be used.*
**Sexual abstinence**<br>*Sexual abstinence is considered a highly effective method only if defined as refraining from heterosexual intercourse during the entire period of risk associated with the study treatment. The reliability of sexual abstinence needs to be evaluated in relation to the duration of the study and the preferred and usual lifestyle of the subject.*
NOTES:<br>[a] Typical use failure rates may differ from those when used consistently and correctly. Use should be consistent with local regulations regarding the use of contraceptive methods for subjects participating in clinical studies.<br>[b] Hormonal contraception may be susceptible to interaction with the study treatment, which may reduce the efficacy of the contraceptive method. In this case, 2 highly effective methods of contraception should be utilized during the treatment period and for at least 12 weeks after the last dose of study treatment.

Appendix 3 Signature of Investigator

PROTOCOL TITLE: A Multicentre, Seamless, Phase 2 Adaptive Randomisation Platform Study to Assess the Efficacy and Safety of Multiple Candidate Agents for the Treatment of COVID-19 in Hospitalised Patients

SUB-PROTOCOL NO: ACCORD-2-003

SUB-PROTOCOL FOR CANDIDATE AGENT MEDI3506

VERSION: Amendment 01

This sub-protocol is a confidential communication of the Sponsor. I confirm that I have read this sub-protocol, I understand it, and I will work according to this sub-protocol, in conjunction with the Master Protocol for the overall platform study. I will also work consistently with the ethical principles that have their origin in the Declaration of Helsinki and that are consistent with Good Clinical Practices and the applicable laws and regulations. Acceptance of this document constitutes my agreement that no unpublished information contained herein will be published or disclosed without prior written approval from the Sponsor.

Instructions to the Investigator: Please SIGN and DATE this signature page. PRINT your name, title, and the name of the study centre in which the study will be conducted. Return the signed copy to the CRO/Sponsor.

I have read this sub-protocol in its entirety and agree to conduct this part of the study accordingly:

Signature of Investigator: ___________________________ Date: ________

Printed Name: ____________________________

Investigator Title: ____________________________

Name/Address of Centre: ____________________________

____

____
