# Supplementary material for: ACCORD: A Multicentre, Seamless, Phase 2 Adaptive Randomisation Platform Study to Assess the Efficacy and Safety of Multiple Candidate Agents for the Treatment of COVID-19 in Hospitalised Patients: A structured summary of a study protocol for a randomised controlled trial
Source: Trials. 2020 Jul 31;21:691. doi: 10.1186/s13063-020-04584-9 (PMC7393340; doi:10.1186/s13063-020-04584-9)
Supplement: Supplementary file 1 — Additional file 1. [file 13063_2020_4584_MOESM1_ESM.zip › ACCORD-2 zilucoplan_SubprotocolR0.pdf]

TITLE PAGE

Master Protocol Title: ACCORD-2: A Multicentre, Seamless, Phase 2 Adaptive Randomisation Platform Study to Assess the Efficacy and Safety of Multiple Candidate Agents for the Treatment of COVID-19 in Hospitalised Patients

Sub-protocol Number: ACCORD-2-006

Sub-protocol for Candidate Agent Zilucoplan

Study Phase: 2

Sponsor Name: University Hospital Southampton NHS Foundation Trust

Legal Registered Address: **Southampton General Hospital**
Level E, Laboratory & Pathology Block, SCBR - MP138
Tremona Road
Southampton SO16 6YD, UK

**Regulatory Agency Identifying Number(s):**	**EudraCT 2020-001736-95**
**IRAS Number:**	**282769**
**RHM Number:**	**MED1711**

Date of Sub-protocol: 05 May 2020

Version: Final

Sponsor Signatory:

I have read this sub-protocol in its entirety and agree to conduct this part of the study accordingly:

05 MAY 2020

Professor Anthony De Soyza BMSc, MBChB, PhD **Date**

Honorary Consultant Physician / Senior Lecturer in Respiratory Medicine

TABLE OF CONTENTS

TITLE PAGE **1**

TABLE OF CONTENTS **3**

TABLE OF FIGURES **5**

1.0 SUB-PROTOCOL SUMMARY **6**
- **1.1 Overview of Sub-protocol** **6**
- **1.2 Schedule of Activities** **7**

2.0 BACKGROUND/RATIONALE IN SUPPORT OF ZILUCOPLAN IN COVID-19 ..... **11**
- **2.1 Rationale for C5A involvement in COVID-19** **11**
  - 2.1.1 Background and Inflammation in COVID-19 infection 11
  - 2.1.2 Evidence that C5 Inhibition in COVID-19 Patients may prevent and possibly reverse Acute Respiratory Distress Syndrome (ARDS) 13
- **2.2 Dose selection for acute treatment of COVID-19** **15**

3.0 STUDY POPULATION **17**
- **3.1 Enrolment and Screening** **17**
- **3.2 Eligibility Criteria** **17**
  - 3.2.1 Inclusion Criteria 17
  - 3.2.2 Exclusion Criteria 18

4.0 STUDY ASSESSMENTS AND PROCEDURES **19**
- **4.1 Safety Assessments** **19**
  - 4.1.1 Adverse Events 19
  - 4.1.2 Clinical Safety Laboratory Assessments 20
- **4.2 Pharmacokinetic Assessments** **20**
- **4.3 Immunogenicity Assessments** **20**
- **4.4 Pharmacodynamic Assessments** **20**

5.0 STUDY TREATMENT **22**
- **5.1 Treatment Plan** **22**
- **5.2 Study Drug Administration** **22**
- **5.3 Dose Modifications and Toxicity Management** **22**
- **5.4 Concomitant Medications** **22**
  - 5.4.1 Rescue Medicine 22
- **5.5 Study Drug Information** **23**

6.0 REFERENCES....24

7.0 APPENDICES....26
Appendix 1 Abbreviations....27
Appendix 2 Contraceptive Guidance....28
Definitions:....28
Contraception Guidance....28
Appendix 3 Signature of Investigator....30

TABLE OF FIGURES

Figure 1 Escalating Phases of Disease Progression with COVID-19, with Associated Signs, Symptoms, and Potential Phase-Specific Therapies. 12
Figure 2 Mean Complement Activity as Measured by Sheep Red Blood Cell Assay (% Haemolysis)...16

1.0 SUB-PROTOCOL SUMMARY

1.1 Overview of Sub-protocol

ACCORD-2 is a seamless, Phase 2, adaptive, randomisation platform study, designed to rapidly test candidate agents in the treatment of coronavirus disease 2019 (COVID-19). The sub-study will treat hospitalised adult patients (≥18 years) who have infection with severe acute respiratory syndrome coronavirus 2 (SARS-CoV-2), the virus that causes COVID-19, as confirmed by laboratory tests and/or point of care tests with zilucoplan. Zilucoplan is a 15-amino acid macrocyclic peptide complement inhibitor designed for the treatment of conditions in which inappropriate activation of complement component 5 (C5) has been demonstrated to play a role. For inclusion, patients will need to have clinical status of Grade 3 (hospitalised – mild disease, no oxygen therapy) to Grade 5 (hospitalised – severe disease, noninvasive ventilation, or high-flow oxygen), as defined by a 9-point ordinal scale described in Section 8.1.1 of the Master Protocol.

Zilucoplan will be administered to patients infected with SARS-CoV-2 subcutaneously every day, for 14 days (if the patient is discharged before 14 days of treatment, zilucoplan should be stopped at time of discharge or 24 hours before discharge). This sub-protocol outlines the scientific rationale, additional eligibility criteria, treatment schema, and other specifics where different from the Master Protocol.

1.2 Schedule of Activities

	Screening	Baseline					
**Day (± Window)**	**Day -1 or Day 1**	**Day 1**	**Daily Until Hospital Discharge**	**Day 15[a] (±2 days)**	**Day 29[a] (±3 days)**	**Day 60[a] (±4 days) (Follow-up)**	**Day 90[a] (±6 days) (End of Study)**
ELIGIBILITY							
Informed consent	X						
Demographics	X						
Relevant medical history[b]	X						
Review of SARS-CoV-2 diagnostic tests	X						
Inclusion and exclusion criteria	X						
12-lead Electrocardiogram	X						
STUDY INTERVENTION							
Randomisation		X					
Administration of zilucoplan		X	X[c]				
Treatment with SoC		X	X				
Antibiotic prophylaxis		X	X[d]	X[d]	X[d]		
STUDY PROCEDURES							
Clinical frailty score	X						
Diagnostic imaging (X-ray and/or computed tomography)	X						
Physical examination (including presenting symptoms, height, weight)	X						
Targeted physical examination (focused on lung auscultation)			X				

	Screening	Baseline					
**Day (± Window)**	**Day -1 or Day 1**	**Day 1**	**Daily Until Hospital Discharge**	**Day 15[a] (±2 days)**	**Day 29[a] (±3 days)**	**Day 60[a] (±4 days) (Follow-up)**	**Day 90[a] (±6 days) (End of Study)**
Vital signs, including temperature, pulse rate, blood pressure, respiratory rate, SpO_2		X[e]	X	X	X		
Clinical assessments[f]		X[e]	X	X	X		
Targeted medication review (including use of vasopressors)		X[e]	X	X	X		
Adverse event evaluation		X	X	X	X	X	X
Disease related coinfection evaluation (including microbiologic/infectious agent assessment/results; bacteria, viral, fungi)		X	X				
Survival status		X	X	X	X	X	X
Blood gases and FiO_2 at worst PO_2[g]	X	X	X	X			
SAFETY LABORATORY							
Haematology, chemistry, liver function tests, coagulation[h]	X[i]	X[e,j]	Days 3, 5, 8, 11 (all ±1 day) while hospitalised				
Pregnancy test for females of childbearing potential	X[i]						
RESEARCH LABORATORY							
Blood (SST) for exploratory inflammatory cytokine analysis		X	Day 8	X	X		
Blood (sodium heparin tube) for PBMC phenotyping[k]		X	Day 8	X	X		
Blood (EDTA) for SARS-CoV-2 PCR (qualitative and quantitative)		X	Days 3, 5, 8, 11 (all ±1 day) while hospitalised	X	X		

	**Screening**	**Baseline**					
**Day (± Window)**	**Day -1 or Day 1**	**Day 1**	**Daily Until Hospital Discharge**	**Day 15[a] (±2 days)**	**Day 29[a] (±3 days)**	**Day 60[a] (±4 days) (Follow-up)**	**Day 90[a] (±6 days) (End of Study)**
Oropharyngeal/nasal swab for SARS-CoV-2 PCR (qualitative and quantitative)		X	Days 3, 5, 8, 11 (all ±1 day) while hospitalised	X	X		
Saliva for SARS-CoV-2 PCR (qualitative and quantitative)		X	Days 3, 5, 8, 11 (all ±1 day) while hospitalised	X	X		
Blood (SST) for SARS-CoV-2 serology research (host response)		X	Day 8	X	X	X	
Blood (PAXGENE) for transcriptome analysis (host genome)[l]		X	Day 8	X			
Blood (EDTA) host genome (host DNA)[l]		X					
Mid-turbinate nasal swab viral genome[l]		X					
Blood (EDTA and SST) for zilucoplan concentration analysis (PK) and exploratory biomarkers[m]		X	Days 3, 5, 8 (all ±1 day) while hospitalised[n]	X	X		
Blood (SST) ADA		X		X		X	

ADA=anti-drug antibody; C=component; EDTA=ethylenediaminetetraacetic acid; FiO_2=fraction of inspired oxygen; G-CSF=granulocyte colony-stimulating factor; GM-CSF= granulocyte-macrophage colony-stimulating factor; IFNγ=interferon gamma; IL=interleukin; IP-10=interferon gamma induced protein 10; MCP-1=monocyte chemoattractant protein 1; MASP-1=mannan-binding lectin serine protease; MIP=macrophage inflammatory protein; PBMC=peripheral blood mononuclear cell; PCR=polymerase chain reaction; PK=pharmacokinetics; PO_2=partial pressure of oxygen; SARS-CoV-2=severe acute respiratory syndrome coronavirus 2; SoC=standard of care; SpO_2=oxygen saturation; SST=serum separator tube; TNFα=tumor necrosis factor alpha.

[a] These visits will be performed even if a patient has already been discharged. If discharged prior to scheduled visit, in-person visits are preferred, but recognising that quarantine and other factors may limit the patient's ability to return to the clinic, these visits may be conducted by telephone or with a home visit by study staff. For visits conducted by telephone, it will not be possible to perform some scheduled assessments (eg, vital signs). The Day 29 assessments will also be performed, where possible, for patients who discontinue the study prematurely.

[b] Medical history includes estimated date and time of first symptoms and number of co-morbidities (eg, respiratory, cardiovascular, metabolic, malignancy, endocrine, gastrointestinal, immunologic, renal).

[c] Zilucoplan (32.4 mg) to be administered once a day for 14 days. However, if the patient is discharged before 14 days of treatment, zilucoplan should be stopped at time of discharge or 24 hours before discharge.

[d] Antibiotic prophylaxis decided by the investigator (eg, ciprofloxacin) for a maximum of 28 days starting with the first dose of zilucoplan (ie, during zilucoplan treatment and an additional 14 days after cessation of zilucoplan). If the patient is discharged prior to finishing antibiotic prophylaxis, it can be confirmed by telephone at Day 29 if prophylaxis was completed at home.

[e] Baseline assessments should be performed prior to study drug administration.

[f] Includes ordinal score, National Early Warning Score 2 (NEWS2), oxygen requirement, noninvasive or invasive ventilator requirement, including start and stop of low- or high-flow oxygen supply or of any form of ventilation etc.

[g] If done as part of SoC, blood gases results to be fully recorded with date and time.

[h] For parameters, see Master Protocol. D-dimer is required for this sub-study.

[i] Laboratory tests performed in the 48 hours prior to enrolment will be accepted for determination of eligibility.

[j] Any laboratory tests performed as part of routine clinical care within the specified visit window can be used for safety laboratory testing.

[k] Samples collected for immediate laboratory processing and frozen storage.

[l] Samples collected dependent on capacity of study centre, need for reduced study burden on staff, and potentially limited access to patients.

[m] Samples collected predose. These samples may be analysed for C5, C5a, sC5b-9, complement function tests and other exploratory biomarkers such as, but not limited to, inflammatory cytokines and additional complement components (eg, IL-1β, IL-1RA, IL-2, IL-8, IL-6, TNFα, GM-CSF, G-CSF, IP-10, MCP-1, MIP-1a, IFNγ, IL-10, C3, C3d, C4 MASP-1, factor B, fragment Bb).

[n] Samples to be collected on the day of discharge if prior to Day 15.

2.0 BACKGROUND/RATIONALE IN SUPPORT OF ZILUCOPLAN IN COVID-19

2.1 Rationale for C5A involvement in COVID-19

2.1.1 Background and Inflammation in COVID-19 infection

As of 24 March 2020, COVID-19 has been confirmed in 395,744 people worldwide, carrying a mortality of approximately 4.45%, compared with a mortality rate of less than 1% from influenza (1). The symptoms of COVID-19 vary from mild to acute respiratory distress syndrome (ARDS), the latter of which generally associated with deregulated immune cytokine production; however, little is currently known as to the interplay between the extent of clinical symptoms and the compositions of the immune responses. Some patient populations (eg, aged >70 years, patients with co-morbidities) are prone to develop more severe symptoms and require emergent medical interventions. Predictors of fatality from a recent retrospective study (2) included elevated ferritin (mean 1297.6 ng/mL in non-survivors versus 614.0 ng/mL in survivors; $p<0.001$) and interleukin (IL)-6 ($p<0.0001$), and patients with severe COVID-19 might have cytokine release syndrome (CRS). Pharmacotherapy targeted against the virus holds the greatest promise when applied early in the course of the illness, but its usefulness in advanced stages may be doubtful. Similarly, use of anti-inflammatory therapy applied too early may not be necessary and could even provoke viral replication such as in the case of corticosteroids. It appears that there are two distinct but overlapping pathological subsets, the first triggered by the virus itself and the second by the host response (Figure 1).

Figure 1 Escalating Phases of Disease Progression with COVID-19, with Associated Signs, Symptoms, and Potential Phase-Specific Therapies.

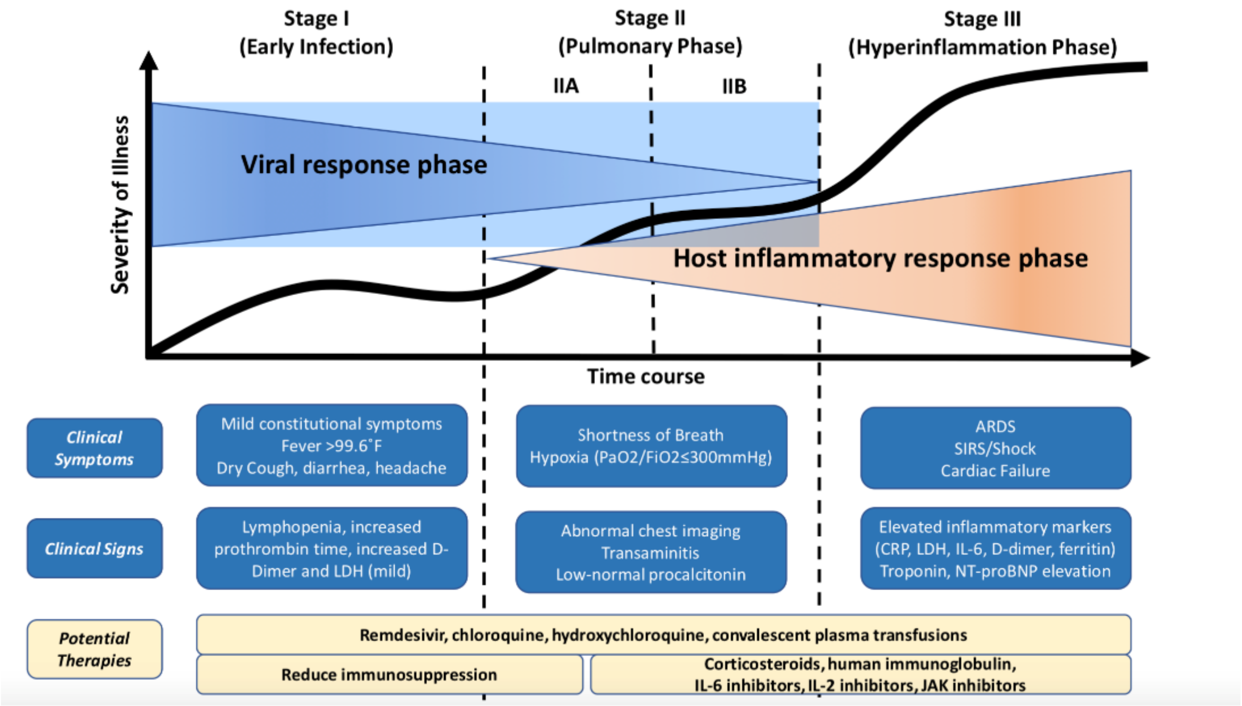

ARDS=Acute respiratory distress syndrome; CRP=C-reactive protein; IL=Interleukin; JAK=Janus kinase; LDH=Lactate dehydrogenase; SIRS=Systemic inflammatory response syndrome.
Source: Journal of Heart and Lung Transplantation. COVID-19 Illness in Native and Immunosuppressed States: A Clinical-Therapeutic Staging Proposal, Hasan K. Siddiqi, Mandeep R. Mehra.

Studies on immunological features of previous coronavirus epidemics show that robust and early T cell response played crucial roles in viral clearance (3). Currently, very limited information is available on the host innate immune status of SARS-CoV-2-infected patients. The description of increased total neutrophils, reduced total lymphocytes, and increased serum levels of IL-6 and of C-reactive protein suggest a strong inflammatory response; supported further by reports detecting abnormally high plasma levels of innate cytokines such as Interferon gamma-induced protein 10 (IP-10), monocyte chemoattractant protein 1 (MCP-1) and macrophage inflammatory protein (MIP)-1A or of high levels of pro-inflammatory cytokines (including IL-2, IL-7, IL-10, granulocyte colony-stimulating factor [G-CSF], IP-10, MCP-1, MIP-1A, and tumor necrosis factor alpha [TNFα]). The overall picture is similar to other high consequence coronavirus epidemics (SARS-CoV and MERS-CoV) and underlines the fact that leukocyte alterations, dysregulated interferon signalling, and CRS could be important in the pathogenesis of COVID-19.

Such severe clinical condition displayed by some of the patients affected with COVID-19 pneumonia are strongly reminiscent of previous and recent epidemic cases of respiratory failure associated to related coronarovirus such as the MERS-CoV, SARS-CoV. In more severe cases, infection can cause pneumonia, severe acute respiratory syndrome, kidney failure, and even

death (4). Death results from respiratory failure and is associated in a substantial percentage of patients with an inflammatory syndrome and a cytokine storm (2) with ARDS and features of macrophage activation syndrome/hemophagocytic lymphohistiocytosis that should be better defined.

There are currently no approved treatments for COVID-19.

2.1.2 Evidence that C5 Inhibition in COVID-19 Patients may prevent and possibly reverse Acute Respiratory Distress Syndrome (ARDS)

Current literature on coronavirus infection supports the importance of the humoral immune system in the control of infection. Early defence against coronavirus infection also includes a role for the “early” components of the complement pathway, in particular mannose-binding lectin (MBL).

In SARS-CoV, the surface S protein contains highly glycosylated, high-mannose structures. Case control studies of 569 patients with SARS and 1188 control subjects identified that MBL of the lectin pathway played a key role in the initiation of the innate immune response and that the distribution of MBL gene polymorphisms was significantly different between patients with SARS and control subjects, with a higher frequency of haplotypes associated with low or deficient serum levels of MBL in patients with SARS than in control subjects (5). In vitro studies demonstrated that MBL could bind SARS-CoV in a dose- and calcium-dependent fashion in vitro, suggesting that binding is through the carbohydrate recognition domains of MBL. These results suggest that MBL contributes to the first-line host defence against SARS-CoV and that MBL deficiency is a susceptibility factor for acquisition of SARS.

Although there is no evidence that “late” components (ie, C3 and C5) of the complement pathway are involved in the defence against SARS-CoV-2, aberrant activation of these components by virus-induced cellular damage is thought to promote broad tissue injury in coronavirus infection.

Although the complement system plays a role in innate immune defences against infectious agents, exaggerated activation can lead to severe tissue injury. Complement activation has long been known be associated with ARDS and sepsis. C5a, a potent anaphylatoxin, recruits neutrophils to the site of initial damage or infection and drives tissue damage by release of reactive oxygen species and tissue-degrading enzymes. C5a induces inflammatory cytokines such as IL-8, IL-6, IL-17, and TNFα from a variety of cell types and contributes to the cytokine storm associated with ARDS and sepsis (6) (7) (8). A report assessing C5a levels, polymorphonuclear granulocyte aggregation, and development of ARDS demonstrated a highly significant relationship between C5a positivity and ARDS, and this was significant even when eliminating patients with sepsis (9). Historical studies document the formation of soluble C5b-9 complex in plasma preceding the development of ARDS (10).

A broad role for complement in mediating ARDS-like tissue injury in mouse models has been demonstrated. In a mouse models of SARS-CoV infection, inhibition by knock-out of the late complement pathway (C3-/-) results in protection from disease (11). C3 deficiency eliminates the key component required for C5 activation and, as a result, animals are unable to generate C5a or C5b. In C3-deficient mice, weight loss and respiratory function were significantly improved relative to that of control mice (wild-type C3 background). Neither depletion of C4 nor factor B (upstream in the complement cascade to C3) resulted in similar protection. Lung pathology scores were reduced in the C3-/- mice relative to controls. Analysis of the cellular inflammatory response to mouse-adapted SARS-CoV (SARS-CoV MA15) infection revealed significant reductions in pathogenic inflammatory monocyte and neutrophil populations, both of which are implicated in human SARS-CoV pathology (12), indicating that complement signalling contributes to pulmonary disease and inflammatory cell recruitment. Finally, several pro-inflammatory cytokines, including IL-6, TNFα, IL-1a, and G-CSF were reduced in lungs of C3-/- mice relative to controls. Importantly, no change in viral loads were observed in C3-/- mice, underscoring that inhibiting the late/distal part of the complement cascade does not affect virus neutralization (11). In another mouse model, MERS-CoV infection-induced inflammation and pyroptosis was found to be mediated by C5a release and NLRP3 inflammasome activation. Blocking the C5a-C5aR1 axis with an anti-C5aR1 antibody when administered immediately prior to virus inoculation ameliorated lung inflammation (pyroptosis and macrophage infiltration) together with markers of systemic inflammation (11).

Further support for a role of complement in COVID-19 can be found in animal studies of avian influenza viruses. In humans, avian influenza H5N1 virus is associated with ARDS and is histopathologically-similar to patients infected with SARS-CoV. In mice, acute lung injury in avian influenza H5N1-infected mice appears to be complement-mediated. C3, C5b-9, and MBL were deposited in lung tissue, and complement receptors C3aR and C5aR were up-regulated. Treatment of H5N1 infected mice with a C3aR antagonist, an anti-C5a monoclonal antibody, or cobra venom factor to deplete complement C3 and C5 significantly alleviated lung inflammation and disease (13). Likewise, avian influenza H7N9 virus causes lung injury and ARDS resembling the disease in H5N1 and SARS-CoV infected patients. Infection of African Green Monkeys with H7N9 virus resulted in intense acute lung injury and systemic inflammatory response syndrome (SIRS) associated with complement activation. Treatment of H7N9-infected monkeys with an intravenously administered monoclonal antibody against C5a substantially attenuated disease, reducing lung histopathological injury and lung infiltration of macrophages and neutrophils. The treatment decreased the intensity of SIRS with minimal changes in body temperature markedly reduced plasma levels of inflammatory cytokines (13). These data indicate that excessive complement activation play an important role in viral-induced lung damage, and that complement inhibition at the level of C3 or C5 may be effective in ARDS.

Recent preliminary data assessing an anti-C5a monoclonal antibody in COVID-19 further support the use of C5 inhibition in this illness. Gao et al (2020) reported strong deposition of

complement components MBL, MASP-2, C4, C3, and C5-9 in the lung tissue of deceased COVID-19 patients, as well as a significant increase in serum C5a levels in patients with severe disease (14). As expected, activation was via the lectin pathway of complement (5). Two hospitalized patients were treated with repeat injections of an anti-C5a monoclonal antibody over approximately 13 days. Fever was reduced almost immediately, and rapid improvements in oxygen saturation, C-reactive protein, lymphocyte numbers, and clinical condition were observed.

Overall, these data support the conclusion that C5 inhibition to prevent the production of C5a and C5b may be effective in reducing inflammatory cytokine production, neutrophil activation, and progression to ARDS.

2.2 Dose selection for acute treatment of COVID-19

A dose of 32.4 mg of zilucoplan administered daily by subcutaneous injection has been selected for the acute treatment of patients with COVID-19. It is expected that this dose will result in complete complement inhibition over the dosing interval, which is anticipated to be necessary to achieve the optimal therapeutic response in patients with COVID-19, similar to the degree of inhibition required for patients with generalized myasthenia gravis (gMG) and paroxysmal nocturnal haemoglobinuria (PNH). This dose will result in the potential for a range of per unit weight doses to be received, from a minimum of 0.22 mg/kg daily (eg, patient weight 150 kg) to >0.6 mg/kg daily (eg, patient weight <54 kg).

The anticipated steady state exposure at 0.6 mg/kg provides a safety margin of approximately 1.3- and 1.6-fold (area under the drug concentration versus time curve and maximum plasma concentration, respectively) to the no-observed-adverse-effect level in the 39-week non-human primate toxicology study. In an ongoing Phase 1 study in healthy participants doses of 0.6 mg/kg have been administered with no major safety or tolerability concerns identified to date. In previous studies conducted in patients with gMG and PNH, there was no apparent difference with respect to the pattern and distribution of adverse events (AEs) or tolerability between the zilucoplan treated and the placebo groups. No dose response in the safety profile was seen between the 0.1 mg/kg and 0.3 mg/kg zilucoplan groups.

In previous studies conducted in patients with gMG and PNH, doses of 0.3 mg/kg showed superior efficacy, greater inhibition of the terminal complement pathway (see Figure 2), as compared with the 0.1 mg/kg dose level. The dose response seen in the clinical outcome measures in studies of gMG and PNH is consistent with the known pharmacodynamic effect of zilucoplan, that resulted in rapid, sustained and complete (97%) inhibition of the terminal complement pathway in all gMG patients receiving the 0.3 mg/kg dose while the 0.1 mg/kg group achieved only submaximal (88%) inhibition of the terminal complement pathway. Similarly, in Phase 2 studies in patients with PNH, the starting dose of 0.1 mg/kg daily did not consistently achieve complete inhibition of complement activity in the sheep red blood cell

(sRBC) lysis assay. The 0.3 mg/kg daily dose, by contrast, consistently achieved complete inhibition in the sRBC lysis assay (≥95% inhibition at trough; see Figure 2) and reduced lactate dehydrogenase (LDH) to levels similar to those observed in patients receiving eculizumab.

Therefore, the 0.3 mg/kg daily nominal dose level previously-tested in PNH and myasthenia gravis has again been selected for use in this study with COVID-19 patients. Although previous trials of zilucoplan implemented weight-based dosing with 3 weight brackets, only the fixed dose from the highest weight bracket (32.4 mg) will be administered in this study, in order to rapidly and efficiently achieve complete complement inhibition, and to account for any reduction in perfusion or cardiac output in the setting of critical illness and/or cardiovascular compromise that may be expected in COVID-19 patients.

Figure 2 Mean Complement Activity as Measured by Sheep Red Blood Cell Assay (% Haemolysis).

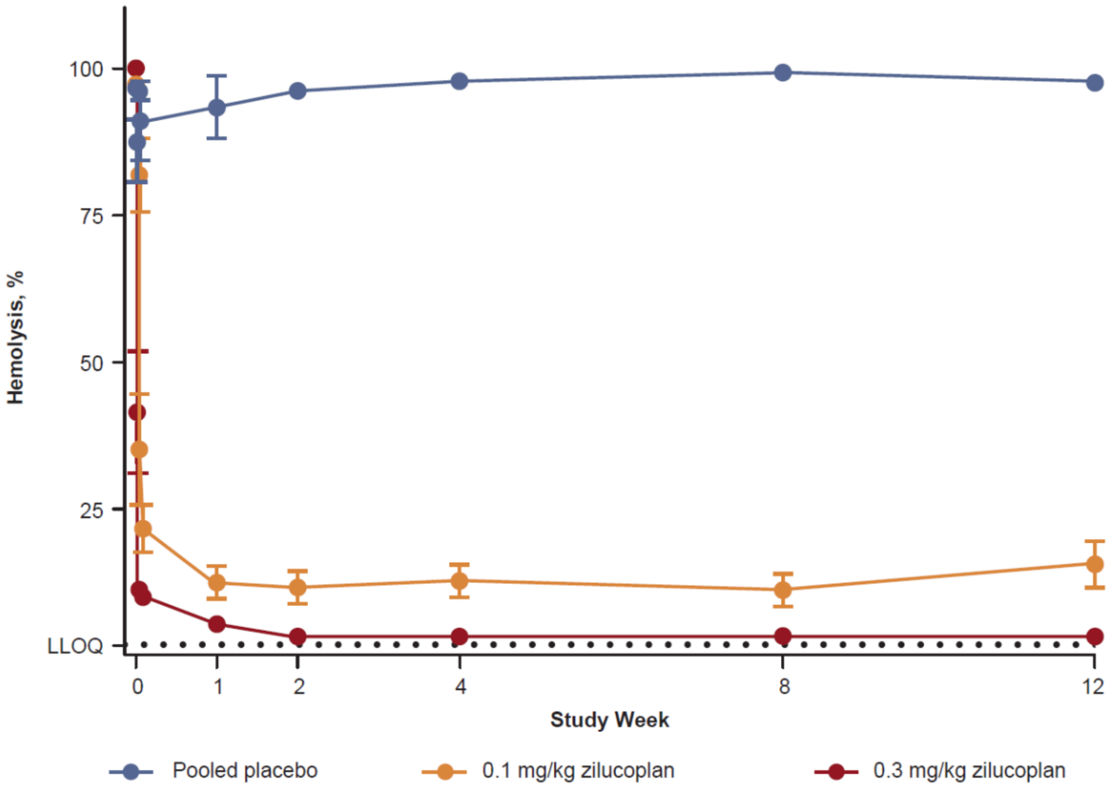

LLOQ=lower limit of quantification

3.0 STUDY POPULATION

3.1 Enrolment and Screening

Enrolment and screening will continue until the required number of patients outlined in the Master Protocol is reached. Enrolment for this sub-protocol will follow all enrolment procedures as outlined in the Master Protocol.

3.2 Eligibility Criteria

Overall inclusion and exclusion criteria are presented in Sections 5.1 and 5.2 of the Master Protocol, respectively. The following sections detail variations to those criteria that are specific to this sub-protocol.

3.2.1 Inclusion Criteria

Inclusion criterion 3 from the Master Protocol, related to contraception, will be replaced with the following:

3. a) Male subjects:

- A male subject must agree to use contraception as detailed in Appendix 2 of this protocol during the treatment period and for at least 6 weeks after the last dose of study treatment and refrain from donating sperm during this period.

b) Female subjects:

- A female subject is eligible to participate if she is not pregnant (see Appendix 2), not breastfeeding, and at least 1 of the following conditions applies:

  i) Not a woman of childbearing potential (WOCBP) as defined in Appendix 2.
  OR
  ii) A WOCBP who agrees to follow the contraceptive guidance in Appendix 2 during the treatment period and for at least 6 weeks after the last dose of study treatment.

An additional inclusion criterion that is specific for this sub-protocol:

X1. Antibiotic prophylaxis: PLEASE NOTE that, according to Section 4.1.1.1, all patients must take antibiotic prophylaxis concomitantly, starting with the first dose of zilucoplan.

3.2.2 Exclusion Criteria

An additional exclusion criterion that is specific for this sub protocol:

X1. Participants with unresolved or suspected infection with *Neisseria meningitidis*, or a past history of *Neisseria meningitidis* (eg, in a complement deficient patient), should not receive treatment with zilucoplan.

4.0 STUDY ASSESSMENTS AND PROCEDURES

In addition to the study assessments and procedures described in Section 8.0 of the Master Protocol, assessments specific to the sub-protocol will be performed as described in the following sections. The Schedule of Activities for this sub-protocol is presented in Section 1.2.

4.1 Safety Assessments

4.1.1 Adverse Events

4.1.1.1 Adverse Events with zilucoplan

To date zilucoplan has shown a favourable safety and good tolerability profile across all studies in healthy participants and patients with gMG, PNH, immune mediated necrotising myopathy (IMNM), and renal impairment. No major safety risks have been identified to date, with over 100 patient years of exposure across and >40,000 injections administered in clinical trials. A difference in the overall AE profiles was seen between the clinical trials in different populations: few AEs occurred in healthy controls and stable renally impaired patients; the AE profile in PNH reflected the haemolytic anaemia characteristic of the underlying disease; and in the gMG population, many AEs were reflective of multiple comorbidities, longstanding use of corticosteroid and immunosuppressive therapies, and the more advanced age of this population.

Although no meningococcal infections were identified with zilucoplan treatment to date, it is well established that inhibition of complement C5 and the terminal complement pathway increases the susceptibility to infection with encapsulated bacteria, in particular *Neisseria meningitidis*. This risk is also described in the prescribing information for the approved complement C5 inhibitor eculizumab (Soliris® USPI 2017).

Given the increased risk for *Neisseria meningitidis* infection with complement C5 inhibition or deficit, patients who have received zilucoplan in ambulatory clinical trials were required to have documentation of *Neisseria meningitidis* vaccination (and booster if appropriate) prior to treatment initiation. In addition, while on zilucoplan, subjects were monitored closely for signs and symptoms of *Neisseria meningitidis* infection, including self- monitoring based on detailed instruction about signs and symptoms of possible meningococcal infections.

Considering the acuity of the indication and short duration of treatment (maximum of 14 days), vaccination and induction of humoral immunity prior to initiation of zilucoplan therapy is not feasible in the context of COVID-19. Therefore, the risk of Neisserial infection must be mitigated by concomitant administration of antibiotic prophylaxis (eg, ciprofloxacin) decided by the investigator for a maximum of 28 days starting with the first dose of zilucoplan (ie, during zilucoplan treatment and an additional 14-day period after cessation of zilucoplan).

4.1.1.2 Adverse Events of Special Interest (AESI)

No AESIs have been defined for zilucoplan in this protocol.

4.1.2 Clinical Safety Laboratory Assessments

See the Master Protocol for the list of clinical laboratory tests to be performed for this sub-protocol, and the Schedule of Activities (Section 1.2) for the timing and frequency.

4.2 Pharmacokinetic Assessments

Plasma samples (from whole blood collection) will be collected for measurement of plasma concentrations of zilucoplan, as specified in the Schedule of Activities (Section 1.2). Instructions for the collection and handling of biological samples will be provided in the Laboratory Manual.

Samples collected for analyses of zilucoplan concentration in plasma may also be used to evaluate safety or efficacy aspects related to concerns arising during or after the study.

4.3 Immunogenicity Assessments

Serum samples (from whole blood collection) will be collected for the evaluation of the presence or absence of anti-drug antibody (ADA) response as specified in the Schedule of Activities. Instructions for the collection and handling of biological samples will be provided in the Laboratory Manual.

Samples collected for analyses of ADA in serum may also be used to evaluate safety or efficacy aspects related to concerns arising during or after the study.

4.4 Pharmacodynamic Assessments

Blood samples for pharmacodynamic analysis will be collected as specified in the Schedule of Activities (Section 1.2). The samples may be analysed for C5, C5a, sC5b-9, complement function tests and other exploratory biomarkers, such as, but not limited to, C3, C3d, C4 MASP-1, factor B and fragment Bb, and other complement nonrelated biomarkers. Additionally, these blood samples may be used for analysis of inflammatory cytokines and additional complement components which may include, but are not limited to, IL-1β, IL-1RA, IL-2, IL-8, IL-6, TNFα, granulocyte-macrophage colony-stimulating factor, G-CSF, IP-10, MCP-1, MIP-1a, interferon gamma, and IL-10.

Instructions pertaining to sample collection, processing, storage, labelling, and shipping are provided in the Laboratory Manual for this study. All collected samples may be also used for research purposes focusing on method development and assay development. Collection of these samples will enable evaluation of biomarkers relative to disease biology and progression, study medication treatment and response, and/or mechanism of action of the study medication

treatment. The samples will be collected and stored from all participants in this study to support potential future exploratory biomarker research.

5.0 STUDY TREATMENT

5.1 Treatment Plan

Patients will continue taking treatment once daily as a subcutaneous injection for the planned treatment duration. Patients may discontinue treatment early at the discretion of the investigator.

5.2 Study Drug Administration

The investigational drug product will be provided in prefilled syringes containing 32.4 mg of zilucoplan (0.81 mL) for subcutaneous injection in the abdomen (preferred site), thigh, or upper arm. This dose is equivalent to that administered to the highest weight bracket in prior weight-based dosing regimens and is expected to achieve rapid, profound, and sustained complement inhibition with acceptable safety and tolerability.

Zilucoplan should be stored at 2°C to 8°C at the study site. Once dispensed, zilucoplan may be stored at room temperature (20°C to 25°C [68°F to 77°F]) for up to 45 days protected from sources of heat, light, and damage. Storage of zilucoplan outside of room temperatures should be avoided.

5.3 Dose Modifications and Toxicity Management

To date, zilucoplan has shown good tolerability and a favourable safety profile across studies in healthy participants and patients with renal impairment, PNH and MG. No major safety risks were identified to date.

No dose modifications are anticipated, stopping zilucoplan will be left to the decision of the investigator.

5.4 Concomitant Medications

To mitigate the risk of Neisserial infection, concomitant administration of antibiotic prophylaxis (eg, ciprofloxacin) decided by the investigator is required for a maximum of 28 days starting with the first dose of zilucoplan (ie, during zilucoplan treatment and an additional 14 days after cessation of zilucoplan).

If the patient is discharged prior to finishing antibiotic prophylaxis, it can be confirmed by telephone at Day 29 if prophylaxis was completed at home.

Additional antibiotics can be administered as per investigator's judgment.

5.4.1 Rescue Medicine

No available rescue medications.

5.5 Study Drug Information

Zilucoplan (RA101495) is a 15-amino acid macrocyclic peptide complement inhibitor designed for the treatment of conditions in which inappropriate activation of complement C5 has been demonstrated to play a role. Zilucoplan binds to C5 with high affinity and prevents its cleavage by C5 convertases into the cleavage products C5a and C5b. Inhibition of C5 cleavage prevents the downstream assembly and cytolytic activity of the membrane attack complex (MAC). Zilucoplan binds to the domain of C5 which corresponds to C5b. Should any C5b be generated, it will be blocked from binding to C6 by zilucoplan, thereby preventing the subsequent assembly of the MAC (C5b-9).

Zilucoplan is being developed by Ra Pharmaceuticals, Inc. ("Ra Pharma"), a member of the UCB group, as a potential treatment for patients with gMG, IMNM, PNH, amyotrophic lateral sclerosis (ALS), and other complement-mediated diseases.

6.0 REFERENCES

1. **World Health Organization.**

2. *COVID-19: consider cytokine storm syndromes and immunosuppression.* **Mehta P, McAuley DF, Brown M, Sanchez E, Tattersall RS, Manson JJ and HLH Across Speciality Collaboration, UK.** s.l. : Lancet, 2020, Vols. 395(10229):1033-1034.

3. *Immune responses in COVID-19 and potential vaccines: Lessons learned from SARS and MERS epidemic.* **Prompetchara E, Ketloy C, Palaga T.** s.l. : Asian Pac J Allergy Immunol, 2020, Vols. 38(1):1-9. doi: 10.12932/AP-200220-0772.

4. *Clinical features of patients infected with 2019 novel coronavirus in Wuhan, China.* **Huang C, Wang Y, Li X, Ren L, Zhao J, Hu Y, et al.** s.l. : Lancet, 2020, Vols. 395(10223):497-506. doi: 10.1016/S0140-6736(20)30183-5.

5. *Mannose-Binding Lectin in Severe Acute Respiratory Syndrome Coronavirus Infection.* **Ip et al.** s.l. : J. Infect. Dis., 2005, Vols. 191:1697-1704. https://doi.org/10.1086/429631.

6. **Bosmann, M and Ward, PA.** Role of C3, C5 and Anaphylatoxin Receptors in Acute Lung Injury and in Sepsis. [book auth.] G. Hajishengallis J. D. Lambris. *Current Topics in Innate Immunity II. Advances in Experimental Medicine and Biology, vol 946.* s.l. : Springer. 147-159 https://doi.org/10.1007/978-1-4614-0106-3_9, 2011.

7. *Neutrophils in the initiation and resolution of acute pulmonary inflammation: understanding biological function and therapeutic potential.* **Potey PM, Rossi AG, Lucas CD, Dorward DA.** s.l. : J. Pathol., 2019, Vols. 247(5):672-685 . https://doi.org/10.1002/path.5221.

8. *Roles of the neutrophil and other mediators in adult respiratory distress syndrome.* **Swank DW, Moore SB.** s.l. : Mayo Clin Proc, 1989, Vols. 64(9):1118-32.

9. *Association of complement activation and elevated plasma-C5a with adult respiratory distress syndrome. Pathophysiological relevance and possible prognostic value.* **Hammerschmidt DE, Weaver LJ, Hudson LD, Craddock PR, Jacob HS.** s.l. : Lancet, 1980, Vols. 1(8175):947-9.

10. *Accentuated formation of the terminal C5b-9 complement complex in patient plasma precedes development of the adult respiratory distress syndrome.* **Langlois PF, Gawryl MS.** s.l. : Am. Rev. Resp. Dis., 1988, Vols. 138(2):368-75.

11. *Complement Activation Contributes to Severe Acute Respiratory Syndrome Coronavirus Pathogenesis.* **Gralinski LE, Sheahan TP, Morrison TE, Menachery VD, Jensen K, Leist SR, Whitmore A, Heise MT, Baric RS.** s.l. : mBio, 2018, Vol. 9(5). doi: 10.1128/mBio.01753-18.

12. *The pathology and pathogenesis of experimental severe acute respiratory syndrome and influenza in animal models.* **van den Brand JM, Haagmans BL, van Riel D, Osterhaus AD, Kuiken T.** s.l. : J Comp Pathol, 2014, Vols. 151(1):83-112. http://dx.doi.org/10.1016/j.jcpa.2014.01.004.

13. *Inhibition of complement activation alleviates acute lung injury induced by highly pathogenic avian influenza H5N1 virus infection.* **Sun, S. et al.** s.l. : Am. J. Respir. Cell. Mol. Biol., 2013, Vols. 49 (2):221-230. doi: 10.1165/rcmb.2012-0428OC.

14. *Highly pathogenic coronavirus N protein aggravates lung injury by MASP-2-mediated complement over-activation.* **Gao, T. et al.** s.l. : medRxiv, 2020. https://doi.org/10.1101/2020.03.29.20041962.

7.0 APPENDICES

Appendix 1 Abbreviations

Abbreviation	Definition
ADA	Anti-drug antibody
AE	Adverse event
AESI	Adverse event of special interest
ARDS	Acute respiratory distress syndrome
C	Component
COVID-19	Coronavirus disease 2019
CRO	Contract research organization
CRP	C-reactive protein
CRS	Cytokine release syndrome
G-CSF	Granulocyte colony-stimulating factor
GM-CSF	Granulocyte-macrophage colony-stimulating factor
gMG	Generalized myasthenia gravis
IL	Interleukin
IMNM	Immune mediated necrotising myopathy
IP-10	Interferon gamma induced protein 10
JAK	Janus kinase
LDH	Lactate dehydrogenase
LLOQ	Lower limit of quantification
MAC	Membrane attack complex
MBL	Mannose-binding lectin
MCP-1	Monocyte chemoattractant protein 1
MIP	Macrophage inflammatory protein
PNH	Paroxysmal nocturnal haemoglobinuria
SARS-CoV-2	Severe acute respiratory syndrome coronavirus 2
SIRS	Systemic inflammatory response syndrome
sRBC	Sheep red blood cell
TNFα	Tumor necrosis factor alpha
WOCBP	Woman of childbearing potential

Appendix 2 Contraceptive Guidance

Definitions:

Woman of Childbearing Potential (WOCBP)

A woman is considered fertile following menarche and until becoming postmenopausal unless permanently sterile (see below).

Women in the following categories are not considered WOCBP

2. Premenarchal
3. Premenopausal female with 1 of the following:
   a) Documented hysterectomy.
   b) Documented bilateral salpingectomy.
   c) Documented bilateral oophorectomy.
   Note: Documentation can come from the study center personnel's: review of the subject's medical records, medical examination, or medical history interview.
4. Postmenopausal female:
   a) A postmenopausal state is defined as no menses for 12 months without an alternative medical cause. A high follicle stimulating hormone (FSH) level in the postmenopausal range may be used to confirm a postmenopausal state in women not using hormonal contraception or hormonal replacement therapy (HRT). However, in the absence of 12 months of amenorrhea, a single FSH measurement is insufficient.
   b) Females on HRT and whose menopausal status is in doubt will be required to use 1 of the non-estrogen hormonal highly effective contraception methods if they wish to continue their HRT during the study. Otherwise, they must discontinue HRT to allow confirmation of postmenopausal status before study enrollment.

Contraception Guidance

Male subjects

- Male subjects with female partners of childbearing potential are eligible to participate if they agree to ONE of the following:
  - Are abstinent from penile-vaginal intercourse as their usual and preferred lifestyle (abstinent on a long-term and persistent basis) and agree to remain abstinent.
  - Agree to use a male condom plus partner use of a contraceptive method with a failure rate of <1% per year as described in the table below when having penile-vaginal intercourse with a woman of childbearing potential who is not currently pregnant.
- In addition, male subjects must refrain from donating sperm for the duration of the study and for 6 weeks after the last dose of study treatment.

- Male subjects with a pregnant or breastfeeding partner must agree to remain abstinent from penile-vaginal intercourse or use a male condom during each episode of penile penetration for the duration of the study and for 6 weeks after the last dose of study treatment.

Female subjects

Female subjects of childbearing potential are eligible to participate if they agree to use a highly effective method of contraception consistently and correctly as described in the table below.

Highly Effective Contraceptive Methods

**Highly Effective Contraceptive Methods That Are User Dependent** [a]<br>*Failure rate of <1% per year when used consistently and correctly.*
Combined (oestrogen and progestogen containing) hormonal contraception associated with inhibition of ovulation[b]<br>• Oral.<br>• Intravaginal.<br>• Transdermal.
Progestogen only hormonal contraception associated with inhibition of ovulation<br>• Oral.<br>• Injectable.
**Highly Effective Methods That Are User Independent** [a]
Implantable progestogen only hormonal contraception associated with inhibition of ovulation[b]<br>• Intrauterine device (IUD).<br>• Intrauterine hormone-releasing system (IUS).<br>Bilateral tubal occlusion.
**Vasectomized partner**<br>*A vasectomized partner is a highly effective birth control method provided that the partner is the sole male sexual partner of the WOCBP and the absence of sperm has been confirmed. If not, an additional highly effective method of contraception should be used.*
**Sexual abstinence**<br>*Sexual abstinence is considered a highly effective method only if defined as refraining from heterosexual intercourse during the entire period of risk associated with the study treatment. The reliability of sexual abstinence needs to be evaluated in relation to the duration of the study and the preferred and usual lifestyle of the subject.*
NOTES:<br>[a] Typical use failure rates may differ from those when used consistently and correctly. Use should be consistent with local regulations regarding the use of contraceptive methods for subjects participating in clinical studies.<br>[b] Hormonal contraception may be susceptible to interaction with the study treatment, which may reduce the efficacy of the contraceptive method. In this case, 2 highly effective methods of contraception should be utilized during the treatment period and for at least 6 weeks after the last dose of study treatment.

Appendix 3 Signature of Investigator

PROTOCOL TITLE: A Multicentre, Seamless, Phase 2 Adaptive Randomisation Platform Study to Assess the Efficacy and Safety of Multiple Candidate Agents for the Treatment of COVID-19 in Hospitalised Patients

SUB-PROTOCOL NO: ACCORD-2-006

SUB-PROTOCOL FOR CANDIDATE AGENT ZILUCOPLAN

VERSION: Original Protocol

This sub-protocol is a confidential communication of the Sponsor. I confirm that I have read this sub-protocol, I understand it, and I will work according to this sub-protocol, in conjunction with the Master Protocol for the overall platform study. I will also work consistently with the ethical principles that have their origin in the Declaration of Helsinki and that are consistent with Good Clinical Practices and the applicable laws and regulations. Acceptance of this document constitutes my agreement that no unpublished information contained herein will be published or disclosed without prior written approval from the Sponsor.

Instructions to the Investigator: Please SIGN and DATE this signature page. PRINT your name, title, and the name of the study centre in which the study will be conducted. Return the signed copy to the CRO/Sponsor.

I have read this sub-protocol in its entirety and agree to conduct this part of the study accordingly:

Signature of Investigator: ___________________________ Date: ________

Printed Name: ____________________________

Investigator Title: ____________________________

Name/Address of Centre: ____________________________

____

____
