# Supplementary material for: ACCORD: A Multicentre, Seamless, Phase 2 Adaptive Randomisation Platform Study to Assess the Efficacy and Safety of Multiple Candidate Agents for the Treatment of COVID-19 in Hospitalised Patients: A structured summary of a study protocol for a randomised controlled trial
Source: Trials. 2020 Jul 31;21:691. doi: 10.1186/s13063-020-04584-9 (PMC7393340; doi:10.1186/s13063-020-04584-9)
Supplement: Supplementary file 1 — Additional file 1. [file 13063_2020_4584_MOESM1_ESM.zip › ACCORD-2-001 Master ProtocolR0.pdf]

TITLE PAGE

Protocol Title: ACCORD-2: A Multicentre, Seamless, Phase 2 Adaptive Randomisation Platform Study to Assess the Efficacy and Safety of Multiple Candidate Agents for the Treatment of COVID-19 in Hospitalised Patients

Master Protocol Number: ACCORD-2-001

Product: Multiple candidate agents

Study Phase: 2

Sponsor Name: University Hospital Southampton NHS Foundation Trust

**Legal Registered Address:**	**Southampton General Hospital**<br>**Level E, Laboratory & Pathology Block, SCBR - MP138**<br>**Tremona Road**<br>**Southampton SO16 6YD, UK**

**Regulatory Agency Identifying Number(s):**	**EudraCT 2020-001736-95**
**IRAS Number:**	**282769**
**RHM Number:**	**MED1711**

Date of Protocol: 24 April 2020

Version: Protocol Amendment 01, Final

Chief Investigator Signatory:

I have read this protocol in its entirety and agree to conduct the study accordingly:

**Professor Tom Wilkinson MA Cantab MBBS PhD FRCP**<br>**Professor of Respiratory Medicine and Honorary NHS Consultant Physician**	**Date**

TABLE OF CONTENTS

TABLE OF TABLES 6

TABLE OF FIGURES 7

PROTOCOL AMENDMENT HISTORY 8

1.0 PROTOCOL SUMMARY 9
- **1.1 Synopsis** 9
- **1.2 Schema** 14
- **1.3 Example Schedule of Activities** 15

2.0 INTRODUCTION 19
- **2.1 Study Rationale** 19
- **2.2 Background** 19
- **2.3 Benefit/Risk Assessment** 20

3.0 OBJECTIVES AND ENDPOINTS 21

4.0 STUDY DESIGN 23
- **4.1 Overall Design** 23
- **4.2 Scientific Rationale for Study Design** 25
- **4.3 Justification for Dose** 25
- **4.4 End of Study Definition** 25

5.0 STUDY POPULATION 26
- **5.1 Inclusion Criteria** 26
- **5.2 Exclusion Criteria** 26
- **5.3 Lifestyle Considerations** 27
- **5.4 Screen Failures** 27

6.0 STUDY TREATMENT 28
- **6.1 Study Treatment(s) Administered** 28
- **6.2 Preparation/Handling/Storage/Accountability** 28
- **6.3 Measures to Minimise Bias: Randomisation and Blinding** 29
- **6.4 Study Treatment Compliance** 29
- **6.5 Concomitant Therapy** 29
  - 6.5.1 Rescue Medicine 30
- **6.6 Dose Modification** 30
- **6.7 Treatment after the End of the Study** 30

7.0 DISCONTINUATION OF STUDY TREATMENT AND PATIENT DISCONTINUATION/WITHDRAWAL 31
7.1 Discontinuation of Study Treatment 31
7.2 Patient Discontinuation/Withdrawal from the Study 31
7.3 Lost to Follow-up 31

8.0 STUDY ASSESSMENTS AND PROCEDURES 33
8.1 Efficacy Assessments 34
8.1.1 Improvement on the 9-Point Scale 34
8.1.2 Other Efficacy Assessments 35
8.2 Safety Assessments 35
8.2.1 Physical Examinations 35
8.2.2 Vital Signs and Blood Gases 35
8.2.3 Clinical Safety Laboratory Assessments 36
8.3 Virologic Load 36
8.4 Adverse Events 36
8.4.1 Time Period and Frequency for Collecting AE and SAE Information 36
8.4.2 Method of Detecting AEs and SAEs 37
8.4.3 Follow-up of AEs and SAEs 37
8.4.4 Regulatory Reporting Requirements for SAEs 37
8.4.5 Pregnancy 38
8.4.6 Adverse Events of Special Interest 38
8.4.7 Disease-Related Events and/or Disease-Related Outcomes Not Qualifying as Adverse Events or Serious Adverse Events 38
8.5 Treatment of Overdose 38
8.6 Pharmacokinetics 38
8.7 Pharmacodynamics 39
8.8 Immunology 39
8.9 Genomics 39
8.10 Serology Research (Host Response) 40

9.0 STATISTICAL CONSIDERATIONS 40
9.1 Design Overview 40
9.2 Statistical Hypotheses 40
9.3 Sample Size Determination 41
9.4 Populations for Analyses 43
9.5 Statistical Analyses 43
9.5.1 Efficacy Analyses 44
9.5.2 Safety Analyses 44
9.5.3 Other Analyses 45
9.5.4 Missing Data 45
9.6 Interim Analyses 46
9.7 Review Committees 46
9.7.1 Steering Committee (Scientific Review Committee) 46
9.7.2 Independent Data and Safety Monitoring Committee 46

10.0 REFERENCES....48

11.0 APPENDICES....49
Appendix 1 Abbreviations....50
Appendix 2 Regulatory, Ethical, and Study Oversight Considerations....52
Protocol Compliance....52
Regulatory and Ethical Considerations....52
Financial Disclosure....53
Indemnity....53
Informed Consent Process....53
Data Protection....54
Administrative Structure....55
Dissemination of Clinical Study Data....55
Data Quality Assurance....55
Source Documents....56
Study and Study Centre Closure....56
Publication Policy....57
Appendix 3 Clinical Laboratory Tests....58
Appendix 4 Adverse Events: Definitions and Procedures for Recording, Evaluating, Follow-up, and Reporting....60
Appendix 5 Collection of Pregnancy Information....64
Appendix 6 Genetics....66
Appendix 7 Signature of Investigator....67

TABLE OF TABLES

Table 1 Study Objectives and Endpoints 21
Table 2 Sample Size for 80% and 90% Power for Time to Improvement, Discharge from Hospital, or Fit for Discharge Using Log-rank Test for a Hazard Ratio of 1.6 in Treatment Arm to Standard of Care 42
Table 3 Analysis Sets 43
Table 4 Efficacy Analyses 44
Table 5 Protocol-required Safety Laboratory Assessments 59

TABLE OF FIGURES

Figure 1 Study Schema..14

PROTOCOL AMENDMENT HISTORY

Protocol amendment 01 (dated 24 April 2020) replaces the original Master Protocol (dated 22 April 2020).

The amendment incorporates the following main changes:

- RHM number added to title page.
- Removed requirement that temperature needs to be taken orally.
- Rearranged wording in inclusion criterion #3 to make it clearer that the criterion applies to patients of all genders. Also added the clarification that a vasectomised partner would be considered an appropriate birth control method.
- Added inclusion criterion regarding lactating women.
- Added exclusion criterion regarding tuberculosis.
- Removed text about rescreening due to hypokalaemia, hypomagnesemia, or hypocalcaemia.
- Clarified that the Sponsor and the candidate agent owner only need to exchange important safety findings regarding the candidate agent (not all findings).
- Removed text about separate informed consent forms for the Master Protocol and the sub-protocol (there will only be one informed consent form to sign).
- Study monitors will perform ongoing source data review (not source data verification).
- Clarified that all clinical laboratory tests will be performed at a local laboratory (there will be no central laboratory for these tests).
- Assessment of intensity details amended, as Common Terminology Criteria for Adverse Events (CTCAE) grades will be used.

1.0 PROTOCOL SUMMARY

1.1 Synopsis

Protocol Title: ACCORD-2: A Multicentre, Seamless, Phase 2 Adaptive Randomisation Platform Study to Assess the Efficacy and Safety of Multiple Candidate Agents for the Treatment of COVID-19 in Hospitalised Patients

Rationale:

There are currently no approved therapeutic agents available to treat coronaviruses such as severe acute respiratory syndrome coronavirus 2 (SARS-CoV-2), the causative agent of COVID-19 disease, and there is an urgent public health need for rapid development of such interventions. This adaptive platform study is designed to rapidly assess multiple candidate agents as treatments for COVID-19. Candidate drugs that are initially assessed as being efficacious will be moved from an evaluation (pilot) stage to a confirmatory stage, with candidate agents being added to and removed from the study on an ongoing basis, depending on the results of their evaluation. Patients to be included in the study will be hospitalised and may require either supplemental oxygen, noninvasive ventilation or high-flow oxygen devices.

Objectives and Endpoints

Objectives	Endpoints
Primary	
• Stage 1: To evaluate the efficacy of candidate agents as add-on therapies to standard of care (SoC) in patients hospitalised with COVID-19 in a screening stage.<br>• Stage 2: To confirm the efficacy of identified efficacious candidate agents in patients hospitalised with COVID-19 in an expansion stage.	• Time to clinical improvement of at least 2 points (from randomisation) on a 9-point category ordinal scale, live discharge from the hospital, or considered fit for discharge (a score of 0, 1, or 2 on the ordinal scale), whichever comes first, by Day 29 (this will also define the “responder” for the response rate analyses).<br>9-point category ordinal scale:<br>0. Uninfected, no clinical or virological evidence of infection<br>1. Ambulatory, no limitation of activities<br>2. Ambulatory, limitation of activities<br>3. Hospitalised – mild disease, no oxygen therapy<br>4. Hospitalised – mild disease, oxygen by mask or nasal prongs<br>5. Hospitalised – severe disease, noninvasive ventilation or high-flow oxygen<br>6. Hospitalised – severe disease, intubation and mechanical ventilation<br>7. Hospitalised – severe disease, ventilation and additional organ support – vasopressors, renal replacement therapy (RRT), extracorporeal membrane oxygenation (ECMO)<br>8. Death

Secondary	
• To evaluate the ability to prevent deterioration according to the ordinal scale by 1, 2, or 3 points	• The proportion of patients not deteriorating according to the ordinal scale by 1, 2, or 3 points on Days 2, 8, 15, 22, and 29.
• To evaluate the number of oxygen-free days.	• Duration (days) of oxygen use and oxygen-free days.
• To evaluate ventilator-free days and incidence and duration of any form of new ventilation use.	• Duration (days) of ventilation and ventilation-free days.<br>• Incidence of any form of new ventilation use and duration (days) of new ventilation use.
• To evaluate SARS-CoV-2 viral load.	• Qualitative and quantitative polymerase chain reaction (PCR) determination of severe acute respiratory syndrome coronavirus 2 (SARS-CoV-2) in oropharyngeal/nasal swab while hospitalised on Days 1, 3, 5, 8, 11, 15, and (optional) Day 29
• To evaluate response rate (see primary endpoint for definition of responder).	• Response rate (number and %) by treatment arm at Days 2, 8, 15, 22, and 29.
• To evaluate time to discharge.	• Time to live discharge from the hospital.
• To evaluate overall mortality.	• Mortality at Days 15, 29, and 60.<br>• Time from treatment start date to death.
• Change in the ratio of the oxygen saturation to fraction of inspired oxygen concentration (SpO_2/FiO_2),	• SpO_2/FiO_2, measured daily from randomisation to Day 15, hospital discharge, or death
• To evaluate the safety of candidate agents as add-on therapy to SoC in patients with COVID-19.	• Physical examination.<br>• Clinical laboratory examinations.<br>• Vital signs (blood pressure/heart rate/temperature/respiratory rate).<br>• Adverse events.
• To evaluate intensive care unit (ICU) and hospitalisation length.	• Duration (days) of ICU and hospitalisation.
• To evaluate National Early Warning Score 2 (NEWS2).	• NEWS2 assessed daily while hospitalised and on Days 15 and 29.<br>• Time to a NEWS2 of ≤2, maintained for at least 24 hours.

Exploratory	
• To evaluate SARS-CoV-2 viral load.	• Qualitative and quantitative PCR determination of SARS-CoV-2 in blood and saliva (while hospitalised) on Days 1, 3, 5, 8, 11, 15, and (optional) Day 29 (may be become a secondary endpoint once the assays are available).
• To collect samples for translational research on host and viral genomics, serum antibody production, COVID-19 diagnostics, and validation of laboratory testing methods.	• Analysis of samples collected at baseline prior to treatment and at specific time points.

Overall Design:

ACCORD-2 is a seamless, Phase 2, adaptive, randomisation platform study, designed to rapidly test candidate agents in the treatment of COVID-19 disease. The study will include hospitalised adult patients (≥18 years) who have infection with SARS-CoV-2, the virus that causes COVID-19, as confirmed by laboratory tests and/or point of care tests. For inclusion, patients will need to have clinical status of Grade 3 (hospitalised – mild disease, no oxygen therapy) to Grade 5 (hospitalised – severe disease, noninvasive ventilation or high-flow oxygen), as defined by a 9-point ordinal scale.

This study will aim to identify efficacious candidate agents for treatment of COVID-19 disease. These candidate agents may include, but will not be limited to, anti-virals, human plasma-derived agents, or immunomodulatory agents. Experimental (first-in-human) agents will not be considered as candidate agents for ACCORD-2, but will instead be considered for inclusion in the separate, but linked, ACCORD-1 Phase 1/2 platform study, which will first determine the dose and assess early activity and safety signals for later consideration for inclusion into ACCORD-2. The ACCORD-2 candidate agents evaluated will include those intended as a treatment for SARS-CoV-2 infection; the study design and/or inclusion and exclusion criteria may subsequently be revised (using a protocol amendment) or a separate protocol may be initiated to include agents intended to prevent COVID-19 disease.

This Master Protocol outlines the overall structure of the study, including the population, inclusion and exclusion criteria, randomisation scheme, primary, secondary, and exploratory outcomes, study design, statistical methodology, and planned analyses that are common for all candidate agents to be tested. The Master Protocol is structured such that multiple candidate agents from different pharmaceutical companies can be evaluated simultaneously. The plan is to add candidate agents as they are identified, and to remove therapies once they have completed their evaluation, with the control group for a candidate agent including only patients randomised during the period in which the candidate agent group was randomised, with patients being randomised equally (ie, 1:1:1…) to the sub-protocols with inclusion/exclusion criteria that they meet.

Sub-protocols will outline the scientific rationale, eligibility, treatment schema, and other specifics for each candidate agent. The sub-protocols may define adverse events of special interest (AESIs), and can include pharmacokinetic and/or pharmacodynamic assessments that are appropriate for the specific candidate agent (pharmacodynamic assessments may require equivalent blood samples from controls).

The study consists of 2 stages:

- **Stage 1** of the study (evaluation/pilot) will evaluate the candidate agents as an add-on to the standard of care (SoC) to assess preliminary safety and efficacy. A patient will be considered to be a responder if they show an improvement of at least 2 points (from randomisation) on a 9-point category ordinal scale, are discharged from hospital, or are considered fit for discharge (a score of 0, 1, or 2 on the ordinal scale), whichever comes first, by Day 29. The time to response will be analysed on Day 29 and used to evaluate if an agent should proceed to Stage 2 of the

study. Stage 1 data will additionally be used to determine optimal study endpoints, and the number of patients to enrol into Stage 2 of the study.

- **Stage 2** of the study (confirmation) is intended to provide confirmatory data of the identified candidate agents from Stage 1, to fully evaluate disease outcomes, including severe adverse events (AEs), overall AEs, disease-related co-infection complications (eg, pneumonia, septic shock), and overall mortality in an expansion stage. Patients and outcomes from Stage 1 will not form part of Stage 2.

Some candidate agents will still be in Stage 1 of the study at the point where other candidate agents have progressed to Stage 2.

Patients will be randomised to receive one of the candidate agents that is being evaluated at the time of randomisation and whose inclusion/exclusion criteria they meet (as an add-on to SoC) or to a control arm where only SoC is administered.

Enrolment of patients will be continuous throughout the study for each candidate agent until the total randomisation number of planned patients for Stage 1 and Stage 2 is achieved. Enrolment under a sub-protocol for a specific candidate agent may also be stopped in the event of success or failure of the candidate agent. The Master Protocol will continue enrolling patients as long as there are candidate agents that are enrolling.

Number of Investigators and Study Centres:

Study centres will be located in the United Kingdom. Overall, it is estimated that approximately 18 centres and investigators will initially take part in the study.

Number of Patients:

The expected number of patients for each treatment arm is presented as part of the sample size determination below. It is estimated that up to 1800 patients will participate in the overall study.

Treatment Groups and Duration:

In each stage of the study, patients will be screened on Day -1 or Day 1, and will remain in the clinic from Day 1 until fit for discharge. Dosing with the candidate agent (as an add-on to SoC) will commence on Day 1. The last day of assessments while hospitalised will be on Day 29. An outpatient visit will be conducted on Day 60 (±4 days), with an end-of-study visit conducted on Day 90 (±6 days).

Statistical methods:

Sample size determination:

Stage 1: Based on the chosen endpoint, a preliminary analysis will be carried out when an estimated 81 events have been observed across each agent treatment and SoC or 28 days after the last patient has been randomised, whichever occurs sooner, as determined by the Independent Data and Safety Monitoring Committee (IDMC). In order to achieve this number of events, it is expected that 54 patients are needed per arm, which will provide 80% power to detect a hazard ratio of 1.6 for the occurrence of the event, when comparing each candidate agent with SoC. To allow for uncertainty in the recruitment rates, it is expected that up to 60 patients will be randomised to each arm in order to achieve the required number of events for the preliminary analysis.

Stage 2: The number of patients will be determined more precisely at the end of Stage 1, but approximately 126 patients will be randomised to each arm.

Analysis sets:

- Intention to Treat (ITT): All patients who are randomised and match the inclusion/exclusion criteria of the Master Protocol and relevant sub-protocol will be included in the ITT.
- Safety Set: All patients who are randomised and take at least 1 dose of study medication will be included in the safety set.

- Pharmacokinetic Analysis Set (PKS): All patients who are randomised and take at least 1 dose of the candidate agent and have quantifiable candidate agent concentrations postdose without protocol deviations or events affecting the pharmacokinetic results will be included in the PKS.
- Pharmacodynamic Analysis Set (PDS): All patients who are randomised and take at least 1 dose of study medication (candidate agent or SoC) and have evaluable results for at least 1 pharmacodynamic endpoint postdose. All analyses of the PDS will be based on each patient's randomised assigned treatment (not actual treatment received).

Efficacy, safety, pharmacokinetic, and pharmacodynamic results will be listed and summarised by stage, dose, and scheduled time for the respective analysis sets, where appropriate. Candidate agent concentration versus response variables may be graphically displayed for selected endpoints. Exposure-response data obtained from this study may be combined with data from other studies and used for modelling and simulations.

Data Monitoring Committee:

A Steering Committee will evaluate interim analysis data to make decisions on further progression of the candidate agents within the study, and will provide guidance, advice, and recommendations to the ACCORD program on relevant clinical issues related to the strategy, implementation, and conduct of the study.

An IDMC will objectively monitor safety data throughout the study to make recommendations to the Steering Committee regarding study conduct.

1.2 Schema

Figure 1 Study Schema

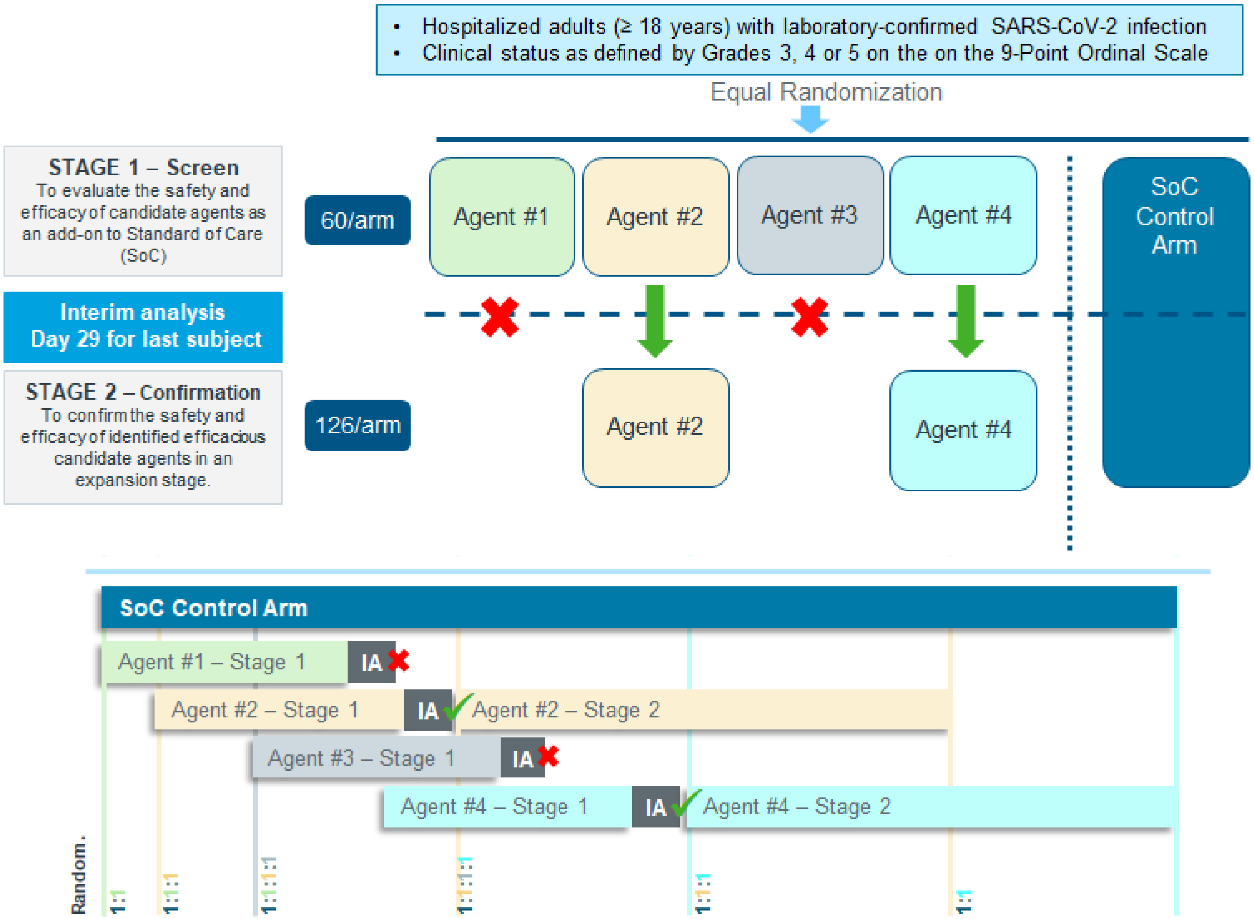

IA=interim analysis; SARS-CoV-2=severe acute respiratory syndrome coronavirus 2; SoC=standard of care.

Note: This figure shows a hypothetical situation, where in Stage 1 of the study there are 4 candidate agents being compared with the SoC, of which 2 candidate agents progress to Stage 2.

1.3 Example Schedule of Activities

	**Screening**	**Baseline**					
**Day (± Window)**	**Day -1 or Day 1**	**Day 1**	**Daily Until Hospital Discharge**	**Day 15[a] (±2 days)**	**Day 29[a] (±3 days)**	**Day 60[a] (±4 days) (Follow-up)**	**Day 90[a] (±6 days) (End of Study)**
ELIGIBILITY							
Informed consent	X						
Demographics	X						
Relevant medical history[b]	X						
Review of SARS-CoV-2 diagnostic tests	X						
Inclusion and exclusion criteria	X						
12-lead Electrocardiogram	X						
STUDY INTERVENTION							
Randomisation		X					
Administration of candidate agent		Defined in subprotocol					
Treatment with SoC		X	X				
STUDY PROCEDURES							
Clinical frailty score	X						
Diagnostic imaging (X-ray and/or computed tomography)	X						
Physical examination (including presenting symptoms, height, weight)	X						
Targeted physical examination (focused on lung auscultation)			X				
Vital signs, including temperature, pulse rate, blood pressure, respiratory rate, SpO_2		X[c]	X	X	X		

	**Screening**	**Baseline**					
**Day (± Window)**	**Day -1 or Day 1**	**Day 1**	**Daily Until Hospital Discharge**	**Day 15[a] (±2 days)**	**Day 29[a] (±3 days)**	**Day 60[a] (±4 days) (Follow-up)**	**Day 90[a] (±6 days) (End of Study)**
Clinical assessments[d]		X[c]	X	X	X		
Targeted medication review (including use of vasopressors)		X[c]	X	X	X		
Adverse event evaluation		X	X	X	X	X	X
Disease-related co-infection evaluation (including microbiologic/infectious agent assessment/results; bacteria, viral, fungi)		X	X				
Survival status		X	X	X	X	X	X
Blood gases and FiO_2 at worst PO_2[e]	X	X	X	X			
SAFETY LABORATORY							
Haematology, chemistry, liver function tests, coagulation[f]	X[g]	X[c,h]	Days 3, 5, 8, 11 (all ±1 day) while hospitalised				
Pregnancy test for females of childbearing potential	X[g]						
RESEARCH LABORATORY							
Blood (SST) for exploratory inflammatory cytokine analysis (others to be defined in sub-protocols)		X	Day 8	X	X		
Blood (sodium heparin tube) for PBMC phenotyping[i]		X	Day 8	X	X		
Blood (EDTA) for SARS-CoV-2 PCR (qualitative and quantitative)		X	Days 3, 5, 8, 11 (all ±1 day) while hospitalised	X	X		

	**Screening**	**Baseline**					
**Day (± Window)**	**Day -1 or Day 1**	**Day 1**	**Daily Until Hospital Discharge**	**Day 15[a] (±2 days)**	**Day 29[a] (±3 days)**	**Day 60[a] (±4 days) (Follow-up)**	**Day 90[a] (±6 days) (End of Study)**
Oropharyngeal/nasal swab for SARS-CoV-2 PCR (qualitative and quantitative)		X	Days 3, 5, 8, 11 (all ±1 day) while hospitalised	X	X		
Saliva for SARS-CoV-2 PCR (qualitative and quantitative)		X	Days 3, 5, 8, 11 (all ±1 day) while hospitalised	X	X		
Blood (SST) for SARS-CoV-2 serology research (host response)		X	Day 8	X	X	X	
Blood (PAXGENE) for transcriptome analysis (host genome)[j]		X	Day 8	X			
Blood (EDTA) host genome (host DNA)[j]		X					
Mid-turbinate nasal swab viral genome[j]		X					

EDTA=ethylenediaminetetraacetic acid; FiO_2=fraction of inspired oxygen; PBMC=peripheral blood mononuclear cell; PCR=polymerase chain reaction; PO_2=partial pressure of oxygen; RT PCR=reverse transcription polymerase chain reaction; SARS-CoV-2= severe acute respiratory syndrome coronavirus 2; SoC=standard of care; SpO_2=oxygen saturation; SST=serum separator tube.

Note: Additional assessments, if required, will be defined in the sub-protocol.

[a] These visits will be performed even if a patient has already been discharged. If discharged prior to scheduled visit, in-person visits are preferred, but recognising that quarantine and other factors may limit the patient's ability to return to the clinic, these visits may be conducted by telephone or with a home visit by study staff. For visits conducted by telephone, it will not be possible to perform some scheduled assessments (eg, vital signs). The Day 29 assessments will also be performed, where possible, for patients who discontinue the study prematurely.

[b] Medical history includes estimated date and time of first symptoms and number of co-morbidities (eg, respiratory, cardiovascular, metabolic, malignancy, endocrine, gastrointestinal, immunologic, renal).

[c] Baseline assessments should be performed prior to study drug administration.

[d] Includes ordinal score, National Early Warning Score 2 (NEWS2), oxygen requirement, noninvasive or invasive ventilator requirement, including start and stop of low- or high-flow oxygen supply or of any form of ventilation etc.

[e] If done as part of SoC, blood gases results to be fully recorded with date and time.

[f] For parameters, see Table 5.

[g] Laboratory tests performed in the 48 hours prior to enrolment will be accepted for determination of eligibility.
[h] Any laboratory tests performed as part of routine clinical care within the specified visit window can be used for safety laboratory testing.
[i] Samples collected for immediate laboratory processing and frozen storage.
[j] Samples collected dependent on capacity of study centre, need for reduced study burden on staff, and potentially limited access to patients.

2.0 INTRODUCTION

2.1 Study Rationale

There are currently no approved therapeutic agents available to treat coronaviruses such as severe acute respiratory syndrome coronavirus 2 (SARS-CoV-2), the causative agent of COVID-19 disease, and there is an urgent public health need for rapid development of such interventions. This adaptive platform study is designed to rapidly assess multiple candidate agents as treatments for COVID-19. Candidate drugs that are initially assessed as being efficacious will be moved from an evaluation (pilot) stage to a confirmatory stage, with candidate agents being added to and removed from the study on an ongoing basis, depending on the results of their evaluation. Patients to be included in the study will be hospitalised and may require either supplemental oxygen, noninvasive ventilation or high-flow oxygen devices.

2.2 Background

Coronaviruses are single-stranded RNA viruses, capable of causing life-threatening disease in humans and animals. The novel coronavirus SARS-CoV-2 was initially identified during an outbreak of viral pneumonia cases of unknown cause in China. Most of the initial infections outside of China were travel associated (ie, from people who had travelled from the infected regions of China to other countries), although person-to-person transmission in other countries was quickly established. The disease caused by the SARS-CoV-2 virus has been designated COVID-19.

SARS-CoV-2 binds via the angiotensin-converting enzyme (ACE) receptor located on alveolar cells and intestinal epithelia.[1] The virus is mutating, indicating that virulence and transmission will shift over time, and showing diversity in critical surface protein. New evidence suggests there are 2 groups of SARS-CoV-2; L-type and S-type.[2] S-type is the less aggressive (30%); the L-type is now the most prevalent version (70%) and is more aggressive. Additionally, individuals appear to be affected to different degree with varying symptoms and outcomes. These findings strongly support an urgent need for immediate comprehensive studies and robust validation of testing methods that combine genomic data, chart records and clinical symptoms, to help better understand the disease, enable risk assessment, triage and support public health resource planning.

Due to the rapid global widespread of SARS-CoV-2, there is an urgent need to develop efficacious treatments for the disease. Current clinical studies involve the use of already approved medications for other indications (repurposing) where it is thought that they might also be effective in the treatment of COVID-19 disease, as well as development of antibody-based therapies against the virus.

This platform study will test multiple candidate agents, with the aim of identifying potentially efficacious treatments in the shortest timeframe possible. In addition, it will support secondary research objectives that are critical for understanding the disease, spread of infection and robust tests to track it.

2.3 Benefit/Risk Assessment

There are currently no approved therapeutic agents available to treat coronaviruses such as SARS-CoV-2, and so while there may not be benefits for an individual patient participating in this study, there may be benefits to society if a safe and efficacious therapeutic agent can be identified during the global COVID-19 outbreak.

Detailed information about the known and expected risks and reasonably expected adverse events (AEs) of each candidate agent may be found in the corresponding sub-protocol for that agent.

3.0 OBJECTIVES AND ENDPOINTS

Table 1 Study Objectives and Endpoints

Objectives	Endpoints
Primary	
• Stage 1: To evaluate the efficacy of candidate agents as add-on therapies to standard of care (SoC) in patients hospitalised with COVID-19 in a screening stage.<br>• Stage 2: To confirm the efficacy of identified efficacious candidate agents in patients hospitalised with COVID-19 in an expansion stage.	• Time to clinical improvement of at least 2 points (from randomisation) on a 9-point category ordinal scale, live discharge from the hospital, or considered fit for discharge (a score of 0, 1, or 2 on the ordinal scale), whichever comes first, by Day 29 (this will also define the "responder" for the response rate analyses).<br>9-point category ordinal scale:<br>0. Uninfected, no clinical or virological evidence of infection<br>1. Ambulatory, no limitation of activities<br>2. Ambulatory, limitation of activities<br>3. Hospitalised – mild disease, no oxygen therapy<br>4. Hospitalised – mild disease, oxygen by mask or nasal prongs<br>5. Hospitalised – severe disease, noninvasive ventilation or high-flow oxygen<br>6. Hospitalised – severe disease, intubation and mechanical ventilation<br>7. Hospitalised – severe disease, ventilation and additional organ support – vasopressors, renal replacement therapy (RRT), extracorporeal membrane oxygenation (ECMO)<br>8. Death
Secondary	
• To evaluate the ability to prevent deterioration according to the ordinal scale by 1, 2, or 3 points	• The proportion of patients not deteriorating according to the ordinal scale by 1, 2, or 3 points on Days 2, 8, 15, 22, and 29.
• To evaluate the number of oxygen-free days.	• Duration (days) of oxygen use and oxygen-free days.
• To evaluate ventilator-free days and incidence and duration of any form of new ventilation use.	• Duration (days) of ventilation and ventilation-free days.<br>• Incidence of any form of new ventilation use and duration (days) of new ventilation use.
• To evaluate SARS-CoV-2 viral load.	• Qualitative and quantitative polymerase chain reaction (PCR) determination of severe acute respiratory syndrome coronavirus 2 (SARS-CoV-2) in oropharyngeal/nasal swab while hospitalised on Days 1, 3, 5, 8, 11, 15, and (optional) Day 29

• To evaluate response rate (see primary endpoint for definition of responder).	• Response rate (number and %) by treatment arm at Days 2, 8, 15, and 29.
• To evaluate time to discharge.	• Time to live discharge from the hospital.
• To evaluate overall mortality.	• Mortality at Days 15, 29, and 60.<br>• Time from treatment start date to death.
• Change in the ratio of the oxygen saturation to fraction of inspired oxygen concentration (SpO_2/FiO_2),	• SpO_2/FiO_2, measured daily from randomisation to Day 15, hospital discharge, or death
• To evaluate the safety of candidate agents as add-on therapy to SoC in patients with COVID-19.	• Physical examination.<br>• Clinical laboratory examinations.<br>• Vital signs (blood pressure/heart rate/temperature/respiratory rate).<br>• Adverse events.
• To evaluate intensive care unit (ICU) and hospitalisation length.	• Duration (days) of ICU and hospitalisation.
• To evaluate National Early Warning Score 2 (NEWS2).	• NEWS2 assessed daily while hospitalised and on Days 15 and 29.<br>• Time to a NEWS2 of ≤2, maintained for at least 24 hours.
Exploratory	
• To evaluate SARS-CoV-2 viral load.	• Qualitative and quantitative PCR determination of SARS-CoV-2 in blood and saliva (while hospitalised) on Days 1, 3, 5, 8, 11, 15, and (optional) Day 29 (may be become a secondary endpoint once the assays are available).
• To collect samples for translational research on host and viral genomics, serum antibody production, COVID-19 diagnostics, and validation of laboratory testing methods.	• Analysis of samples collected at baseline prior to treatment and at specific time points.

4.0 STUDY DESIGN

4.1 Overall Design

ACCORD-2 is a seamless, Phase 2, adaptive, randomisation platform study, designed to rapidly test candidate agents in the treatment of COVID-19 disease. The study will include hospitalised adult patients (≥18 years) who have infection with SARS-CoV-2, the virus that causes COVID-19, as confirmed by laboratory tests and/or validated point of care tests. For inclusion, patients will need to have clinical status of Grade 3 (hospitalised - mild disease, no oxygen therapy) to Grade 5 (hospitalised – severe disease, noninvasive ventilation or high-flow oxygen), as defined by a 9-point ordinal scale, which was detailed in the World Health Organization R&D Blueprint "*Novel Coronavirus - COVID-19 Therapeutic Trial Synopsis*" (February 2020). Medical history will record the estimated date and time of first symptoms.

This study will aim to identify efficacious candidate agents for treatment of COVID-19 disease. These candidate agents may include, but will not be limited to, anti-virals, human plasma-derived agents, or immunomodulatory agents. Experimental (first-in-human) agents will not be considered as candidate agents for ACCORD-2, but will instead be considered for inclusion in the separate, but linked, ACCORD-1 Phase 1/2 platform study, which will first determine the dose and assess early activity and safety signals for later consideration for inclusion into ACCORD-2. The ACCORD-2 candidate agents evaluated will include those intended as a treatment for SARS-CoV-2 infection; the study design and/or inclusion and exclusion criteria may subsequently be revised (using a protocol amendment) or a separate protocol may be initiated to include agents intended to prevent COVID-19 disease. A Steering Committee will evaluate candidate agents for progression in the study (see Section 9.7.1).

This Master Protocol outlines the overall structure of the study, including the population, inclusion and exclusion criteria, randomisation scheme, primary, secondary, and exploratory outcomes, study design, statistical methodology, and planned analyses that are common for all candidate agents to be tested. The Master Protocol is structured such that multiple candidate agents from different pharmaceutical companies can be evaluated simultaneously. The plan is to add candidate agents as they are identified, and to remove therapies once they have completed their evaluation, with the control group for a candidate agent including only patients randomised during the period in which the candidate agent group was randomised, with patients being randomised equally (ie, 1:1:1…) to the sub protocols with inclusion/exclusion criteria that they meet.

Sub-protocols will outline the scientific rationale, eligibility, treatment schema, and other specifics for each candidate agent. The sub-protocols may define adverse events of special interest (AESIs), and can include pharmacokinetic and/or pharmacodynamic assessments that are appropriate for the specific candidate agent (pharmacodynamic assessments may require

equivalent blood samples from controls). Additional patients will be recruited into the study each time a new sub-protocol (candidate agent) is added.

The study consists of 2 stages:

- **Stage 1** of the study (evaluation/pilot) will evaluate the candidate agents as an add-on to the standard of care (SoC) to assess preliminary safety and efficacy. A patient will be considered to be a responder if they show an improvement of at least 2 points (from randomisation) on a 9-point category ordinal scale, are discharged from hospital, or are considered fit for discharge (a score of 0, 1, or 2 on the ordinal scale), whichever comes first, by Day 29. The time to response will be analysed on Day 29 and used to evaluate if an agent should proceed to Stage 2 of the study. Stage 1 data will additionally be used to determine optimal study endpoints, and the number of patients to enrol into Stage 2 of the study.
- **Stage 2** of the study (confirmation) is intended to provide confirmatory data of the identified candidate agents from Stage 1, to fully evaluate disease outcomes, including severe AEs, overall AEs, disease-related co-infection complications (eg, pneumonia, septic shock), and overall mortality in an expansion stage. Patients and outcomes from Stage 1 will not form part of Stage 2.

Some candidate agents will still be in Stage 1 of the study at the point where other candidate agents have progressed to Stage 2.

Patients will be randomised to receive one of the candidate agents that is being evaluated at the time of randomisation and whose inclusion/exclusion criteria they meet (as an add-on to SoC) or to a control arm where only SoC is administered.

In each stage of the study, patients will be screened on Day -1 or Day 1, and will remain in the clinic from Day 1 until fit for discharge. Dosing with the candidate agent (as an add-on to SoC) will commence on Day 1. The last day of assessments while hospitalised will be on Day 29. An outpatient visit will be conducted on Day 60 (±4 days), with an end-of-study visit conducted on Day 90 (±6 days).

Enrolment of patients will be continuous throughout the study for each candidate agent until the total randomisation number of planned patients for Stage 1 and Stage 2 is achieved. Enrolment under a sub-protocol for a specific candidate agent may also be stopped in the event of success or failure of the candidate agent. The Master Protocol will continue enrolling patients as long as there are candidate agents that are enrolling. It is estimated that up to 1800 patients will participate in the overall study.

Study centres will be located in the United Kingdom. Overall, it is estimated that approximately 18 centres and investigators will initially take part in the study.

4.2 Scientific Rationale for Study Design

There are currently no approved therapeutic agents available to treat coronaviruses such SARS-CoV-2, the causative agent of as COVID-19 disease, and there is an urgent public health need for rapid development of such interventions. This adaptive platform study is designed to rapidly assess multiple candidate agents as treatments for COVID-19. Candidate drugs that are initially assessed as being efficacious will be moved from an evaluation (pilot) stage to a confirmatory stage, with candidate agents being added to and removed from the study on an ongoing basis, depending on the results of their evaluation. Patients to be included in the study will be hospitalised and may require either supplemental oxygen, noninvasive ventilation or high-flow oxygen devices.

This study utilises an adaptive design that maximises efficiency in identifying a safe and efficacious therapeutic agent for COVID-19. Some candidate agents may be in limited supply and this study design enables continuation of the study even if an agent becomes unavailable. In addition, the adaptive design allows for the evaluation of new candidate agents as they are identified.

4.3 Justification for Dose

Justification for the dose of each candidate agent will be included in the corresponding sub-protocol.

4.4 End of Study Definition

For each sub-protocol, the end of the study for that candidate agent will be defined as the date on which the last patient completes the last visit for that sub-protocol.

For the overall study, the end of the study will be defined as the date on which the last patient completes the last visit for the final sub-protocol to be concluded.

Once a patient has completed this study, there are no restrictions on them entering another study, subject to the eligibility criteria of that subsequent study.

5.0 STUDY POPULATION

Prospective approval of protocol deviations to recruitment and enrolment criteria, also known as protocol waivers or exemptions, is not permitted.

The inclusion and exclusion criteria listed below may be supplemented by additional criteria stipulated in the sub-protocols that are specific to the target candidate being tested (eg, criteria related to prohibited medications). In order to enrol, a patient or legally authorised representative must sign an informed consent form (ICF) and meet all entry criteria for both the Master Protocol and at least 1 respective sub-protocol.

5.1 Inclusion Criteria

Patients are eligible to be included in the study only if all of the following criteria apply (as well as all criteria from the appropriate sub-protocol):

1. Adults (≥18 years) with SARS-CoV-2 infection confirmed by laboratory tests and/or point of care tests.
2. A score of Grade 3 to 5 on the 9-point ordinal scale.
3. The patient, and their partner(s), agree to use medically-accepted double-barrier methods of contraception (eg, barrier methods, including male condom, female condom or diaphragm with spermicidal gel) during the study and for at least 6 weeks after termination of study therapy. A vasectomised partner would be considered an appropriate birth control method provided that the partner is the sole male sexual partner and the absence of sperm has been confirmed. If not, an additional method of contraception should be used.
   or
   The patient is a woman who is not of childbearing potential (as defined in Appendix 5)
4. Women who are lactating who agree not to breastfeed their child during the study and for at least 6 weeks after termination of study therapy (they may continue to express milk away from the child during this period, but this milk must be discarded).
5. Ability to provide informed consent signed by the study patient or legally authorised representative.

5.2 Exclusion Criteria

Patients are excluded from the study if any of the following criteria apply (or any of the criteria from the appropriate sub-protocol):

1. Patients who have previously had a score of 6 or 7 on the 9-point ordinal scale.
2. Any patient whose interests are not best served by study participation, as determined by a senior attending clinician.
3. Alanine aminotransferase (ALT)/aspartate aminotransferase (AST) >5 × the upper limit of normal (ULN).

4. Known active infection with HIV or hepatitis B or C.
5. Stage 4 severe chronic kidney disease or requiring dialysis (ie, estimated glomerular filtration rate <30 mL/min/1.73 m^2).
6. History of the following cardiac conditions:
   a) Myocardial infarction within 3 months prior to the first dose
   b) Unstable angina
   c) History of clinically significant dysrhythmias (long QT features on electrocardiogram [ECG], sustained bradycardia [≤55 bpm]), left bundle branch block, cardiac pacemaker or ventricular arrhythmia) or history of familial long QT
7. Screening 12-lead ECG with a measurable QTc interval according to Fridericia correction (QTcF) >500 ms.
8. Anticipated transfer to another hospital that is not a study centre within 72 hours.
9. Allergy to any study medication.
10. Experimental off-label usage of medicinal products as treatments for COVID-19.
11. Patients participating in another clinical study of an investigational medicinal product.
12. Active tuberculosis defined as requiring current treatment for tuberculosis.

5.3 Lifestyle Considerations

Any lifestyle considerations that are specific to the candidate agent will be defined in the corresponding sub-protocol.

Female patients are advised to avoid becoming pregnant during the study.

5.4 Screen Failures

Screen failures are defined as patients who consent to participate in the clinical study but are not subsequently randomly assigned to study treatment. A minimal set of screen failure information is required to ensure transparent reporting of screen failure patients to meet the Consolidated Standards of Reporting Trials (CONSORT) publishing requirements and to respond to queries from regulatory authorities. Minimal information includes demography, screen failure details, eligibility criteria, and any serious adverse event (SAE).

6.0 STUDY TREATMENT

Study treatment is defined as any investigational treatment(s), marketed product(s), placebo, or medical device(s) intended to be administered to a patient according to the study protocol.

6.1 Study Treatment(s) Administered

This platform study will include multiple treatments (candidate agents) from different pharmaceutical companies. Each candidate agent included within the study will have a sub-protocol that will provide details of that treatment, including route and mode of administration, dose, dosage regimen, and duration of treatment.

These candidate agents may include, but will not be limited to, anti-virals, human plasma-derived agents, or immunomodulatory agents; experimental (first-in-human) agents will not be considered as candidate agents.

The SoC will be based on appropriate guidelines in place at the time of treatment on the study, eg, the current National Institute for Health and Care Excellence 'COVID-19 rapid guideline: critical care in adults'. The SoC may change during the course of the study as new information becomes available about treating COVID-19.

6.2 Preparation/Handling/Storage/Accountability

Candidate agents will be shipped to the study centre either directly from participating companies, from the Sponsor, or from other regional or local drug repositories. All other supplies will be provided by the study centre.

The Investigator or designee must confirm appropriate temperature conditions have been maintained during transit for all candidate agents received and any discrepancies are reported and resolved before use of the candidate agent.

Only patients enrolled in the study may receive a candidate agent and only authorised study centre staff may supply or administer candidate agents. All candidate agents must be stored in a secure, environmentally controlled, and monitored (manual or automated) area in accordance with the labelled storage conditions with access limited to the Investigator and authorised study centre staff.

The Investigator, institution, or the head of the medical institution (where applicable) is responsible for candidate agent accountability, reconciliation, and record maintenance (ie, receipt, reconciliation, and final disposition records).

Further guidance and information for the final disposition of unused candidate agents are provided in the Pharmacy Manual for the candidate agent.

The Investigator, a member of the study centre staff, or a hospital pharmacist must maintain an adequate record of the receipt and distribution of all candidate agents using the Drug Accountability Form. These forms must be available for inspection at any time.

6.3 Measures to Minimise Bias: Randomisation and Blinding

No blinding will be used in this study.

The study will randomise participants on an ongoing basis to receive either one of the active candidate treatment arms as an add-on to SoC or to receive SoC alone. Randomisation will be stratified by study centre and baseline severity grade.

If additional arms (candidate treatments) are added to or dropped from the study, randomisation will proceed with an equal probability of assignment to each of the remaining arms, except for situations where a patient does not meet the inclusion/criteria for, and hence would be excluded from, one or more of the sub-protocols.

6.4 Study Treatment Compliance

For each candidate agent, the prescribed dosage, timing, and mode of administration may not be changed, except as defined in Section 6.6. Any departures from the intended regimen must be recorded in the electronic case report forms (eCRFs).

Each dose of candidate agent will be administered by a member of the clinical research team that is qualified and licensed to administer the study product. Administration and date, time, and route will be entered into the eCRF.

6.5 Concomitant Therapy

Any medication or vaccine (including over-the-counter or prescription medicines, vitamins, and/or herbal supplements) that the patient is receiving at the time of enrolment (including screening) or receives during the study must be recorded in the eCRF along with:

- Reason for use
- Dates of administration including start and end dates
- Dosage information including dose and frequency

The Medical Monitor should be contacted if there are any questions regarding concomitant or prior therapy.

A list of excluded medications/therapy will be provided for each candidate agent in the corresponding sub-protocol.

6.5.1 Rescue Medicine

Any permitted rescue medications appropriate for the candidate agent administered will be detailed in the corresponding sub-protocol.

6.6 Dose Modification

An Independent Data and Safety Monitoring Committee (IDMC) will actively monitor interim data throughout the duration of the study to make recommendations about early study closure or changes to the study treatment arms.

Details of permitted dose modifications for specific candidate agents will be detailed in the corresponding sub-protocol.

6.7 Treatment after the End of the Study

This is a study in an acute severe respiratory disease and study treatment will not be continued/required following hospital discharge.

7.0 DISCONTINUATION OF STUDY TREATMENT AND PATIENT DISCONTINUATION/WITHDRAWAL

7.1 Discontinuation of Study Treatment

A patient in this study may discontinue their assigned candidate agent for any of the following reasons:

- Patient requests to discontinue study drug.
- Occurrence of any medical condition or circumstance that does not allow the patient to adhere to the requirements of the protocol or patient fails to comply with protocol requirements or study-related procedures.
- Any SAE, clinically significant AE, severe laboratory abnormality (including abnormal liver function test results), intercurrent illness, or other medical condition that indicates to the Investigator that continued participation is not in the best interest of the patient.
- Pregnancy recognised post start and prior to end of study treatment.

Unless the patient withdraws consent, those who discontinue study drug early should remain in the study for further acquisition of endpoint measurements. The reason for patient discontinuation of study drug should be documented in the eCRF.

7.2 Patient Discontinuation/Withdrawal from the Study

- A patient may withdraw from study treatment or the study at any time at his/her own request, or may be withdrawn from study treatment or the study at any time at the discretion of the Investigator for safety, behavioural, compliance, or administrative reasons.
- If the patient withdraws consent for disclosure of future information, the Sponsor may retain and continue to use any data collected before such a withdrawal of consent.
- If a patient withdraws from the study, he/she may request destruction of any residual remaining samples taken and not tested, and the Investigator must document this in the study centre study records. However, any laboratory or test data generated from samples that have already been processed and included in secondary translational research may not be recalled.
- See Schedule of Activities (SoA, Section 1.3) for data to be collected at the time of study discontinuation and follow-up and for any further evaluations that need to be completed.

7.3 Lost to Follow-up

A patient will be considered lost to follow-up if he or she repeatedly fails to return for scheduled visits and is unable to be contacted by the study centre.

The following actions must be taken if a patient fails to return to the study centre for a required study visit:

- All efforts should be made to ascertain the vital status of the patient.

- The study centre must attempt to contact the patient and reschedule the missed visit as soon as possible and counsel the patient on the importance of maintaining the assigned visit schedule and ascertain whether or not the patient wishes to and/or should continue in the study.
- Before a patient is deemed lost to follow-up, the Investigator or designee must make every effort to regain contact with the patient (where possible, 3 telephone calls and, if necessary, a certified letter to the patient’s last known mailing address or local equivalent methods). These contact attempts should be documented in the patient’s medical record.
- Should the patient continue to be unreachable, he/she will be considered to have withdrawn from the study.

8.0 STUDY ASSESSMENTS AND PROCEDURES

Note: Given the nature of the disease and the condition of the patients, it may not always be possible to perform all planned assessments at all time points; however, all efforts should be made to perform assessments as long as it considered clinically safe to do so.

An example SoA is presented in Section 1.3. For the ‘SoC only’ arm, this will be the schedule that is followed. For each candidate agent, the specific SoA for that treatment arm will be detailed in the corresponding sub-protocol.

Protocol waivers or exemptions are not allowed.

Immediate safety concerns should be discussed with the Sponsor immediately upon occurrence or awareness to determine if the patient should continue or discontinue study treatment.

Adherence to the study design requirements, including those specified in the corresponding sub-protocol for the candidate agent, is essential and required for study conduct.

All screening evaluations must be completed and reviewed to confirm that potential patients meet all eligibility criteria. The Investigator will maintain a screening log to record details of all patients screened and to confirm eligibility or record reasons for screening failure, as applicable.

Procedures conducted as part of the patient’s routine clinical management (eg, blood count) and obtained before signing of the ICF may be utilised for screening or baseline purposes provided the procedures met the protocol-specified criteria and were performed within the timeframe defined in the SoA.

The maximum amount of blood collected from each patient over the duration of the study, including any extra assessments that may be required, will not exceed 500 mL. Repeat or unscheduled samples may be taken for safety reasons or for technical issues with the samples.

All biological samples collected will be stored in a secure storage space in line with recommended guidelines for infectious substance handling, and with adequate measures to protect confidentiality. The samples will be retained while research on COVID-19 continues but no longer than 10 years or other period as per local requirements.

8.1 Efficacy Assessments

8.1.1 Improvement on the 9-Point Scale

For the purposes of this study, the condition of each potential participant in the study will be assessed using a 9-point category ordinal scale:

0. Uninfected, no clinical or virological evidence of infection
1. Ambulatory, no limitation of activities
2. Ambulatory, limitation of activities
3. Hospitalised – mild disease, no oxygen therapy
4. Hospitalised – mild disease, oxygen by mask or nasal prongs
5. Hospitalised – severe disease, noninvasive ventilation or high-flow oxygen
6. Hospitalised – severe disease, intubation and mechanical ventilation
7. Hospitalised – severe disease, ventilation and additional organ support – vasopressors, renal replacement therapy (RRT), extracorporeal membrane oxygenation (ECMO)
8. Death

For Grade 5, 'high-flow oxygen' consists of a heated, humidified high-flow nasal cannula delivering up to 100% heated and humidified oxygen at a maximum flow of 60 L/minute via nasal prongs or cannula to a patient at rates of flow higher than traditional low-flow therapy. In high-flow oxygen, the fraction of inspired oxygen (FiO_2) can be titrated from 21% to 100% independent of the flow rate.

Note: If initial data suggest that there are too few patients in certain categories, the decision may be made to combine parts of the ordinal scale, leading to a smaller number of categories.

To be considered for inclusion in the study, patients must be Grade 3 to 5 on this scale.

To be considered a "responder" to treatment with a target candidate, a patient needs to show an improvement of at least 2 points (from randomisation) on this scale. For example, a patient who is Grade 5 (hospitalised - severe disease, noninvasive ventilation or high-flow oxygen) at randomisation but improves to Grade 3 (hospitalised - mild disease, no oxygen therapy) would be considered to be a responder. A patient with a live discharge from hospital or who is considered fit for discharge (a score of 0, 1, or 2 on the ordinal scale) will also be considered to be a responder.

The primary endpoint will be time to clinical improvement of 2 points (from randomisation). The response rate (number and percentage of patients) will be determined for each treatment arm (target candidate) on Days 2, 8, 15, 22, and 29 as a secondary endpoint.

In addition, a secondary endpoint will be proportion of patients not deteriorating according to the ordinal scale by 1, 2, or 3 points on Days 2, 8, 15, 22, and 29.

8.1.2 Other Efficacy Assessments

Overall mortality will be assessed on Days 15, 29, and 60, and, where applicable, time from treatment (candidate agent) start date to death will be calculated.

The duration (days) of oxygen use and oxygen-free days (to Day 29) will be a secondary endpoint.

The duration of mechanical ventilator-free days (to Day 29) will be a secondary endpoint, as will be the incidence and duration (days) of new mechanical ventilation use.

The duration (days) of intensive care unit (ICU) and hospitalisation will also be a secondary endpoint.

The National Early Warning Score 2 (NEWS2) has demonstrated an ability to discriminate patients at risk of poor outcomes.[3] This score is based on 6 physiological measurements (respiration rate, oxygen saturation [SpO_2], systolic blood pressure, pulse rate, level of consciousness or new confusion, and temperature), and the overall score is uplifted by 2 points for patients requiring supplemental oxygen to maintain their recommended SpO_2. For patients confirmed to have hypercapnic respiratory failure on blood gas analysis, on either a prior or their current hospital admission, and requiring supplemental oxygen, the following are recommended: (i) a prescribed SpO_2 target range of 88% to 92%, and (ii) that the dedicated SpO_2 scoring scale (Scale 2) on the NEWS2 chart should be used to record and score the SpO_2 for the NEWS2. NEWS2 will be assessed daily while hospitalised and on Days 15 and 29. This should be evaluated at the first assessment of a given study day.

8.2 Safety Assessments

Planned time points for all safety assessments are provided in the SoA (Section 1.3).

8.2.1 Physical Examinations

A general physical examination will be performed at screening, including assessment of presenting symptoms.

At subsequent assessments, a symptom-directed (targeted) physical examination will be performed, focused on lung auscultation.

8.2.2 Vital Signs and Blood Gases

Temperature, pulse rate, blood pressure, and respiratory rate will be assessed. Blood pressure and pulse measurements will be assessed with a completely automated device. SpO_2 will also be assessed. Manual techniques will be used only if an automated device is not available.

Blood gases (oxygen and carbon dioxide) and respiratory support will be recorded. FiO_2, the assumed percentage of oxygen concentration participating in gas exchange in the alveoli, will also be recorded.

Measurements will be taken in line with standard practices for the study centre.

Vital signs measurements will contribute to the NEWS2 score (see Section 8.1.2).

8.2.3 Clinical Safety Laboratory Assessments

Fasting is not required before collection of laboratory samples. See Appendix 3 for the list of clinical laboratory tests to be performed and to the SoA (Section 1.3) for the timing and frequency. Additional assessments, if required, will be defined in the sub-protocol.

8.3 Virologic Load

Qualitative and/or quantitative polymerase chain reaction (PCR) determination of SARS-CoV-2 in oropharyngeal/nasal swab, in saliva, and in blood (while hospitalised) on Days 1, 3, 5, 8, 11, 15, and (optional) Day 29 will be performed. Additional assessments, if required, will be defined in the sub-protocol.

8.4 Adverse Events

The definitions of an AE or SAE can be found in Appendix 4.

AEs will be reported by the patient (or, when appropriate, by a caregiver, surrogate, or the participant's legally authorised representative).

The Investigator and any designees are responsible for detecting, documenting, and recording events that meet the definition of an AE or SAE and remain responsible for following up AEs that are serious, considered related to the study treatment or study procedures, or that caused the patient to discontinue the study treatment or the study (see Section 7.0).

8.4.1 Time Period and Frequency for Collecting AE and SAE Information

All AEs will be collected from provision of informed consent until the final follow-up visit, at the time points specified in the SoA (Section 1.3).

All SAEs will be recorded and reported to the Sponsor or designee within 24 hours, as indicated in Appendix 4. The Investigator will submit any updated SAE data to the Sponsor within 24 hours of it being available.

Investigators are not obligated to actively seek AEs or SAEs after conclusion of the study participation. However, if the Investigator learns of any SAE, including a death, at any time after a patient has been discharged from the study, and he/she considers the event to be reasonably

related to the study treatment or study participation, the Investigator must promptly notify the Sponsor.

The method of recording, evaluating, and assessing causality of AEs and SAEs and the procedures for completing and transmitting SAE reports are provided in Appendix 4.

8.4.2 Method of Detecting AEs and SAEs

Care will be taken not to introduce bias when detecting AEs and/or SAEs. Open-ended and non-leading verbal questioning of the patient is the preferred method to inquire about AE occurrences.

8.4.3 Follow-up of AEs and SAEs

After the initial AE/SAE report, the Investigator is required to proactively follow each patient at subsequent visits/contacts. All SAEs, and nonserious AESIs (see Section 8.4.6), will be followed until resolution, stabilisation, the event is otherwise explained, or the patient is lost to follow-up (as defined in Section 7.3). Further information on follow-up procedures is given in Appendix 4.

8.4.4 Regulatory Reporting Requirements for SAEs

Prompt notification by the Investigator to the Sponsor of an SAE (within 24 hours of becoming aware) is essential so that legal obligations and ethical responsibilities towards the safety of patients and the safety of a study treatment under clinical investigation are met.

The Sponsor has a legal responsibility to notify both the local regulatory authority and other regulatory agencies about the safety of a study treatment under clinical investigation. The Sponsor will comply with country-specific regulatory requirements relating to safety reporting to the regulatory authority, main research ethics committee (REC), and Investigators.

Investigator safety reports must be prepared for suspected unexpected serious adverse reactions (SUSAR) according to local regulatory requirements and Sponsor policy and forwarded to Investigators as necessary.

An Investigator who receives an Investigator safety report describing an SAE or other specific safety information (eg, summary or listing of SAEs) from the Sponsor will review and then file it along with the Investigator's Brochure and will notify the REC, if appropriate according to local requirements.

The Sponsor will inform the candidate agent owner of any important safety findings related to the candidate agent that arise during the conduct of this study. Similarly, the candidate agent owner will inform the Sponsor of any important safety findings related to the candidate agent that they become aware of from other studies.

8.4.5 Pregnancy

- Details of all pregnancies in female patients will be collected after the start of study treatment and until a time period that is at least 5 terminal half-lives after the last dose of the candidate agent.
- If a pregnancy is reported, the Investigator should inform the Sponsor within 24 hours of learning of the pregnancy and should follow the procedures outlined in Appendix 5.
- Abnormal pregnancy outcomes (eg, spontaneous abortion, foetal death, stillbirth, congenital anomalies, ectopic pregnancy) are considered SAEs.

8.4.6 Adverse Events of Special Interest

AESIs will be specific to the target candidate and, as such, will be defined in the corresponding sub-protocol.

8.4.7 Disease-Related Events and/or Disease-Related Outcomes Not Qualifying as Adverse Events or Serious Adverse Events

Events that are typically associated with the disease under study will not be reported according to the standard process for expedited reporting of SAEs even though the event may meet the definition of an SAE.

8.5 Treatment of Overdose

In the event of an overdose of a candidate agent, the Investigator/treating physician should:

1. Contact the Medical Monitor immediately.
2. Closely monitor the patient for any AEs/SAEs and laboratory abnormalities until the candidate agent can no longer be detected systemically.
3. Obtain a plasma sample for pharmacokinetic (PK) analysis if requested by the Medical Monitor (determined on a case-by-case basis).
4. Document the quantity of the excess dose as well as the duration of the overdose in the eCRF.

Decisions regarding dose interruptions or modifications will be made by the Investigator in consultation with the Medical Monitor based on the clinical evaluation of the patient.

8.6 Pharmacokinetics

Any PK assessments performed will be specific to the candidate agent, and will be discussed in the corresponding sub-protocol, including a schedule for collection of samples of blood or other biological samples for analysis.

If a patient refuses blood collection for PK analysis, this will not be considered a protocol violation as the PK analysis is not a primary or secondary objective of the protocol.

8.7 Pharmacodynamics

Blood samples will be collected for analysis of inflammatory cytokines (eg, interleukins). Further details regarding the specific cytokines to be analysed and the analytical methods will be presented in the laboratory manual.

Any other pharmacodynamic assessments performed will be specific to the candidate agent and will be discussed in the corresponding sub-protocol, including a schedule for collection of samples of blood or other biological samples for analysis. In some sub-protocols, this will require matched sampling of control patients.

If a patient refuses blood collection for pharmacodynamic analysis, this will not be considered a protocol violation as the pharmacodynamic analysis is not a primary or secondary objective of the protocol.

8.8 Immunology

At time points indicated in the SoA (Section 1.3), a blood sample will be collected in a sodium heparin tube for immediate local laboratory processing and frozen storage. Samples will be analysed for peripheral blood mononuclear cell (PBMC) phenotyping.

8.9 Genomics

Prior to randomisation to a treatment arm, the following samples may be collected from patients at baseline (Day 1) and may be collected at the end of treatment (except for whole genome sequencing) for optional genetic analyses:

- A whole blood sample in a PAXgene tube at baseline (Day 1), on Day 8, and at the end of treatment: this sample will be used for transcriptomics, used to study an individual's transcriptome.
- A whole blood sample in an ethylenediaminetetraacetic acid (EDTA) tube at baseline (Day 1): this sample will primarily be used to provide DNA for whole genome sequencing.
- Oropharyngeal/nasal swab at baseline (Day 1): this sample will be used for viral sequencing.

Residual samples postanalysis will be biobanked for potential future research. An independent Steering Committee will evaluate and prioritise use of samples in future translational research studies.

In the event of DNA or RNA extraction failure, a replacement blood sample for genomics may be requested from the patient. Signed informed consent will be required to obtain a replacement sample unless it was included in the original consent.

See Appendix 6 for information regarding host DNA extraction and genetic research.

Separate informed consent is not required for collection of the host genome research samples, but specific points relating to genomics will be included in the study consent documents.

Host blood samples taken for genomics will be collected for processing into DNA within a short timeframe.

In cases of deferred consent, DNA samples collected from patients who subsequently die and from whom qualifying consent has not be obtained for genomic analysis, may be analysed after their death if the data are completely anonymised, the analysis is conducted in accordance with the approved and authorised clinical trial objectives, and is used for purposes of public interest and public health in accordance with local legislation.

Blood samples will not be taken from deceased individuals.

8.10 Serology Research (Host Response)

At time points indicated in the SoA (Section 1.3), a whole blood sample will be collected in a serum separator tube to be processed to serum (within 48 hours) and aliquoted by the central laboratory into 5 serum samples to be used in different antibody analysis methods for understanding immunity, antibody repertoire, and for validation of appropriate testing methods.

9.0 STATISTICAL CONSIDERATIONS

9.1 Design Overview

This is a 2-stage, multi-treatment regimen clinical study. Experimental treatment regimens may enter the study at any time and are in their stage at entering.

In Stage 1 (evaluation/pilot), the goal is to decide whether the treatment regimen is sufficiently promising to allow the candidate agents to go forward for continued evaluation in confirmatory Stage 2.

The maximum sample size for a candidate agent in Stage 1 is 60 patients. For those treatments that graduate within Stage 1, further efficacy evaluation will be carried out in Stage 2 using a second set of patients.

9.2 Statistical Hypotheses

The primary hypothesis at Stage 1 is time to response (2-point improvement in the 9-point ordinal scale, live discharge from hospital, or considered fit for discharge, as analysed at Day 29) of the candidate arm is shorter than the SoC.

The primary hypothesis at Stage 2 is time to response (as analysed at Day 29) of the candidate arm is shorter than the SoC.

As the events are 2-point improvement in 9-point ordinal scale, live discharge, or considered fit for discharge, a hazard ratio (HR) larger than 1 is favourable to the test treatment, with the HR comparing the test treatment with the control.

$$H_0: HR \leq 1 \; vs \; H_A: HR > 1$$

No multiplicity adjustment will be made for the fact that each candidate arm will be compared with the control arm (SoC). In Stage 2, each comparison will be tested at the Type I error rate=0.05 (1-sided alpha=0.025) using a log-rank test; HR will be estimated based on the Cox regression model.

9.3 Sample Size Determination

The study consists of two parts: Stage 1 (pilot stage) and Stage 2 (confirmatory stage).

Stage 1 (Pilot Stage)

This stage will focus on assessing a number of candidate agents with SoC, with the aim of carrying forward a subset of candidate agents into Stage 2, the confirmatory stage. Approximately 60 patients will be randomised into each arm at this stage of the study, whereby patients will receive either one of the candidate agents (with SoC) or SoC alone. The chosen endpoint to compare treatments will be the time to a 2-point improvement on a 9-point category ordinal scale, discharge from hospital, or considered fit for discharge.

Based on the chosen endpoint, a preliminary analysis will be carried out when an estimated 81 events have been observed across each agent treatment and SoC or 28 days after the last patient has been randomised, whichever occurs sooner, as determined by the IDMC. In order to achieve this number of events, it is expected that 54 patients are needed per arm, which will provide 80% power to detect a hazard ratio of 1.6 for the occurrence of the event, when comparing each candidate agent with SoC. This calculation is based on a 1-sided test and a 10% significance level, and assumes 85.5% of patients will improve, be discharged from hospital, or considered fit for discharge at Day 29 for a candidate treatment versus 70% of patients for the SoC (the estimate of SoC was taken from Cao 2020[4]). To allow for uncertainty in the recruitment rates, it is expected that up to 60 patients (rather than 54 patients) will be randomised to each arm in order to achieve the required number of events for the preliminary analysis.

Safety data will also be assessed.

Full details will be provided in the IDMC charter.

Stage 2 (Confirmatory Stage)

This stage will focus on assessing potential candidate agents carried forward from Stage 1 with SoC. Further patients will be recruited into this stage of the study, with approximately

126 additional patients randomised to each arm. The set of patients in Stage 1 will not be included in the set of patients for Stage 2.

As in Stage 1, the time to a 2-point improvement on a 9-point category ordinal scale, discharge from hospital, or considered fit for discharge will be used to assess each candidate agent with SoC. Based on this endpoint, 126 patients will be needed per arm to observe an estimated 188 events across each candidate agent and SoC. This number of patients and events will provide 90% power to detect a hazard ratio of 1.6 for the occurrence of the event, when comparing each candidate agent with SoC. This calculation is based on a 1-sided test and a 2.5% significance level, and assumes the same response rates for the candidate agents and SoC as quoted for Stage 1.

<u>Stage 1 Leading into Stage 2</u>

The analyses carried out at Stage 1, the pilot stage, will incorporate an assessment of which candidate agents to progress to Stage 2, the confirmatory stage. At the same time, the results observed at Stage 1 will facilitate decision making for Stage 2, allowing the selection of the primary analysis and endpoint, and the re-evaluation of sample size. If the proposed sample size for Stage 2 goes beyond 150 patients per arm based on the data observed at pilot stage, this must be ratified by the steering committee.

<u>Sample Size Calculations</u>

The calculations below (Table 2) are associated with time to improvement, discharge from hospital, or fit for discharge, and are associated with 80% and 90% power. The sample sizes are derived from a log-rank test, using a hazard ratio of 1.6 for the occurrence of the event, when comparing a candidate agent with SoC.

Table 2 Sample Size for 80% and 90% Power for Time to Improvement, Discharge from Hospital, or Fit for Discharge Using Log-rank Test for a Hazard Ratio of 1.6 in Treatment Arm to Standard of Care

**2-Point Improvement, Discharge from Hospital, or Fit for Discharge at Day 29**				**Patients/Arm [Total Events]**		
SoC	Experimental arm	Relative improvement to SoC	Hazard ratio	One-sided alpha	Power	
					80%	90%
0.7	0.855	15.5%	1.6	0.1	54 [81]	79 [118]
	0.9	20%	1.9	0.1	29 [43]	42 [63]
0.7	0.855	15.5%	1.6	0.025	94 [141]	126 [188]
	0.9	20%	1.9	0.025	50 [75]	67 [100]

SoC=standard of care

9.4 Populations for Analyses

For purposes of analysis, the analysis sets in Table 3 are defined.

Table 3 Analysis Sets

Analysis Set	Description
Intention to Treat (ITT)	All patients who are randomised and match the inclusion/exclusion criteria of the Master Protocol and relevant sub protocol.
Safety Set	All patients who are randomised and take at least 1 dose of study medication.
Pharmacokinetic Analysis Set (PKS)	All patients who are randomised and take at least 1 dose of the candidate agent and have quantifiable candidate agent concentrations postdose without protocol deviations or events affecting the pharmacokinetic results.
Pharmacodynamic Analysis Set (PDS)	All patients who are randomised and take at least 1 dose of study medication (candidate agent or standard of care) and have evaluable results for at least 1 pharmacodynamic endpoint postdose. All analyses of the PDS will be based on each patient's randomised assigned treatment (not actual treatment received).

9.5 Statistical Analyses

The Statistical Analysis Plan (SAP) will describe the patient analysis sets to be included in the analyses and procedures for accounting for missing, unused, and spurious data. Addenda to the SAP will be produced for each sub-protocol.

Efficacy, safety, PK, and pharmacodynamic results will be listed and summarised by stage (pilot stage; confirmatory stage), dose, and scheduled time for the respective analysis sets, where appropriate. Candidate agent concentration versus response variables may be graphically displayed for selected endpoints. Exposure-response data obtained from this study may be combined with data from other studies and used for modelling and simulations.

9.5.1 Efficacy Analyses

Table 4 Efficacy Analyses

Endpoint	Statistical Analysis Methods
Primary	Stage 1:<br>Time to 2-point improvement on the 9-point ordinal scale, live discharge, or fit for discharge (as analysed at Day 29), whichever comes first, will be compared with each treatment arm with standard of care (SoC) using log-rank test at the one-sided 0.1 alpha level. Patients in the Stage 1 Intention to Treat (ITT) set will be used.<br>Stage 2:<br>The time to 2-point improvement on the 9-point ordinal scale, live discharge, or fit for discharge (as analysed at Day 29), whichever comes first, analyses will be conducted between the selected treatments plus SoC and SoC alone. The primary endpoint could be re-determined based on Stage 1 results. Patients in the Stage 2 ITT set will be used for the following analyses:<br>The time to 2-point improvement on 9-point ordinal scale, live discharge, or fit for discharge at Day 29 will be conducted between each selected treatment group and control using the log-rank statistic, stratifying for study centre and baseline severity grade, and the p-value associated with the log-rank statistic will be compared at the 2-sided 0.05 alpha level. The hazard ratio between treatment arm and SoC will be estimated using Cox regression, including treatment, age category (age <70 years vs ≥ 70 years), baseline severity, age category and treatment interaction, baseline severity and treatment interaction as fixed effect, and study centre as random effect.
Secondary	Stage 1:<br>2-point improvement on a 9-point ordinal scale will be analysed using CMH test, with stratification factors as covariates.<br>Time to live discharge.<br>Stage 2:<br>Binary endpoints, including response rates at Days 2, 8, 15, 22, and 29, and mortality rates at Days 15, 29, and 60, will be analysed using the same CMH test and logistic regression model as in the primary endpoint.<br>Time-to-event endpoints, including time to clinical improvement, time to death, and time to live discharge, from treatment start date will be plotted with Kaplan-Meier curves and will be tested with the log-rank test.<br>Other continuous endpoints will be summarised with descriptive statistics such as mean, standard deviation, median, minimum, and maximum.
Exploratory	Will be described in the Statistical Analysis Plan finalised before database lock

9.5.2 Safety Analyses

Analysis of adverse events

All AEs reported in this study will be coded using the currently available version of the Medical Dictionary for Regulatory Activities (MedDRA). Coding will be to lowest level terms. The preferred term (PT), and the primary system organ class will be listed.

Summaries of all treatment-emergent adverse events (TEAEs) by treatment group will include:

- The number (n) and percentage (%) of patients with at least 1 TEAE by system organ class and PT
- TEAEs by severity, presented by system organ class and PT
- TEAEs by relationship to treatment (related, not related), presented by system organ class and PT
- Treatment-emergent AESIs (defined with a PT or a prespecified grouping)

Deaths and other SAEs will be listed and summarised by treatment group.

TEAEs leading to permanent treatment discontinuation will be listed and summarised by treatment group.

Analysis of clinical laboratory evaluations

The number and proportion of patients with normal/abnormal laboratory tests or different grades on Days 3, 5, 8, and 11 (or day of discharge) will be presented as shift tables by the baseline status (Day 1).

Analysis of vital signs

Potentially clinically significant abnormality (PCSA) values are defined as abnormal values considered medically important by the Sponsor according to predefined criteria/thresholds based on literature review. The incidence of PCSA vital signs on Days 8, 15, and 29 (or day of discharge) will be summarised overall and by their baseline normality status (normal/abnormal).

9.5.3 Other Analyses

Where appropriate (if data collected), PK, pharmacodynamic, and biomarker exploratory analyses will be described in the SAP finalised before database lock. Drug concentration versus response variables may be graphically displayed for select endpoints. Exposure-response data may be obtained from this study and may be modelled and/or combined with data from other studies and used for modelling and simulations. If modelling and/or simulations are performed, these will be presented separately from the main clinical study report (CSR).

9.5.4 Missing Data

All patients recruited into the study will be accounted for, including those who did not complete the study along with the reasons for withdrawal. Patients who withdraw from the study will have the reasons of withdrawal collected in the eCRF. Non-completers in the endpoints of the response rate analyses will be treated as non-responders. More details will be described in the SAP before database lock.

9.6 Interim Analyses

An interim analysis will be performed at the end of Stage 1 to determine if each candidate agent will be carried forward to the confirmatory Stage 2 based on the comparison with the SoC arm. The assessment will be based on the totality of data and the evaluation carried out by the Steering Committee. Full details will be given in the Steering Committee charter.

In addition, the primary endpoint, analysis method, and corresponding sample size to use in Stage 2 will be re-evaluated based on the data from Stage 1 as well as relevant external information.

9.7 Review Committees

9.7.1 Steering Committee (Scientific Review Committee)

A Steering Committee has been assembled for the executive oversight and supervision of this ACCORD program. The Steering Committee will serve this role through regular scheduled meetings or teleconferences and, if necessary, additional ad hoc meetings.

The Steering Committee will be expected to:

- Evaluate interim analysis data to make decisions on further progression of the candidate agents within the study.
- Provide guidance, advice, and recommendations to the ACCORD program on relevant clinical issues related to the strategy, implementation, and conduct of the study. This may include, but not necessarily be limited to:
    - Advice on the strategy and design of the ACCORD Master Protocol and sub-protocols and any subsequent amendments or revisions.
    - Advice on issues of study enrolment including patient accrual, number and location of investigator sites, recruitment goals, and patient eligibility/ineligibility issues.
    - Advice on issues relating to the clinical conduct of the protocol including protocol violations/deviations and investigative site or Ethics Committee/Institutional Review Board concerns/issues, and regulatory engagement.
    - Advice on safety issues.

9.7.2 Independent Data and Safety Monitoring Committee

An independent IDMC will be established for this study to assess safety on an ongoing basis throughout the study. The committee will be established for the purpose of objectively monitoring the safety data and will not include any assessment of efficacy data. The IDMC members will perform ongoing safety surveillance and provide recommendations to the Steering Committee regarding study conduct.

The IDMC will consist of at least 3 independent experts appointed by the Steering Committee based on their expertise. IDMC members will not be Investigators in the study, nor will they have any conflict of interest with the Steering Committee, candidate agent Sponsors, or its designee.

Further details (eg, frequency of data reviews and study committee composition and membership) will be provided in the IDMC charter. The charters will define the criteria, frequency of reviews, data, and source documentation required to adjudicate all events.

10.0 REFERENCES

1. Hamming I, Timens W, Bulthuis ML, Lely AT, Navis G, van Goor H. Tissue distribution of ACE2 protein, the functional receptor for SARS coronavirus. A first step in understanding SARS pathogenesis. J Pathol. 2004 Jun;203(2):631-7.

2. Tang X, Wu C, Li X, et al. On the origin and continuing evolution of SARS-CoV-2. National Science Review, https://doi.org/10.1093/nsr/nwaa036.

3. Royal College of Physicians. National Early Warning Score (NEWS) 2: Standardising the Assessment of Acute-illness Severity in the NHS – Updated Report of a Working Party. Royal College of Physicians: London, 2017. https://www.rcplondon.ac.uk/file/8504/download [accessed 06 April 2020]

4. Cao B, Wang Y, Wen D, et al. A Trial of Lopinavir-Ritonavir in Adults Hospitalized with Severe Covid-19. N Engl J Med. 2020 Mar 18. doi: 10.1056/NEJMoa2001282. [Epub ahead of print].

11.0 APPENDICES

Appendix 1 Abbreviations

**Abbreviation**	**Definition**
ACE	Angiotensin-converting enzyme
AE	Adverse event
AESI	Adverse events of special interest
ALT	Alanine aminotransferase
AST	Aspartate aminotransferase
CIOMS	Council for International Organizations of Medical Sciences
CONSORT	Consolidated Standards of Reporting Trials
ECG	Electrocardiogram
ECMO	Extracorporeal membrane oxygenation
eCRF	Electronic case report form
EDTA	Ethylenediaminetetraacetic acid
FiO_2	Fraction of inspired oxygen
FSH	Follicle stimulating hormone
GCP	Good Clinical Practice
HIPAA	Health Insurance Portability and Accountability Act
HR	Hazard ratio
HRA	Health Research Authority
HRT	Hormone replacement therapy
ICF	Informed consent form
ICH	International Council for Harmonisation
ICU	Intensive care unit
IDMC	Independent Data and Safety Monitoring Committee
ITT	Intention to Treat
MedDRA	Medical Dictionary for Regulatory Activities
NEWS2	National Early Warning Score 2
PBMC	Peripheral blood mononuclear cell
PCR	Polymerase chain reaction
PCSA	Potentially clinically significant abnormality
PDS	Pharmacodynamic Analysis Set
PK	Pharmacokinetic
PKS	Pharmacokinetic Analysis Set

Abbreviation	Definition
PT	Preferred term
QTcF	QTc interval according to Fridericia correction
REC	Research ethics committee
RRT	Renal replacement therapy
SAE	Serious adverse event
SAP	Statistical Analysis Plan
SARS-CoV-2	Severe acute respiratory syndrome coronavirus 2
SoA	Schedule of Activities
SoC	Standard of care
SpO_2	Oxygen saturation
SUSAR	Suspected unexpected serious adverse reactions
TDCC	Tissue Directory and Coordination Centre
TEAE	Treatment-emergent adverse event
ULN	Upper limit of normal

Appendix 2 Regulatory, Ethical, and Study Oversight Considerations

Protocol Compliance

The Investigator agrees to comply with the requirements of the Protocol and Good Clinical Practice. Prospective, planned deviations or waivers to the protocol are not allowed under the UK regulations on Clinical Trials and must not be used; eg, it is not acceptable to enrol a patient if they do not meet the eligibility criteria or restrictions specified in the protocol.

Accidental protocol deviations can happen at any time. They must be adequately documented on the relevant forms and reported to the Chief Investigator and Sponsor immediately.

Deviations from the protocol, which are found to frequently recur, are not acceptable and will require immediate action by the Sponsor. Frequent non-compliances could potentially be classified as a serious breach.

Regulatory and Ethical Considerations

- This study will be conducted in accordance with the Master Protocol and sub-protocols and with the following:
  - Consensus ethical principles derived from international guidelines including the Declaration of Helsinki and Council for International Organizations of Medical Sciences (CIOMS) International Ethical Guidelines.
  - Applicable International Council for Harmonisation (ICH) Good Clinical Practice (GCP) Guidelines.
  - Applicable laws and regulations.
- The Master Protocol, sub-protocol, protocol amendments, ICF, Investigator Brochures, and other relevant documents (eg, advertisements) must be approved by the appropriate regulatory body, Health Research Authority (HRA), and REC before the study, or a new study arm introducing a candidate agent, is initiated.
- Any substantial amendments to the protocol will not be implemented until HRA/REC have provided the relevant authorisations, except for changes necessary to eliminate an immediate hazard to patients.
- All correspondence with the HRA and the REC will be retained in the Trial Master File and the Investigator Site File (maintained by the site).
- An annual progress report will be submitted to the REC within 30 days of the anniversary date on which the favourable opinion was given, and annually until the study is declared ended.
- Within 90 days after the end of the study (as defined in Section 4.4), the Chief Investigator/Sponsor will ensure that the HRA and the main REC are notified that the study has finished. If the study is terminated prematurely, those reports will be made within 15 days after the end of the study.

- The Chief Investigator will supply the Sponsor with a summary report of the study, which will then be submitted to the main REC within 1 year after the end of the study.
- All results will be published on a publicly accessible database.
- After reading the protocol, each Investigator will sign the protocol signature page and send a copy of the signed page to the Sponsor or representative (Appendix 7). The study will not start at any study centre at which the Investigator has not signed the protocol.

Financial Disclosure

Investigators and sub-Investigators will provide the Sponsor with sufficient, accurate financial information as requested to allow the Sponsor to submit complete and accurate financial certification or disclosure statements to the appropriate regulatory authorities. Investigators are responsible for providing information on financial interests during the course of the study and for 1 year after completion of the study.

Indemnity

The sponsor of the study is University Hospital Southampton NHS Foundation Trust. For NHS sponsored research HSG (96) 48 reference no.2 refers. If there is negligent harm during the clinical study when the NHS body owes a duty of care to the person harmed, NHS Indemnity covers NHS staff, medical academic staff with honorary contracts, and those conducting the study. NHS Indemnity does not offer no-fault compensation and is unable to agree in advance to pay compensation for non-negligent harm. Ex-gratia payments may be considered in the case of a claim.

Informed Consent Process

Note: Informed consent will be obtained electronically, using an electronic device with a screen that will be cleaned between users.

By participating in the study participants also consent to all the secondary research endpoints.

Some patients will be unable to give consent themselves, and if relatives are unable to visit the patients in person due to COVID-19 restrictions, it may not be possible to obtain legally authorised consent in person; in these circumstances a legally authorised representative can give assent via a remote process. The legally authorised representative will be contacted by telephone to discuss the patient's condition; they will then be sent an e-mail invitation about the study, and they can sign the ICF remotely. Study staff will be available by telephone to answer questions, and can counter-sign the document.

In the UK, the law allows adults with incapacity to be recruited into clinical trials such as this one without prior consent in emergency situations if:

- Treatment needs to be given urgently;

- It is also necessary to take urgent action to administer the study treatment for the purposes of the trial;
- It is not reasonably practicable to obtain consent from a legal representative;
- The procedure is approved by a National Health Service Research Ethics Committee;
- Consent is sought from a legally authorised representative as soon as possible.

The Investigator or his/her representative will explain the nature of the study to the patient or his/her legally authorised representative and answer all questions regarding the study.

Patients must be informed that their participation is voluntary. Patients or their legally authorised representative will be required to sign a statement of informed consent that meets local requirements, where applicable, and the REC or study centre.

The medical record must include a statement that written informed consent was obtained before the patient was entered in the study and the date the written consent was obtained. The authorised person obtaining the informed consent must also sign the ICF.

Patients must be re-consented to the most current version of the ICF(s) during their participation in the study.

A copy of the ICF(s) must be provided to the patient or the patient's legally authorised representative.

Data Protection

- Patients will be assigned a unique identifier by the Sponsor. Any patient records or datasets that are transferred to the Sponsor will contain the identifier only; patient names or any information which would make the patient identifiable will not be transferred.
- The patient must be informed that his/her personal study-related data will be used by the Sponsor in accordance with local data protection law. The level of disclosure must also be explained to the patient.
- The patient must be informed that his/her medical records may be examined by Clinical Quality Assurance auditors or other authorised personnel appointed by the Sponsor, by appropriate REC members, and by inspectors from regulatory authorities.
- The ICF will incorporate (or, in some cases, be accompanied by a separate document incorporating) wording that complies with relevant data protection and privacy legislation, that also explains that his/her medical records (past and future) may be linked to their study data and accessed and examined for healthcare research, and that participant data may be stored and processed in the cloud or outside the country.
- The ICF will explain that the individual genotype results will not be returned to patients, provided to any insurance company, to any employer, their family members, general physician, or any other third party, unless required to do so by law. Incidental findings in the

genome data will not be reported back to the individual; these data are research data and results will not be confirmed by accredited clinical genetics testing laboratories.

- Participants will not receive any financial benefit from the use of their samples or data in research. Samples and data may be processed and used by commercial entities as well as government or academic entities.
- Extra precautions are taken to preserve confidentiality and prevent genetic data being linked to the identity of the patient. In exceptional circumstances, however, certain individuals might see both the genetic data and the personal identifiers of a patient. For example, in the case of a medical emergency, the Sponsor or representative physician or an Investigator might know a patient's identity and also have access to his or her genetic data. Also regulatory authorities may require access to the relevant files.

Administrative Structure

The University Hospital Southampton NHS Foundation Trust will be the overall Sponsor of the platform study and of each sub-protocol, and is the organisation that it taking legal responsibility for the study.

The Chief Investigator is Professor Tom Wilkinson, Clinical & Experimental Sciences, Southampton General Hospital, Tremona Road, Southampton, SO16 6YD.

The IQVIA Therapeutic Medical Advisor (Medical Monitor) is available for 24 hours a day/7 days a week urgent contact. If the IQVIA Therapeutic Medical Advisor is not able to provide 24/7 services for a period longer than 2 hours (eg, for international business travel) or during vacations, adequate back-up will be arranged and communicated.

The administrative structure will be documented in more detail in the Trial Master File.

Dissemination of Clinical Study Data

The results of the study should be reported within 1 year from the end of the clinical study. Irrespective of the outcome, the Sponsor will submit to the European Union database a summary of the results of the clinical study within 1 year from the end of the clinical study. It shall be accompanied by a summary written in a manner that is understandable to laypersons.

Data Quality Assurance

- All patient data relating to the study will be recorded on eCRFs unless transmitted to the Sponsor or designee electronically (eg, laboratory data). The Investigator is responsible for verifying that data entries are accurate and correct by physically or electronically signing the eCRF.
- The Investigator must maintain accurate documentation (source data) that supports the information entered in the eCRF.

- The Investigator must permit study-related monitoring, audits, REC review, and regulatory agency inspections and provide direct access to source data documents.
- The Sponsor or designee is responsible for the data management of this study including quality checking of the data.
- Study monitors will perform ongoing source data review to confirm that data entered into the eCRF by authorised study centre personnel are accurate, complete, and verifiable from source documents; that the safety and rights of patients are being protected; and that the study is being conducted in accordance with the currently approved protocol and any other study agreements, ICH GCP, and all applicable regulatory requirements.
- Records and documents, including signed ICFs, pertaining to the conduct of this study must be retained by the Investigator for 25 years after study completion unless local regulations or institutional policies require a longer retention period. No records may be destroyed during the retention period without the written approval of the Sponsor. No records may be transferred to another location or party without written notification to the Sponsor.

Source Documents

The Investigator/institution should maintain adequate and accurate source documents and study records that include all pertinent observations on each of the study centre's patients. Source data should be attributable, legible, contemporaneous, original, accurate, and complete. Changes to source data should be traceable, should not obscure the original entry, and should be explained if necessary (eg, via an audit trail).

- Source documents provide evidence for the existence of the patient and substantiate the integrity of the data collected. Source documents are filed at the Investigator's study centre.
- Data reported on the eCRF or entered in the eCRF that are transcribed from source documents must be consistent with the source documents or the discrepancies must be explained. The Investigator may need to request previous medical records or transfer records, depending on the study. Also, current medical records must be available.
- Definition of what constitutes source data can be found in the eCRF completion guidelines.

Study and Study Centre Closure

The Sponsor designee reserves the right to close the study centre or terminate the study at any time for any reason at the sole discretion of the Sponsor. Study centres will be closed upon study completion. A study centre is considered closed when all required documents and study supplies have been collected and a study centre closure visit has been performed.

The Investigator may initiate study centre closure at any time, provided there is reasonable cause and sufficient notice is given in advance of the intended termination.

Reasons for the early closure of a study centre by the Sponsor or Investigator may include but are not limited to:

- Failure of the Investigator to comply with the protocol, the requirements of the REC or local health authorities, the Sponsor's procedures, or GCP guidelines.
- Inadequate recruitment of patients by the Investigator.
- Discontinuation of further study treatment development.

Publication Policy

The data generated by this study are confidential information of the Sponsor. The Sponsor will make the results of the study publicly available. The publication policy with respect to the Investigator and study centre will be set forth in the Clinical Trial Agreement.

- The results of this study may be published or presented at scientific meetings. If this is foreseen, the Investigator agrees to submit all manuscripts or abstracts to the Sponsor before submission. This allows the Sponsor to protect proprietary information and to provide comments.
- The Sponsor will comply with the requirements for publication of study results. In accordance with standard editorial and ethical practice, the Sponsor will generally support publication of multicentre studies only in their entirety and not as individual study centre data. In this case, a Coordinating Investigator will be designated by mutual agreement.
- Authorship will be determined by mutual agreement and in line with International Committee of Medical Journal Editors authorship requirements.

Appendix 3 Clinical Laboratory Tests

The minimum tests to be performed are detailed in Table 5. Any additional tests that are specific for the candidate agent will be detailed in the corresponding sub-protocol. Clinical laboratory tests will be performed at a local laboratory.

Protocol-specific requirements for inclusion or exclusion of patients are detailed in Section 5.0 of the protocol.

Additional tests may be performed at any time during the study as determined necessary by the Investigator or required by local regulations.

Changes to some laboratory parameters are anticipated for any patients moving on to ECMO therapy.

Investigators must document their review of each laboratory safety report.

Table 5 Protocol-required Safety Laboratory Assessments

**Laboratory Assessments**	**Parameters**
Haematology	Platelet Count<br>Haemoglobin<br>White blood cell count with differential:<br>Neutrophils<br>Lymphocytes<br>Monocytes<br>Eosinophils<br>Basophils
Coagulation	D-dimer test (if possible)<br>Fibrinogen<br>Activated partial thromboplastin time (aPTT)<br>Prothrombin time (PT)<br>International Normalised Ratio (INR)
Clinical Chemistry	Potassium<br>Sodium<br>Calcium<br>Magnesium<br>Phosphate<br>Alkaline phosphatase<br>Bicarbonate<br>Creatinine<br>Creatine kinase (MB fraction)<br>Glucose<br>Total bilirubin<br>Aspartate aminotransferase (AST)<br>Alanine aminotransferase (ALT)<br>Gamma-glutamyl transferase (GGT)<br>C-reactive protein<br>Ferritin<br>Triglycerides<br>Lactate dehydrogenase (LDH)<br>Troponin

Appendix 4 Adverse Events: Definitions and Procedures for Recording, Evaluating, Follow-up, and Reporting

Definition of AE

**AE Definition**
• An adverse event (AE) is any untoward medical occurrence in a patient or patient, temporally associated with the use of study treatment, whether or not considered related to the study treatment.<br>• NOTE: An AE can therefore be any unfavourable and unintended sign (including an abnormal laboratory finding), symptom, or disease (new or exacerbated) temporally associated with the use of study treatment.

**Events Meeting the AE Definition**
• Any abnormal laboratory test results (haematology, clinical chemistry, or urinalysis) or other safety assessments (eg, electrocardiogram, radiological scans, vital signs measurements), including those that worsen from baseline, considered clinically significant in the medical and scientific judgment of the Investigator (ie, not related to progression of underlying disease).<br>• Exacerbation of a chronic or intermittent pre-existing condition including either an increase in frequency and/or intensity of the condition.<br>• New conditions detected or diagnosed after study treatment administration even though it may have been present before the start of the study.<br>• Signs, symptoms, or the clinical sequelae of a suspected drug-drug interaction.<br>• Signs, symptoms, or the clinical sequelae of a suspected overdose of either study treatment or a concomitant medication. Overdose per se will not be reported as an AE/serious adverse event (SAE) unless it is an intentional overdose taken with possible suicidal/self-harming intent. Such overdoses should be reported regardless of sequelae.<br>• "Lack of efficacy" or "failure of expected pharmacological action" per se will not be reported as an AE or SAE. Such instances will be captured in the efficacy assessments. However, the signs, symptoms, and/or clinical sequelae resulting from lack of efficacy will be reported as AE or SAE if they fulfil the definition of an AE or SAE.

**Events NOT Meeting the AE Definition**
• Any clinically significant abnormal laboratory findings or other abnormal safety assessments which are associated with the underlying disease, unless judged by the Investigator to be more severe than expected for the patient’s condition.<br>• The disease/disorder being studied or expected progression, signs, or symptoms of the disease/disorder being studied, unless more severe than expected for the patient’s condition.<br>• Medical or surgical procedure (eg, endoscopy, appendectomy): the condition that leads to the procedure is the AE.<br>• Situations in which an untoward medical occurrence did not occur (social and/or convenience admission to a hospital).<br>• Anticipated day-to-day fluctuations of pre-existing disease(s) or condition(s) present or detected at the start of the study that do not worsen.

Definition of SAE

If an event is not an AE per definition above, then it cannot be an SAE even if serious conditions are met (eg, hospitalisation for signs/symptoms of the disease under study, death due to progression of disease).

An SAE is defined as any untoward medical occurrence that, at any dose:
**a) Results in death**
**b) Is life-threatening**<br>The term ‘life-threatening’ in the definition of “serious” refers to an event in which the patient was at risk of death at the time of the event. It does not refer to an event, which hypothetically might have caused death, if it were more severe.
**c) Requires inpatient hospitalisation or prolongation of existing hospitalisation**<br>In general, hospitalisation signifies that the patient has been detained (usually involving at least an overnight stay) at the hospital or emergency ward for observation and/or treatment that would not have been appropriate in the physician’s office or outpatient setting. Complications that occur during hospitalisation are AEs. If a complication prolongs hospitalisation or fulfils any other serious criteria, the event is serious. When in doubt as to whether “hospitalisation” occurred or was necessary, the AE should be considered serious.<br>Hospitalisation for elective treatment of a pre-existing condition that did not worsen from baseline is not considered an AE.
**d) Results in persistent disability/incapacity**<br>• The term disability means a substantial disruption of a person’s ability to conduct normal life functions.<br>• This definition is not intended to include experiences of relatively minor medical significance such as uncomplicated headache, nausea, vomiting, diarrhoea, influenza, and accidental trauma (eg, sprained ankle) which may interfere with or prevent everyday life functions but do not constitute a substantial disruption.
**e) Is a congenital anomaly/birth defect**
**f) Other situations:**<br>• Medical or scientific judgment should be exercised in deciding whether SAE reporting is appropriate in other situations such as important medical events that may not be immediately life-threatening or result in death or hospitalisation but may jeopardise the patient or may require medical or surgical intervention to prevent 1 of the other outcomes listed in the above definition. These events should usually be considered serious.<br>Examples of such events include invasive or malignant cancers, intensive treatment in an emergency room or at home for allergic bronchospasm, blood dyscrasias, or convulsions that do not result in hospitalisation, or development of drug dependency or drug abuse.

Recording and Follow-up of AE and/or SAE

AE and SAE Recording
• When an AE/SAE occurs, it is the responsibility of the Investigator to review all documentation (eg, hospital progress notes, laboratory reports, and diagnostics reports) related to the event.<br>• The Investigator will then record all relevant AE/SAE information in the eCRF. Each event must be recorded separately.<br>• It is **not** acceptable for the Investigator to send photocopies of the patient's medical records to the CRO/Sponsor in lieu of completion of the AE/SAE eCRF page.<br>• There may be instances when copies of medical records for certain cases are requested by the CRO/Sponsor. In this case, all patient identifiers, with the exception of the patient number, will be redacted on the copies of the medical records before submission to the CRO/Sponsor.<br>• The Investigator will attempt to establish a diagnosis of the event based on signs, symptoms, and/or other clinical information. Whenever possible, the diagnosis (not the individual signs/symptoms) will be documented as the AE/SAE.
**Assessment of Intensity**
The Investigator will make an assessment of intensity for each AE and SAE reported during the study and assign it to 1 of the following Common Terminology Criteria for Adverse Events (CTCAE) grades:<br>• Grade 1 Mild; asymptomatic or mild symptoms; clinical or diagnostic observations only; intervention not indicated.<br>• Grade 2 Moderate; minimal, local or noninvasive intervention indicated; limiting age appropriate instrumental activities of daily living (preparing meals, shopping for groceries or clothes, using the telephone, managing money, etc).<br>• Grade 3 Severe or medically significant but not immediately life-threatening; hospitalisation or prolongation of hospitalisation indicated; disabling; limiting self care activities of daily living (bathing, dressing and undressing, feeding self, using the toilet, taking medications, and not bedridden).<br>• Grade 4 Life-threatening consequences; urgent intervention indicated.<br>• Grade 5 Death related to AE.<br>An event is defined as 'serious' when it meets at least 1 of the predefined outcomes as described in the definition of an SAE, NOT when it is rated as severe (Grade 3 and higher).

Assessment of Causality
• The Investigator is obligated to assess the relationship between study treatment and each occurrence of each AE/SAE. The AE must be characterised as related or unrelated.<br>o "Related" conveys that there are facts, evidence, and/or arguments to suggest a causal relationship for the individual case.<br>o "Unrelated" is used if there is not a reasonable possibility that the study treatment caused the AE.<br>• The Investigator will use clinical judgment to determine the relationship.<br>• Alternative causes, such as underlying disease(s), concomitant therapy, and other risk factors, as well as the temporal relationship of the event to study treatment administration will be considered and investigated.<br>• The Investigator will also consult the Investigator's Brochure (IB) and/or Product Information, for marketed products, in his/her assessment.<br>• For each AE/SAE, the Investigator must document in the medical notes that he/she has reviewed the AE/SAE and has provided an assessment of causality.

• There may be situations in which an SAE has occurred and the Investigator has minimal information to include in the initial report to the CRO/Sponsor. However, it is very important that the Investigator always make an assessment of causality for every event before the initial transmission of the SAE data to the CRO/Sponsor.<br>• The Investigator may change his/her opinion of causality in light of follow-up information and send an SAE follow-up report with the updated causality assessment.<br>• The causality assessment is one of the criteria used when determining regulatory reporting requirements.

**Follow-up of AEs and SAEs**
• The Investigator is obligated to perform or arrange for the conduct of supplemental measurements and/or evaluations as medically indicated or as requested by the CRO/Sponsor to elucidate the nature and/or causality of the AE or SAE as fully as possible. This may include additional laboratory tests or investigations, histopathological examinations, or consultation with other health care professionals.<br>• If a patient dies during participation in the study or during a recognised follow-up period, the Investigator will provide the CRO/Sponsor with a copy of any postmortem findings including histopathology<br>• New or updated information will be recorded in the originally completed eCRF.<br>• The Investigator will submit any updated SAE data to the Sponsor within 24 hours of receipt of the information.

Reporting of SAEs

**SAE Reporting to the CRO/Sponsor via an Electronic Data Collection Tool**
• The primary mechanism for reporting an SAE to the CRO/Sponsor will be the electronic data collection tool.<br>• If the electronic system is unavailable for more than 24 hours, then the study centre will use the paper SAE data collection tool (see next section).<br>• The study centre will enter the SAE data into the electronic system as soon as it becomes available.<br>• After the study is completed at a given study centre, the electronic data collection tool will be taken off-line to prevent the entry of new data or changes to existing data.<br>• If a study centre receives a report of a new SAE from a patient or receives updated data on a previously reported SAE after the electronic data collection tool has been taken off-line, then the study centre can report this information on a paper SAE form (see next section) or to the medical monitor/SAE coordinator by telephone.

**SAE Reporting to CRO/Sponsor via Paper Case Report Form**
• Facsimile transmission of the SAE paper case report form is the preferred method to transmit this information to the medical monitor or the SAE coordinator.<br>• In rare circumstances and in the absence of facsimile equipment, notification by telephone is acceptable with a copy of the SAE data collection tool sent by overnight mail or courier service.<br>• Initial notification via telephone does not replace the need for the Investigator to complete and sign the SAE eCRF pages within the designated reporting time frames.

Appendix 5 Collection of Pregnancy Information

Definitions:

Woman of Childbearing Potential (WOCBP)

A woman is considered fertile following menarche and until becoming postmenopausal unless permanently sterile (see below).

Women in the following categories are not considered WOCBP

1. Premenarchal
2. Premenopausal female with 1 of the following:
    a) Documented hysterectomy.
    b) Documented bilateral salpingectomy.
    c) Documented bilateral oophorectomy.
    Note: Documentation can come from the study centre personnel's: review of the patient's medical records, medical examination, or medical history interview.
3. Postmenopausal female:
    a) A postmenopausal state is defined as no menses for 12 months without an alternative medical cause. A high follicle stimulating hormone (FSH) level in the postmenopausal range may be used to confirm a postmenopausal state in women not using hormonal contraception or hormonal replacement therapy (HRT). However, in the absence of 12 months of amenorrhea, a single FSH measurement is insufficient.
    b) Females on HRT and whose menopausal status is in doubt will be required to use 1 of the non-oestrogen hormonal highly effective contraception methods if they wish to continue their HRT during the study. Otherwise, they must discontinue HRT to allow confirmation of postmenopausal status before study enrolment.

Pregnancy Testing:

WOCBP should only be included after a confirmed menstrual period and a negative highly sensitive pregnancy test.

Pregnancy testing will also be performed whenever a menstrual cycle is missed or when pregnancy is otherwise suspected.

Collection of Pregnancy Information

The Investigator will collect pregnancy information on any female patient who becomes pregnant while participating in this study. Information will be recorded on the appropriate form and submitted to the Sponsor within 24 hours of learning of a patient's pregnancy. The patient will be followed to determine the outcome of the pregnancy. The Investigator will collect follow-up information on the patient and the neonate and the information will be forwarded to the Sponsor. Generally, follow-up will not be required for longer than 6 to 8 weeks beyond the

estimated delivery date. Any termination of pregnancy will be reported, regardless of foetal status (presence or absence of anomalies) or indication for the procedure.

While pregnancy itself is not considered to be an AE or SAE, any pregnancy complication or elective termination of a pregnancy will be reported as an AE or SAE. A spontaneous abortion is always considered to be an SAE and will be reported as such.

Any poststudy pregnancy-related SAE considered reasonably related to the study treatment by the Investigator will be reported to the Sponsor as described in Section 8.4.4. While the Investigator is not obligated to actively seek this information in former patients, he or she may learn of an SAE through spontaneous reporting.

Continuation of study treatment may be allowed, as COVID-10 is a high mortality disease.

Appendix 6 Genetics

Use/Analysis of DNA

Genetic variation in a patient's DNA may impact their response to study treatment, susceptibility to, and severity and progression of disease. Variable response to study treatment may be due to genetic determinants that impact drug absorption, distribution, metabolism, and excretion; mechanism of action of the drug; disease aetiology; and/or molecular subtype of the disease being treated. Therefore, where local regulations and REC allow, samples will be collected for host DNA analysis from consenting patients.

This analysis may provide insights into host variation that may help explain COVID-19 outcomes, drug response or toxicity and provide data to develop further biomarkers and assay for identification of patient sub-groups. Genomics data may be correlated with other functional assays, eg, antibody profiling, that may form part of downstream clinical studies. It may be possible to stratify study results by viral strain of COVID-19 if variation and mutations are identified or co-infection occurs. This may have the benefit of contributing to global knowledge of the biology of SARS-CoV-2 infection and disease, allowing development of research assets for future use, thus supporting research to shorten the COVID-19 pandemic.

The results of genetic analyses may be reported in the CSR or in a separate study summary.

Sample usage for the exploratory endpoints of host genome, viral genome, and serology analysis or other future healthcare research using residual sample materials, will be overseen managed by the independent, publicly funded, Tissue Directory and Coordination Centre (TDCC), hosted at Nottingham University.

The samples collected for secondary translational research endpoints will follow a documented chain of custodianship, with the transfer of the custodianship of the samples from the study Sponsor to the laboratories performing the sample analysis.

Any residual samples retained for future research will be distributed through a documented managed sample access process with scientific review by a scientific review body hosted at TDCC.

Samples will be stored in a secure storage space in line with recommended guidelines for infectious substance handling, and with adequate measures to protect confidentiality.

The samples will be retained while research on COVID-19 continues but no longer than 10 years or other period as per local requirements.

Appendix 7 Signature of Investigator

PROTOCOL TITLE: ACCORD-2: A Multicentre, Seamless, Phase 2 Adaptive Randomisation Platform Study to Assess the Efficacy and Safety of Multiple Candidate Agents for the Treatment of COVID-19 in Hospitalised Patients

PROTOCOL NO: ACCORD-2-001

VERSION: Protocol Amendment 01

This protocol is a confidential communication of the Sponsor. I confirm that I have read this protocol, I understand it, and I will work according to this protocol. I will also work consistently with the ethical principles that have their origin in the Declaration of Helsinki and that are consistent with Good Clinical Practices and the applicable laws and regulations. Acceptance of this document constitutes my agreement that no unpublished information contained herein will be published or disclosed without prior written approval from the Sponsor.

Instructions to the Investigator: Please SIGN and DATE this signature page. PRINT your name, title, and the name of the study centre in which the study will be conducted. Return the signed copy to the CRO/Sponsor.

I have read this protocol in its entirety and agree to conduct the study accordingly:

Signature of Investigator: ___________________________ Date: ________

Printed Name: ____________________________

Investigator Title: ____________________________

Name/Address of Centre: ____________________________

____

____
