# Supplementary material for: ACCORD: A Multicentre, Seamless, Phase 2 Adaptive Randomisation Platform Study to Assess the Efficacy and Safety of Multiple Candidate Agents for the Treatment of COVID-19 in Hospitalised Patients: A structured summary of a study protocol for a randomised controlled trial
Source: Trials. 2020 Jul 31;21:691. doi: 10.1186/s13063-020-04584-9 (PMC7393340; doi:10.1186/s13063-020-04584-9)
Supplement: Supplementary file 1 — Additional file 1. [file 13063_2020_4584_MOESM1_ESM.zip › ACCORD-2-002_Bemcentinib_SubprotocolR0.pdf]

TITLE PAGE

Master Protocol Title: ACCORD-2: A Multicentre, Seamless, Phase 2 Adaptive Randomisation Platform Study to Assess the Efficacy and Safety of Multiple Candidate Agents for the Treatment of COVID-19 in Hospitalised Patients

Sub-protocol Number: ACCORD-2-002

Sub-protocol for Candidate Agent: Bemcentinib

Study Phase: 2

Sponsor Name: University Hospital Southampton NHS Foundation Trust

Legal Registered Address: **Southampton General Hospital**
Level E, Laboratory & Pathology Block, SCBR - MP138
Tremona Road
Southampton SO16 6YD, UK

Regulatory Agency Identifying Number(s): EudraCT: 2020-001736-95

IRAS Number: **282769**

RHM Number: **MED1711**

Date of Sub-protocol: 24 April 2020

Version: Protocol Amendment 02, Final Version 2

Caution: In accordance with cardiac exclusion criteria, in particular Exclusion 13, drugs known to cause QT prolongation should be discontinued/replaced, with sufficient time (5 half-lives) for washout.

Patients in these categories of potential risk should be discussed directly by telephone with BerGenBio:

Prof Hani Gabra, BerGenBio Chief Medical Officer: +44 7810 576112

Dr Akil Jackson, BerGenBio Medical Director: +44 7810 575037

Sponsor Signatory:

I have read this sub-protocol in its entirety and agree to conduct this part of the study accordingly:

**Professor Tom Wilkinson MA Cantab MBBS PhD FRCP**	**Date**
**Professor of Respiratory Medicine and Honorary NHS Consultant Physician**	

TABLE OF CONTENTS

TABLE OF FIGURES 4

PROTOCOL AMENDMENT HISTORY 5

1.0 SUB-PROTOCOL SUMMARY 6
- **1.1 Overview of Sub-protocol** 6
- **1.2 Schedule of Activities** 8

2.0 BACKGROUND/RATIONALE IN SUPPORT OF BEMCENTINIB FOR COVID-19 12
- **2.1 Preliminary Efficacy Data vs with SARS-CoV-2 In vitro** 13
- **2.2 Dose Justification for Bemcentinib** 14
- **2.3 Human Experience from Trials in Cancer Patients** 16

3.0 STUDY POPULATION 18
- **3.1 Inclusion Criteria** 18
- **3.2 Eligibility Criteria** 18

4.0 STUDY ASSESSMENTS AND PROCEDURES 20
- **4.1 Efficacy Assessments** 20
- **4.2 Safety Assessments** 20
  - 4.2.1 Adverse Events 21
  - 4.2.2 Clinical Safety Laboratory Assessments 21
- **4.3 Pharmacokinetic Assessments** 21
- **4.4 Pharmacodynamic Assessments** 21

5.0 STUDY TREATMENT 22
- **5.1 Conclusions and Risk Benefit Statement** 22
- **5.2 Bemcentinib Drug Administration** 22
- **5.3 Dose Modifications and Toxicity Management** 23
- **5.4 Prohibited Concomitant Medications** 23
- **5.5 Study Drug Information** 23
  - 5.5.1 Study Medication 23
  - 5.5.2 Bemcentinib Storage, Dispensing, and Destruction 24

6.0 REFERENCES 25

7.0 AMENDMENT HISTORY 27

8.0 APPENDICES 28
- **Appendix 1 Abbreviations** 28

Appendix 2 Prohibited Concomitant Medication Due to QT Prolongation Risk or CYP3A4 Interaction with Narrow Therapeutic Index 29
Appendix 3 Signature of Investigator 30

TABLE OF FIGURES

Figure 1 Bemcentinib Potently Inhibits Severe Acute Respiratory Syndrome Coronavirus 2 (SARS-CoV-2) Infection of Vero E6 Cells 14
Figure 2 Complete Inhibition of AXL Kinase Activation (p-Axl) in Two Acute Myeloid Leukaemia Patients Treated at the Recommended Phase 2 Dose (200 mg Maintenance Daily Dosing) 15
Figure 3 Bemcentinib Plasma Pharmacokinetics in Acute Myeloid Leukaemia Patients 16

PROTOCOL AMENDMENT HISTORY

Protocol Amendment 02 (dated 24 April 2020) replaces Protocol Amendment 01 for the sub-protocol for bemcentinib (dated 22 April 2020).

The amendment incorporates the following main changes:

- RHM number added to title page.
- Removed requirement that temperature needs to be taken orally.
- Clarified in Schedule of Activities footnotes that bemcentinib can be taken more than 2 hours after a light meal (and not just on an empty stomach).
- Inclusion criterion 3 modified from the Master Protocol, as the contraception methods will need to be used for 120 days (not 6 weeks) after termination of study therapy.
- Inclusion criterion 4 modified from the Master Protocol, as the guidance for lactating mothers will be for 120 days (not 6 weeks) after termination of study therapy.
- Additional exclusion criteria have been renumbered as X1, X2 etc (instead of 12, 13 etc) to make them a distinct numbering system from those in the Master Protocol, which are subject to amendment.

1.0 SUB-PROTOCOL SUMMARY

1.1 Overview of Sub-protocol

Bemcentinib is a small molecule AXL kinase inhibitor developed by BerGenBio ASA (the candidate agent owner) which has demonstrated potent and selective inhibition of AXL in biochemical and cell-based kinase inhibition assays. AXL is a recognised therapeutic target for the treatment of cancer. AXL regulates cancer cell survival and immunosuppressive modulatory effects, particularly in driving immunosuppressive features of the innate immune compartment in the tumour microenvironment, including activation of the myeloid derived suppressor cells, the M2 macrophage state, and suppressor regulatory dendritic cells that inhibit activated T cell function. Further, AXL is an important negative regulator of type I interferon (IFN) responses that are important for anti-cancer treatments. Bemcentinib is currently being developed for the treatment of cancer, particularly in non-small cell lung carcinoma, acute myeloid leukaemia (AML), myelodysplastic syndrome, and other cancers.

The AXL receptor mediates entry of enveloped viruses such as Zika and Ebola through “apoptotic mimicry”. Phosphatidylserine (PS) on the viral envelope is tethered to AXL by its bound ligand, growth arrest-specific 6 (GAS6), leading to viral internalisation. Viruses also activate AXL signal transduction that antagonises anti-viral type-I IFN responses. Preclinical data show that AXL-mediated internalization and IFN suppression may extend to severe acute respiratory syndrome coronavirus 2 (SARS-CoV-2) and that bemcentinib can inhibit coronavirus entry and replication. The hypothesis is therefore that bemcentinib is a potentially effective treatment for COVID-19 disease. Additionally the Sponsor’s understanding of the mechanism supports testing the hypothesis that bemcentinib may be an effective preventive/prophylactic therapy for COVID-19.

This sub-protocol describes the approach for study conduct of the bemcentinib arm within the ACCORD-2 multicentre, Phase 2 adaptive randomisation platform study to assess the efficacy and safety of multiple candidate agents for the treatment of COVID-19 in hospitalised patients.

Key differentiating features of this sub-protocol include the following:

(a) Bemcentinib is an oral 100-mg capsule, recommended Phase 2 dose (RP2D) is 200 mg given once daily, following a loading dose (400 mg) given once daily for 3 days. Although the Master Protocol plans for 15 days of treatment, this sub-protocol can provide up to 21 days of bemcentinib treatment if required for patients.

(b) A specific eligibility criterion has been added to exclude patients based on electrocardiogram (ECG) QT interval on screening above 450 msec (a lower exclusion limit than that stated in the Master Protocol, 500 msec), as well as significant cardiac comorbidity. Any concomitant medication with QT prolongation risk must be stopped at screening and direct discussion between the investigator and Sponsor Medical Monitors will allow support in risk management. Serial ECG measurements have been added in

order to pragmatically monitor any QT interval change with bemcentinib, coupled to a sparse pharmacokinetic (PK) sample schedule, in the hospitalised COVID-19 population. Within the clinical development programme to date, over 286 patients have been treated without any observed cardiac adverse events (AEs).

(c) Translational and pharmacodynamic (PD) sampling is included in this protocol. Detailed procedures for the collection, processing, storage and shipment of the samples will be provided in the Study Laboratory Manual.

(d) Selected secondary endpoints from the Master Protocol will be considered as key secondary endpoints for the purposes of this sub-protocol.

1.2 Schedule of Activities

	**Screening**	**Baseline**					
**Day (± Window)**	**Day -1 or Day 1**	**Day 1**	**Daily Until Hospital Discharge**	**Day 15[a] (±2 days)**	**Day 29[a] (±3 days)**	**Day 60[a] (±4 days) (Follow-up)**	**Day 90[a] (±6 days) (End of Study)**
ELIGIBILITY							
Informed consent	X						
Demographics	X						
Relevant medical history[b]	X						
Review SARS-CoV-2 diagnostic tests	X						
Inclusion and exclusion criteria	X						
STUDY INTERVENTION							
Randomisation		X					
Administration of bemcentinib[c]		Days 1-3 400 mg loading daily	X 200 mg maintenance	X			
Treatment with SoC		X	X				
STUDY PROCEDURES							
Clinical frailty score	X						
Diagnostic imaging (X-ray and/or computed tomography)	X						
Physical examination (including presenting symptoms, height, weight)	X						
Targeted physical examination (focused on lung auscultation)			X				

	**Screening**	**Baseline**					
**Day (± Window)**	**Day -1 or Day 1**	**Day 1**	**Daily Until Hospital Discharge**	**Day 15[a] (±2 days)**	**Day 29[a] (±3 days)**	**Day 60[a] (±4 days) (Follow-up)**	**Day 90[a] (±6 days) (End of Study)**
Vital signs, including temperature, pulse rate, blood pressure, respiratory rate, SpO_2		X[d]	X	X	X		
Clinical assessments[e]		X[d]	X	X	X		
Targeted medication review (including use of vasopressors)		X[d]	X	X	X		
Adverse event evaluation		X	X	X	X	X	X
Disease-related co-infection evaluation (including microbiologic/infectious agent assessment/results; bacteria, viral, fungi)		X	X				
Survival status		X	X	X	X	X	X
Blood gases and FiO_2 at worst PO_2[f]	X	X	X				
12-lead ECG[g]	X	X	See schedule below[g]	X			
SAFETY LABORATORY							
Haematology, chemistry, liver function tests, coagulation[h]	X[i]	X[d,j]	Days 3, 5, 8, 11 (all ±1 day) if hospitalised	X [k]	X [k]		
Pregnancy test for females of childbearing potential	X[i]						
RESEARCH LABORATORY							
Blood (SST) for exploratory inflammatory cytokine analysis		X	Day 8	X	X		
Blood (sodium heparin tube) for PBMC phenotyping[l]		X	Day 8	X	X		

	Screening	Baseline					
**Day (± Window)**	**Day -1 or Day 1**	**Day 1**	**Daily Until Hospital Discharge**	**Day 15[a] (±2 days)**	**Day 29[a] (±3 days)**	**Day 60[a] (±4 days) (Follow-up)**	**Day 90[a] (±6 days) (End of Study)**
Blood (EDTA) for SARS-CoV-2 PCR (qualitative and quantitative)		X	Days 3, 5, 8, 11 (all ±1 day) if hospitalised	X	X		
Oropharyngeal/nasal swab for SARS-CoV-2 PCR (qualitative and quantitative)		X	Days 3, 5, 8, 11 (all ±1 day) if hospitalised	X	X		
Saliva for SARS-CoV-2 PCR (qualitative and quantitative)		X	Days 3, 5, 8, 11 (all ±1 day) if hospitalised	X	X		
Blood (SST) for SARS-CoV-2 serology research (host response)		X	Day 8	X	X	X	
Blood (PAXGENE) for transcriptome analysis (host genome)[m]		X	Day 8	X			
Blood (EDTA) host genome (host DNA)[m]		X					
Mid-turbinate nasal swab viral genome[m]		X					
Blood samples for PD and translational studies[n]		X	See schedule below[n]	X	X	X	
PK sampling[n]	X		See schedule below[n]	X			

ECG=electrocardiogram; EDTA=ethylenediaminetetraacetic acid; FiO_2=fraction of inspired oxygen; PBMC=peripheral blood mononuclear cell; PCR=polymerase chain reaction; PD=pharmacodynamic; PK=pharmacokinetic; PO_2=partial pressure of oxygen; RT PCR=reverse transcription polymerase chain reaction; SARS-CoV-2= severe acute respiratory syndrome coronavirus 2; SoC=standard of care; SpO_2=oxygen saturation; SST=serum separator tube.

[a] These visits will be performed even if a patient has already been discharged. If discharged prior to scheduled visit, in-person visits are preferred, but recognising that quarantine and other factors may limit the patient's ability to return to the clinic, these visits may be conducted by telephone or with a home visit by study staff. For visits conducted by telephone, it will not be possible to perform some scheduled assessments (eg, vital signs). The Day 29 assessments will also be performed, where possible, for patients who discontinue the study prematurely.

[b] Medical history includes estimated date and time of first symptoms and number of co-morbidities (eg, respiratory, cardiovascular, metabolic, malignancy, endocrine, gastrointestinal, immunologic, renal).

[c] Although the Master Protocol plans for 15 days treatment, this sub protocol can provide up to 21 days bemcentinib treatment if required for patients. Bemcentinib should be taken once per day, in the morning, on an empty stomach or more than 2 hours after a light meal, with water. Patients should not consume anything other than water for at least 1 hour after taking study drug.

[d] Baseline assessments should be performed prior to study drug administration.

[e] Includes ordinal score, National Early Warning Score 2 (NEWS2), oxygen requirement, noninvasive or invasive ventilator requirement, including start and stop of low- or high-flow oxygen supply or of any form of ventilation etc.

[f] If done as part of SoC, blood gases results to be fully recorded with date and time.

[g] ECG to coincide with selected PK and PD sampling timepoints, ie, pre-loading dose (Day 1); then pre-maintenance dose and 6 hours postdose (Day 4). Subsequently predose on Days 8 and 15.

[h] For parameters, see Master Protocol

[i] Laboratory tests performed in the 48 hours prior to enrolment will be accepted for determination of eligibility.

[j] Any laboratory tests performed as part of routine clinical care within the specified visit window can be used for safety laboratory testing.

[k] Additional safety laboratory evaluations to be performed on Days 15 and 29 only if patient is still hospitalised.

[l] Samples collected for immediate laboratory processing and frozen storage.

[m] Samples collected dependent on capacity of study centre, need for reduced study burden on staff, and potentially limited access to patients.

[n] Procedures for the collection, processing, storage and shipment of the PK and PD samples will be provided in the Study Laboratory Manual. Sample collection to coincide with the ECGs, ie, pre-loading dose (Day 1); then pre-maintenance dose and 6 hours postdose (Day 4). Subsequently predose on Days 8 and 15.

2.0 BACKGROUND/RATIONALE IN SUPPORT OF BEMCENTINIB FOR COVID-19

Nonclinical in vitro and in vivo data suggest that bemcentinib might be useful for the treatment of early SARS-CoV-2 infection for which no medical countermeasures are currently approved, and support testing the efficacy of bemcentinib treatment among hospitalised adults with COVID-19.

The AXL receptor tyrosine kinase promotes the infection of a wide range of enveloped viruses including pox-, retro-, flavi-, arena-, filo-, and alpha-viruses (Shimojima 2006, Brindley 2011, Meertens 2012, Dowall 2016, Meertens 2017). AXL increases viral infection through two mechanisms: 1) enhanced viral entry through “apoptotic mimicry”; and 2) suppression of anti-viral type I IFN responses.

The AXL receptor and related receptors (Tyro3 and MerTK, collectively TAM) are important for the clearance of apoptotic cells (efferocytosis) by macrophages (Lemke 2019). Enveloped viruses co-opt this mechanism to expand tropism and enhance viral entry. GAS6, the AXL ligand, binds PS exposed on the surface of the viral envelope, tethering the viral particle to the AXL receptor and promoting uptake by phagocytosis. This mechanism of viral entry, based on PS exposure, is common to most enveloped viruses and is termed viral “apoptotic mimicry” (Mercer 2008, Bhattacharyya 2013).

Binding of the viral particle to GAS6-AXL potently activates signal transduction through its tyrosine kinase domain to suppress type I IFN signalling and facilitate viral replication (Bhattacharyya 2013, Meertens 2017). AXL expression is induced by inflammation and serves as an innate immune checkpoint. AXL signalling suppresses viral-induced IFN responses via suppressor of cytokine signalling (SOCS)1/3, leading to increased viral replication in infected cells and decreased anti-viral defences of neighbouring cells (Huang 2015, Chen 2018, Strange 2019). Consequently, *Axl*-null mice are resistant to Zika pathogenesis likely due to a combination of reduced virus entry and enhanced IFN responses (Hastings 2019), indicating a potential role for AXL inhibitors as therapeutics during viral infection.

Therapeutic AXL receptor inhibition ameliorated pulmonary pathology resulting from primary viral infection in experimental models, indicating an important role for AXL within the lung (Shibata 2014). During primary respiratory syncytial virus (RSV) infection, AXL inhibition increased the number of IFNg–producing T cells and natural killer (NK) cells, suppressed RSV replication and whole lung levels of interleukin (IL)-4 and IL-13. Also, the lethal effect of intrapulmonary H1N1 infection inflammation was reduced by AXL inhibition. AXL inhibition in infected mice increased the number of IFN-b–producing macrophages and dendritic cells and suppressed neutrophil infiltration.

Bemcentinib is a clinical-stage, oral, selective small molecule AXL kinase inhibitor with well documented anti-viral effects in several systems. Bemcentinib is reported to block dengue, Ebola and Zika virus infections in several cell types including epithelial, fibroblast, endothelial, neuronal and myeloid cell types in in vitro cell culture and organoid systems. Bemcentinib treatment is associated with increased IFN signalling and reduced viral replication (Dowall 2016, Meertens 2017, Strange 2019).

During the 2013/2014 Ebola virus (EBOV) outbreak, bemcentinib was 1 of 60 compounds evaluated by Public Health England as an experimental therapy for EBOV, using its Biosafety Containment Level 4 facilities at Porton Down. Bemcentinib 200 mg/kg/day starting 6 hours post viral challenge protected 1/6 EBOV infected guinea pigs from weight loss and early mortality in an 18-day in vivo mortality study (Dowall 2016), compared with 1/6 untreated animals surviving to Day 18 but exhibiting weight loss during the observation period. The authors concluded that bemcentinib may have had some protective effect in this model.

2.1 Preliminary Efficacy Data vs with SARS-CoV-2 In vitro

Professor Wendy Maury, University of Iowa, conducted a preliminary analysis of the anti-viral effects of bemcentinib on SARS-CoV-2 in a Vero E6 cell line. As shown in Figure 1, bemcentinib incubation starting 1 hour prior to virus inoculation potently inhibited SARS-CoV-2 infection of Vero E6 cells in a dose-dependent manner. Other studies using vesicular stomatitis virus pseudotyped with SARS-CoV spike protein and a mouse betacoronavirus (mouse hepatitis virus [MHV]) showed that bemcentinib may both inhibit uptake and activate the IFN-mediated antiviral gene, ISG15, to control viral infection. SARS-CoV-2 cell tropism is likely to include PS dependent viral uptake and may target critical immune cell populations (eg, macrophages, dendritic cells) that produce IFN and mobilize anti-viral immunity. Importantly, delayed IFN signalling is characteristic of pathogenic human betacoronaviruses and correlates with disease severity in animal models, suggesting that early intervention with IFN-activating treatment may provide therapeutic benefit (Channappanavar 2016). Thus, AXL targeting is expected to attenuate SARS-CoV-2 pathogenesis both by limiting viral uptake and promoting innate anti-viral immunity.

Figure 1 Bemcentinib Potently Inhibits Severe Acute Respiratory Syndrome Coronavirus 2 (SARS-CoV-2) Infection of Vero E6 Cells

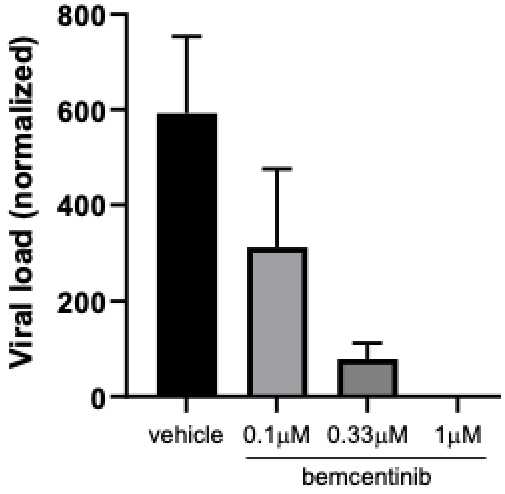

Vero E6 cells (60,000) in a 48-well format were incubated (1 hour) with bemcentinib prior to addition of severe acute respiratory syndrome coronavirus 2 (SARS-CoV-2) (MOI 0.0005). Cells were lysed at 24 hours and viral load was analysed by quantitative reverse transcription polymerase chain reaction (RT-PCR) for spike protein gene expression as normalized to the housekeeping gene *Cyclophilin*. W.Maury *et al*., unpublished results

Importantly, recent data that differentiate SARS-CoV-2 from severe acute respiratory syndrome (SARS) have emerged that support the hypothesis that AXL may have a more dominant role in SARS-CoV-2 infection, and therefore that bemcentinib may have specifically a more important role to play in inhibiting this viral infection:

- SARS-CoV-2 has similar replication kinetics to SARS-CoV, but demonstrates differential sensitivity to type I interferon treatment (One of 2 mechanisms of action of Bemcentinib through AXL inhibition) (Lokugamage 2020).
- Structural analysis of the spike (S) protein of SARS-CoV-2 showed that its S protein has weaker binding to the angiotensin converting enzyme (ACE)2 receptor on human cells compared with strong affinity of human SARS coronavirus (Dong 2020) – supporting the large magnitude effect we have observed for inhibition of viral replication using clinically appropriate doses of bemcentinib in ACE2 receptor +ve cells.

2.2 Dose Justification for Bemcentinib

Bemcentinib's antiviral action stems from a cellular effect, inhibiting AXL kinase to prevent viral attachment and intracellular viral replication (through maximisation of the early type-I interferon rather than a direct antiviral action). From estimates of pooled data, the half maximal inhibitory concentration (IC_{50}) at 24 hours for bemcentinib inhibition of viral load is approximately 140 nM and the approximate concentration required for 90% of maximum inhibition (IC_{90}) is 650 nM. This broadly corresponds to cancer cell data demonstrating high

potency of bemcentinib to inhibit AXL. This high potency is reflected in translational data from clinical trials that demonstrate that at the RP2D steady state, AXL kinase is completely inhibited in myeloblasts from AML patients on bemcentinib (Figure 2).

Clinically, at RP2D, AXL kinase target demonstrates complete kinase inhibition (Figure 2), thus the maximal possible antiviral effect with this mechanism of action is accessed at the current clinical dose.

Clinically, frequent monotherapy complete responses are observed in AML at RP2D (Figure 2). Bemcentinib PK exhibits a prolonged enteral absorptive phase which overlaps with prolonged elimination kinetics after the second dose, to proportionately reduce peak-to-trough difference in plasma concentration-time profile, over a 24-hour dose interval, resulting in smooth steady state without noticeable peak to trough variation (Figure 3). Thus, at the clinical RP2D it is predicted that bemcentinib would be a highly potent antiviral against SARS-CoV-2, and potentially as efficacious as its main use an anticancer therapeutic.

Figure 2 Complete Inhibition of AXL Kinase Activation (p-Axl) in Two Acute Myeloid Leukaemia Patients Treated at the Recommended Phase 2 Dose (200 mg Maintenance Daily Dosing)

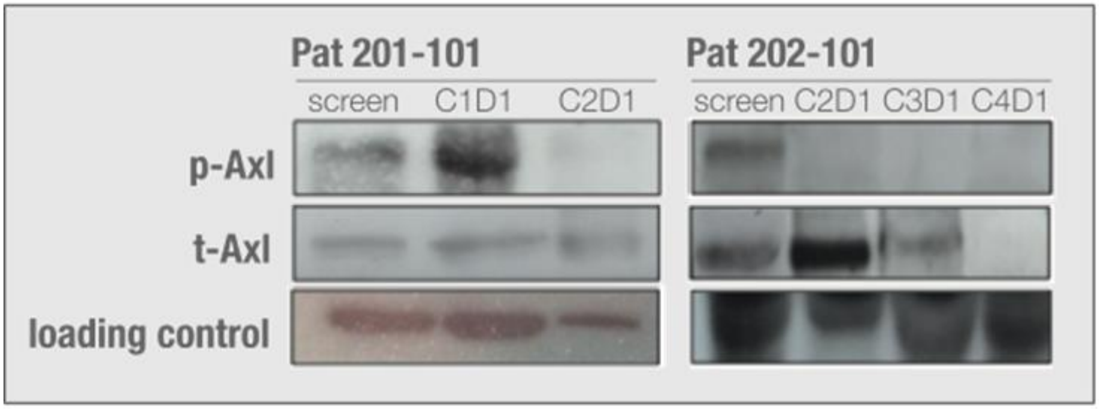

C=cycle; D=day; Pat=patient

Figure 3 Bemcentinib Plasma Pharmacokinetics in Acute Myeloid Leukaemia Patients

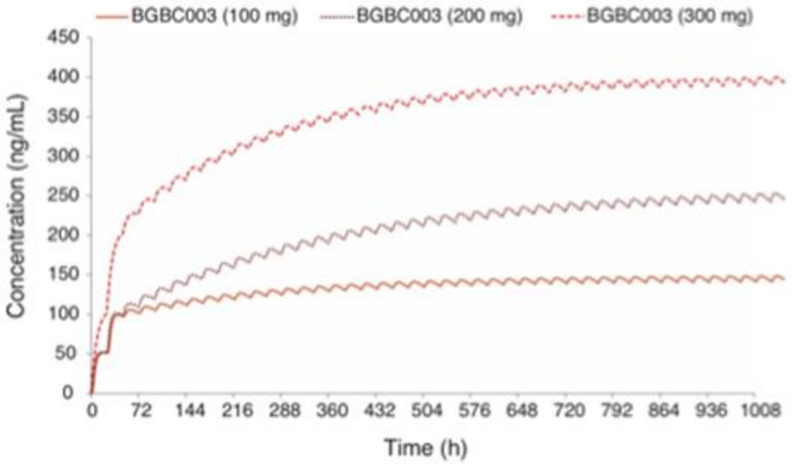

Bemcentinib is given orally as 100-mg capsules. Early clinical development in oncology patients identified the RP2D; dose administration of bemcentinib in this study will utilise the same dosing regimen, ie, a loading dose (400 mg) given once daily for 3 days followed by a maintenance dose of 200 mg once daily. Planned duration of bemcentinib treatment is for a total of 15 days, with the possibility of extension to 21 days for in-patients, at the discretion of the clinical investigator.

In preclinical and Phase 1 clinical studies, it was shown that systemic exposure to bemcentinib increased dose proportionately. The terminal half-life was 45.6 to 88.7 hours in man. Modelling of the PK data from this study indicated that the most effective approach to rapidly achieving steady state is to administer 3 daily loading doses followed by a lower daily maintenance dose.

2.3 Human Experience from Trials in Cancer Patients

Experience has been gained in the use of bemcentinib in the treatment of many types of cancer (including over 286 patients treated with AML, lung cancer, breast cancer, melanoma, and pancreatic cancer) in Phase 2 clinical studies. This has helped to define the safety profile, recommended dose and schedule as a monotherapy (Loges et al 2018). The safety profile of bemcentinib, initially ascertained in normal human volunteers is tolerable and allows monotherapy approaches as well as combinations with chemotherapy (low dose cytosine arabinoside or with docetaxel), targeted therapy (epidermal growth factor receptor inhibitors) and immunotherapy (pembrolizumab). Bemcentinib is given as two oral capsules once daily (200 mg) following three daily 400 mg loading doses, to achieve steady state. Its principal significant AEs are a low incidence of diarrhoea (28% , with 6% Grade 3/4), asymptomatic QT prolongation (6% Grade 3/4), asthenia, and nausea at the RP2D (Loges et al 2018). Many patients have received bemcentinib without untoward effects for over 2 years at full dose. In particular, there were no QTc related cardiac sequelae observed with either monotherapy or combination therapy in over 286 cancer patients, including elderly multimorbid patients (as presented in the Investigator's Brochure). Evidence of monotherapy efficacy has been

documented in heavily pre-treated patients with AML. Complete response rates of approximately 40% have been observed in AXL expressing AML patients (Loges et al 2018), indicating on-target potency and specificity of bemcentinib as a monotherapy, through complete inhibition of the AXL kinase target. Treatment was well tolerated by most subjects, including the frail elderly. Bemcentinib was well tolerated when combined with pembrolizumab, demonstrating synergy with programmed cell death-1 (PD-1) antagonists through targeting of AXL-dependent immune suppressive mechanisms (M2 macrophages, suppressor dendritic cells, regulatory T cells, and myeloid derived suppressor cells). Collectively, bemcentinib mediated activation of innate immunity within the tumour microenvironment synergizes with immune checkpoint therapy (Krebs et al 2019).

3.0 STUDY POPULATION

3.1 Inclusion Criteria

Inclusion criteria 3 and 4 will be modified from the Master Protocol, as the contraception methods and guidance for lactating mothers will need to be for 120 days (not 6 weeks) after termination of study therapy. The revised criteria will be as follows:

3. The patient, and their partner(s), agree to use medically-accepted double-barrier methods of contraception (eg, barrier methods, including male condom, female condom or diaphragm with spermicidal gel) during the study and for at least ***120 days*** after termination of study therapy. A vasectomised partner would be considered an appropriate birth control method provided that the partner is the sole male sexual partner and the absence of sperm has been confirmed. If not, an additional method of contraception should be used. Pre-existing highly effective methods of contraception, such as complete sexual abstinence, intrauterine device, or intrauterine system will be considered acceptable methods of contraception.

   Note: patients admitted on oral contraceptives will not be required to stop hormonal contraceptive but will be required to additionally use a barrier method for 120 days after the last dose of study medication.

   Or,
   Is a woman who is not of childbearing potential (as defined in Appendix 5 of the Master Protocol).

4. Women who are lactating who agree not to breastfeed their child during the study and for at least ***120 days*** after termination of study therapy (they may continue to express milk away from the child during this period, but this milk must be discarded).

3.2 Eligibility Criteria

Overall inclusion and exclusion criteria are presented in Sections 5.1 and 5.2 of the Master Protocol, respectively.

Additional exclusion criteria that are specific to the sub-protocol are as follows:

X1. Inability to swallow capsules (administration via nasogastric tube is permitted)

X2. Current treatment with any agent known to cause QT prolongation. See Appendix 2 for list of relevant medications. The treatment can be discontinued, with sufficient time (5 half-lives) for washout, to allow inclusion of the patient.

X3. Screening 12-lead ECG with a measurable QTc interval according to Fridericia correction (QTcF) >450 ms

X4. Clinically significant hypokalaemia. Individuals who do not meet this criterion may be rescreened once.

X5. Therapeutic anticoagulation with vitamin K antagonists. Note: Patients receiving low doses prescribed to maintain the patency of venous access devices may be included.

X6. Previous bowel resection that would interfere with drug absorption

4.0 STUDY ASSESSMENTS AND PROCEDURES

In addition to the study assessments and procedures described in Section 8.0 of the Master Protocol, assessments specific to the sub-protocol will be performed as described in the following sections. The Schedule of Activities (SoA) for this sub-protocol is presented in Section 1.2.

4.1 Efficacy Assessments

Efficacy assessments will be included as per the Master Protocol. The following endpoints, secondary endpoints in the Master Protocol, will be considered as key secondary endpoints for the purposes of this sub-protocol:

Table 1. Bemcentinib Sub-protocol Specific Key Objectives and Endpoints

Objectives	Endpoints
• To evaluate the ability to prevent deterioration according to the ordinal scale by 1, 2, or 3 points	• The proportion of patients not deteriorating according to the ordinal scale by 1, 2, or 3 points on Days 2, 8, 15, 22, and 29.
• To evaluate the number of oxygen-free days.	• Duration (days) of oxygen use and oxygen-free days.
• To evaluate ventilator-free days and incidence and duration of any form of new ventilation use.	• Duration (days) of ventilation and ventilation-free days.<br>• Incidence of any form of new ventilation use and duration (days) of new ventilation use.
• To evaluate SARS-CoV-2 viral load.	• Qualitative and quantitative polymerase chain reaction (PCR) determination of severe acute respiratory syndrome coronavirus 2 (SARS-CoV-2) in oropharyngeal/nasal swab while hospitalised on Days 1, 3, 5, 8, 11, 15, and (optional) Day 29
• To evaluate SARS-CoV-2 viral load.	• Qualitative and quantitative PCR determination of SARS-CoV-2 in blood and saliva (while hospitalised) on Days 1, 3, 5, 8, 11, 15, and (optional) Day 29 (may be become a secondary endpoint once the assays are available).

Exploratory studies will define the role of PD biomarkers.

Exploratory endpoints relate to the translational plan.

4.2 Safety Assessments

For ECG assessments see the SoA (Section 1.2). Timing of the PK and PD evaluations will be matched to the ECG assessments.

4.2.1 Adverse Events

4.2.1.1 Adverse Events from Indications Under Investigation

Bemcentinib is a relatively safe investigational therapeutic agent. A few patients may experience tiredness and gastrointestinal disturbance (nausea, diarrhoea). Occasionally there may be some changes to blood tests relating to liver function (transaminitis) and some changes on ECG tracing (QT prolongation). These AEs are temporary and lasting only a few days.

4.2.1.2 Adverse Events of Special Interest (AESI)

Not applicable.

4.2.1.3 Disease-related Events and/or Disease-related Outcomes Not Qualifying as Adverse Events or Serious Adverse Events

Not applicable.

4.2.2 Clinical Safety Laboratory Assessments

See Master Protocol for the list of clinical laboratory tests to be performed for this sub-protocol, and the SoA (Section 1.2) for the timing and frequency.

4.3 Pharmacokinetic Assessments

Venous blood samples for PK analysis will be collected according to the SoA (Section 1.2). The samples will be analysed for plasma bemcentinib and additionally for bemcentinib metabolites (to be defined). Detailed procedures for the collection, processing, storage and shipment of the samples will be provided in the Study Laboratory Manual.

Plasma samples for determination of bemcentinib concentration will be analysed by BerGenBio's Bioanalytical Services vendor using the validated liquid chromatography with tandem mass spectrometry method.

4.4 Pharmacodynamic Assessments

Blood samples for PD analysis will be collected according to the SoA (Section 1.2) and stored for future analysis in both the bemcentinib arm and the control arm. The samples will be analysed for soluble AXL, GAS6 (which could be predictive, PD, and mechanism biomarkers and hence require comparison with control group not receiving bemcentinib), and other blood proteins. Detailed procedures for the collection, processing, storage and shipment of the samples will be provided in the Study Laboratory Manual.

This analysis will be in addition to the inflammatory cytokine analysis detailed in the Master Protocol.

5.0 STUDY TREATMENT

5.1 Conclusions and Risk Benefit Statement

Bemcentinib has shown in vitro evidence of antiviral effect against SARS-CoV-2 infection at concentrations below those achieved at the proposed dose regimen for use in this trial. This dose matches the RP2D derived from multiple studies in various cancer populations. Treatment at this dose in 286 patients over a range of 6 weeks up to 2 years demonstrates that monotherapy or combination is largely well tolerated; therefore short term administration (15 to 21 days) in the context of hospitalised patients with SARS-CoV-2 infection is anticipated to be well tolerated. A non-severe, asymptomatic effect on QTc interval has been noted. Regular ECG/continuous ECG monitoring is included in the clinical trial enabling early identification of prolonged QTc and early stopping rules given the high morbidity and potential mortality of SARS-CoV-2 in hospitalised patients, there is a favourable balance of potential benefit: risk in the proposed clinical investigation of bemcentinib treatment for COVID-19 within the context of this clinical trial.

5.2 Bemcentinib Drug Administration

Bemcentinib will be administered as a 400 mg oral loading dose on Days 1, 2, and 3, followed by 200 mg once-daily oral maintenance dose for 15 days with the possibility of extension to 21 days for in patients at the discretion of the clinical investigator.

Bemcentinib should be taken once per day, in the morning after an overnight fast. Tablets should be taken with 100 mL water. Patients should not consume anything other than water for at least 1 hour after taking study drug.

5.3 Dose Modifications and Toxicity Management

Note: this modified guidance has been added to the Investigators' Brochure under relevant section of treatment of COVID-19

Event	Recommended Bemcentinib Dose Modification
Estimated creatinine clearance decreases by more than ≥50% from baseline	Study medication should be withheld until estimated creatinine clearance returns to baseline
ALT or AST increases to >5 ULN	Study medication should be withheld until ALT and AST returns to baseline
**Dose Modification of Bemcentinib Daily Dose for QTc Prolongation**	
**QTcF**	**Recommended Bemcentinib Dose Modification**
**≥501 ms**	
1st occurrence	Discontinue permanently
**Ventricular arrhythmia**	
1st occurrence	Discontinue permanently
ALT=alanine aminotransferase; AST=aspartate aminotransferase; QTcF= QTc interval according to Fridericia correction; ULN=upper limit of normal<br>Notes:<br>Serum calcium, magnesium and potassium should be measured regularly whilst receiving bemcentinib; all abnormal results should be corrected; check for use of concomitant medication that are associated with QT prolongation.	

5.4 Prohibited Concomitant Medications

Administration of bemcentinib is contraindicated in patients requiring treatment with concomitant medications known to prolong QTc interval and promote Torsade de Points (TdP) listed in Appendix 2. Patients already in receipt of such medications should be excluded unless medications are discontinued and a sufficient wash out period is allowed prior to starting bemcentinib.

Medicines with both cytochrome P450 (CYP) 3A4 and TdP liabilities are particularly hazardous and their concomitant use is a reason for exclusion of patients.

Concomitant medications that are CYP3A4 substrates are not reasons for exclusion of patients, however they should be discontinued or used with caution.

Treatment with histamine receptor 2 inhibitors (cimetidine, ranitidine) or protein pump inhibitors (omeprazole) is permitted provided that administration is in the evening.

5.5 Study Drug Information

5.5.1 Study Medication

The drug product is presented in a single strength: 100 mg bemcentinib in size 0, Swedish Orange hydroxypropyl methylcellulose capsules for oral administration. Bemcentinib capsules

consists of a wet granulated blend of 40% drug substance with standard excipients (lactose monohydrate, microcrystalline cellulose, crospovidone, polyvinylpyrrolidone, colloidal silicon dioxide, and magnesium stearate). Bemcentinib has been manufactured according to appropriate Good Manufacturing Practice standards.

Bemcentinib capsules are packaged in 50-mL white opaque round high density polyethylene bottles containing 22 capsules. The bottles are closed with opaque 32-mm child-resistant screw caps and sealed with tamper-resistant tape.

Refer to the current version of the Bemcentinib Investigational Medicinal Product Dossier/Investigator's Brochure for additional information on the physical, chemical and pharmaceutical properties of bemcentinib.

5.5.2 Bemcentinib Storage, Dispensing, and Destruction

Bemcentinib will be shipped to the participating site by PCI Pharma Services, UK, and must be stored at the site in a secure location under ambient temperature conditions (<25°C).

Accountability for study treatment is the responsibility of the Investigator. The Investigator/designee must ensure that the bemcentinib will only be dispensed to patients in accordance with the dosing instructions in this protocol. Study staff should refer to the Bemcentinib Pharmacy Manual for specific instructions regarding the handling, storage, dispensing and destruction of bemcentinib.

6.0 REFERENCES

Bhattacharyya S, Zagórska A, Lew ED, et al. Enveloped viruses disable innate immune responses in dendritic cells by direct activation of TAM receptors. Cell Host Microbe 2013;14:136-47.

Brindley MA, Hunt CL, Kondratowicz AS, et al. Tyrosine kinase receptor Axl enhances entry of Zaire ebolavirus without direct interactions with the viral glycoprotein. Virology 2011;415:83-94.

Channappanavar R, Fehr AR, Vijay R, et al. Dysregulated Type I Interferon and Inflammatory Monocyte-Macrophage Responses Cause Lethal Pneumonia in SARS-CoV-Infected Mice. Cell 2016;19:181-93.

Chen J, Yang YF, Yang Y, et al. AXL promotes Zika virus infection in astrocytes by antagonizing type I interferon signalling. Nat Microbiol 2018;3(3):302-9.

Dong N, Yang X, Ye L, Chen K, Chan EW, Chen S. Genomic and protein structure modelling analysis depicts the origin and pathogenicity of 2019-nCoV, a new coronavirus which caused a pneumonia outbreak in Wuhan, China [version 2; peer review: 1 not approved]. F1000Research 2020, 9:121 (https://doi.org/10.12688/f1000research.22357.2)

Dowall SD, Bewley K, Watson RJ, et al. Antiviral Screening of Multiple Compounds against Ebola Virus. Viruses 2016;8(11). pii:E277.

Hastings AK, Hastings K, Uraki R, et al. Loss of the TAM Receptor Axl Ameliorates Severe Zika Virus Pathogenesis and Reduces Apoptosis in Microglia. iScience 2019;13:339-50.

Huang MT, Liu WL, Lu CW, et al. Feedback regulation of IFN-αβ signalling by Axl receptor tyrosine kinase modulates HBV immunity. Eur. J. Immunol. 2015;45:1696-705.

Krebs M et al. A phase II study of bemcentinib (BGB324), a first-in- class selective AXL inhibitor, in combination with pembrolizumab in patients with advanced NSCLC: Updated analysis. SITC 2019, oral presentation in High Impact Clinical Trials session.

Lemke G. How macrophages deal with death. Nat Rev Immunol. 2019;19(9):539-49.

Loges S et al. Comprehensive Analysis of the Dose Escalation, Expansion and Correlates in the Ph I/II Trial BGBC003 with the Selective Oral AXL Inhibitor Bemcentinib (BGB324) in Relapsed/Refractory AML and MDS ASH 2018, presentation.

Lokugamage KG, Hage A, Schindewolf C, Rajsbaum R, Menachery VD. SARS-CoV-2 is sensitive to type I interferon pretreatment. bioRxiv preprint doi: https://doi.org/10.1101/2020.03.07.982264.Meertens L, Carnec X, Lecoin MP, et al. The TIM

and TAM Families of Phosphatidylserine Receptors Mediate Dengue Virus Entry. Cell Host Microbe 2012;12(4):544-57.

Meertens L, Labeau A, Dejarnac O, et al. Axl mediates ZIKA virus entry in human glial cells and modulates innate immune responses. Cell Rep 2017;18(2):324-33.

Mercer J, Helenius A. Vaccinia virus uses macropinocytosis and apoptotic mimicry to enter host cells. Science 2008;320(5875):531-5.

Shibata T, Habiel DM, Coelho AL, Kunkel SL, Lukacs NW, Hogaboam CM. Axl Receptor Blockade Ameliorates Pulmonary Pathology Resulting from Primary Viral Infection and Viral Exacerbation of Asthma. J Immunol. 2014;192(8):3569-81.

Shimojima M, Takada A, Ebihara H, et al. Tyro3 family-mediated cell entry of Ebola and Marburg viruses. J Virol. 2006;80(20):10109-16.

Strange DP, Jiyarom B, Pourhabibi Zarandi N, et al. Axl promotes Zika virus entry and modulates the antiviral state of human Sertoli cells. mBio 2019;10(4):e01372-19.

7.0 AMENDMENT HISTORY

Protocol amendment 01 (dated 22 April 2020) replaces the original sub-protocol for bemcentinib (dated 20 April 2020).

The amendment incorporates the following main changes:

- A change of Sponsor, from BerGenBio ASA to University Hospital Southampton NHS Foundation Trust, with a corresponding change to the named Sponsor signatory.
- Addition of exclusion criterion for clinically significant hypokalaemia.
- Clarification that for the exclusion criterion of inability to swallow capsules, administration via nasogastric tube is permitted.
- Clarification that the physical examination at screening includes height and weight (this is part of the Master Protocol).
- Addition of samples for cytokine analysis and PBMC phenotyping on the Schedule of Activities (this is part of the Master Protocol).
- Removal of specific blood volumes from the Schedule of Activities.
- Clarified that analysis of inflammatory cytokines is part of the Master Protocol.
- Clarification regarding known adverse events for bemcentinib.

8.0 APPENDICES

Appendix 1 Abbreviations

Abbreviation	Definition
ACE	Angiotensin-converting enzyme
AE	Adverse event
AML	Acute myeloid leukaemia
BP	Blood pressure
CYP	Cytochrome P450
EBOV	Ebola virus
ECG	Electrocardiogram
GAS6	Growth arrest-specific 6
IC_{50}	Half maximal inhibitory concentration
IC_{90}	Concentration required for 90% of maximum inhibition
IFN	Interferon
IL	Interleukin
MHV	Mouse hepatitis virus
NK	Natural killer
NYHA	New York Heart Association
PD	Pharmacodynamic
PD-1	Programmed cell death-1
PK	Pharmacokinetic
PS	Phosphatidylserine
QTcF	QTc interval according to Fridericia correction
RP2D	Recommended Phase 2 dose
RSV	Respiratory syncytial virus
SARS	Severe acute respiratory syndrome
SARS-CoV-2	Severe acute respiratory syndrome coronavirus 2
SoA	Schedule of Activities
SOCS	Suppressor of cytokine signalling
TAM	Tyro3, AXL, MerTK
TdP	Torsade de Points

Appendix 2 Prohibited Concomitant Medication Due to QT Prolongation Risk or CYP3A4 Interaction with Narrow Therapeutic Index

For any concomitant medication, please check the following website for the drug's *Torsades de Pointes* (TdP) risk: https://crediblemeds.org/oncosupport/.

Drugs with known (TdP) risk are reason for EXCLUSION. For drugs with a conditional risk, please review the product label and correct any abnormalities, eg, hypokalaemia.

**Common medication Associated with a Risk of QT Prolongation and TdP –**<br>**USE PROHIBITED AS CONCOMITANT MEDICATION**		
**t½ less than 6 hours**	**t½ between 6 – 12 hours**	**t½ greater than 12 hours**
Azithromycin 2-4 hours[$]	Cisapride 10 hours **	Amiodarone 50 days[$]
Clarithromycin 3-4 hours[$]	Disopyramide 6.7 hours[$]	Astemizole 24 hours **
Cocaine 0.6 – 1.3 hours[$]	Ketoconazole 3 – 10 hours[$]	Chloroquine 1-2 months[$]
Droperidol 2 hours[$]	Moxifloxacin 12 hours[$]	Citalopram 35 hours[$]
Erythromycin 2 hours[$]	Quinidine 6 hours **[$]	Escitalopram 30 hours[$]
Ondansetron 3 hours[$]	Voriconazole 6 hours[$]	Fluconazole 30 hours[$]
Procainamide 2.5-4.75 hours[$]		Haloperidol 15 – 27 hours[$]
Terfenadine 3.5 hours**		Methadone 25 – 55 hours[$]
		Petamidine 10 – 14 days[$]
		Pimozide 55 hours **
		Sotalol 10 – 20 hours[$]
		Thioridazine 21 – 24 hours[$]
***also CYP3A4 substrates*	[$] also TdP risk	
**Sensitive CYP3A4 Substrates With A Narrow Therapeutic Margin**<br>THESE MEDICATIONS SHOULD BE **DISCONTINUED** BEFORE ENROLMENT		
**t½ less than 6 hours**	**t½ between 6 – 12 hours**	**t½ greater than 12 hours**
alfentanyl 90-111 minutes	astemizole 7 – 9 hours	pimozide 55 hours
dihydroergotamine & ergotamine 2 hours	cisapride 12 hours	sirolimus 63 hours
Fluticasone 3 – 8hours	cyclosporine 8.4 hours	
Terfenadine 3.5 hours	Fentanyl 8 – 10 hours	
	quinidine 6 hours	
	tacrolimus (FK506) 12 hours	

Woosley RL, Heise CW , Gallo T, Tate J, Woosley D and Romero KA, www.CredibleMeds.org, QTdrugs List, [14Apr2020], AZCERT, Inc. 1822 Innovation Park Dr., Oro Valley, AZ 85755

Appendix 3 Signature of Investigator

PROTOCOL TITLE: A Multicentre, Seamless, Phase 2 Adaptive Randomisation Platform Study to Assess the Efficacy and Safety of Multiple Candidate Agents for the Treatment of COVID-19 in Hospitalised Patients

SUB-PROTOCOL NO: ACCORD-2-002

SUB-PROTOCOL FOR CANDIDATE AGENT BEMCENTINIB

VERSION: Amendment 02

This sub-protocol is a confidential communication of the Sponsor. I confirm that I have read this sub-protocol, I understand it, and I will work according to this sub-protocol, in conjunction with the Master Protocol for the overall platform study. I will also work consistently with the ethical principles that have their origin in the Declaration of Helsinki and that are consistent with Good Clinical Practices and the applicable laws and regulations. Acceptance of this document constitutes my agreement that no unpublished information contained herein will be published or disclosed without prior written approval from the Sponsor.

Instructions to the Investigator: Please SIGN and DATE this signature page. PRINT your name, title, and the name of the study centre in which the study will be conducted. Return the signed copy to the Contract Research Organization/Sponsor.

I have read this sub-protocol in its entirety and agree to conduct this part of the study accordingly:

Signature of Investigator: ____________________________ Date: ________

Printed Name: ____________________________

Investigator Title: ____________________________

Name/Address of Centre: ____________________________

____

____
