# Supplementary material for: ACCORD: A Multicentre, Seamless, Phase 2 Adaptive Randomisation Platform Study to Assess the Efficacy and Safety of Multiple Candidate Agents for the Treatment of COVID-19 in Hospitalised Patients: A structured summary of a study protocol for a randomised controlled trial
Source: Trials. 2020 Jul 31;21:691. doi: 10.1186/s13063-020-04584-9 (PMC7393340; doi:10.1186/s13063-020-04584-9)
Supplement: Supplementary file 1 — Additional file 1. [file 13063_2020_4584_MOESM1_ESM.zip › ACCORD-2-004 Acalabrutinib_SubprotocolR0.pdf]

TITLE PAGE

Master Protocol Title: ACCORD-2: A Multicentre, Seamless, Phase 2 Adaptive Randomisation Platform Study to Assess the Efficacy and Safety of Multiple Candidate Agents for the Treatment of COVID-19 in Hospitalised Patients

Sub-protocol Number: ACCORD-2-004

Subprotocol for Candidate Agent Acalabrutinib

Agent Owner Identifier: D822FC00002

Study Phase: 2

Sponsor Name: University Hospital Southampton NHS Foundation Trust

Legal Registered Address: **Southampton General Hospital**
Level E, Laboratory & Pathology Block, SCBR - MP138
Tremona Road
Southampton SO16 6YD, UK

Regulatory Agency Identifying Number(s): EudraCT 2020-001736-95

IRAS Number: **282769**

RHM Number: **MED1711**

Date of Sub-protocol: 01 May 2020

Version: Protocol Amendment 01, Final

Chief Investigator Signatory:

I have read this sub-protocol in its entirety and agree to conduct this part of the study accordingly:

S. D. Singh 1st MAY 2020

Professor Dave Singh MD, FERS, FBPhS
Professor of Clinical Pharmacology and Respiratory
Medicine & Honorary Respiratory Consultant

Date

TABLE OF CONTENTS

TABLE OF TABLES 4

PROTOCOL AMENDMENT HISTORY 5

1.0 SUB-PROTOCOL SUMMARY 6
1.1 Overview of Sub-protocol 6
1.2 Schedule of Activities 7

2.0 BACKGROUND/RATIONALE IN SUPPORT OF ACALABRUTINIB IN COVID-19 11
2.1 Dose Justification for Acalabrutinib 12

3.0 STUDY POPULATION 13
3.1 Enrolment and Screening 13
3.2 Eligibility Criteria 13
3.2.1 Inclusion Criteria 13
3.2.2 Exclusion Criteria 13

4.0 STUDY ASSESSMENTS AND PROCEDURES 14
4.1 Safety Assessments 14
4.1.1 Adverse Events 14
4.1.2 Clinical Safety Laboratory Assessments 14
4.2 Pharmacokinetic Assessments 14
4.3 Pharmacodynamic Assessments 15

5.0 STUDY TREATMENT 16
5.1 Treatment Plan 16
5.2 Study Drug Administration 16
5.3 Dose Modifications and Toxicity Management 16
5.4 Concomitant Medications 17
5.4.1 Premedications 17
5.4.2 Prohibited or Restricted Concomitant Therapy 17
5.4.3 Acalabrutinib Drug-drug Interaction Guidance in the Presence of Life-threatening COVID-19 Infection 18
5.4.4 Use in Patients with Renal Impairment 21
5.4.5 Use in Patients with Hepatic Impairment 21
5.5 Study Drug Information 22

6.0 REFERENCES....23

7.0 APPENDICES....25
Appendix 1 Abbreviations....26
Appendix 2 Examples of Coadministered Drugs That Need Additional Consideration....27
Appendix 3 Contraceptive Guidance....29
Appendix 4 Signature of Investigator....31

TABLE OF TABLES

Table 1 Guidance on Acalabrutinib Use with CYP3A Inhibitors, CYP3A Inducers and agents that reduce Gastric Acid production and pH....19
Table 2 Preliminary Experience Obtained During Clinical Studies of Acalabrutinib Co-Administration with Strong CYP3A Inhibitors....20
Table 3 CYP3A Inhibitors....27
Table 4 CYP3A Inducers....28
Table 5 Other Drugs Needing Additional Considerations....28

PROTOCOL AMENDMENT HISTORY

Protocol Amendment 01 (dated 01 May 2020) replaces the original sub-protocol for acalabrutinib (dated 29 April 2020).

The amendment incorporates the following main changes:

- Additional text regarding management of adverse events.
- Clarified that patients should only be discharged at least 24 hours after the last dose and review of safety laboratory results.

1.0 SUB-PROTOCOL SUMMARY

1.1 Overview of Sub-protocol

In addition to the Master Protocol, this sub-protocol contains the following information specific to the candidate agent acalabrutinib:

- Dosing schedule and Schedule of Activities (SoA; Section 1.2)
- Background on the candidate agent and dose justification (Section 2.0)
- Sub-protocol-specific exclusion criteria (Section 3.2.2)
- Potential risks and adverse events of special interest (AESIs) for acalabrutinib (Section 4.1.1.2)
- Pharmacokinetic (PK) sample assessments (Section 1.2 and Section 4.2).
- Pharmacodynamic (PD) sample assessments (Section 1.2 and Section 4.3)
- Concomitant medications specific to candidate agent (Section 5.4)
- Formulation, dose, and route of administration for acalabrutinib (Section 5.5)

1.2 Schedule of Activities

	**Screening**	**Baseline**					
**Day (± Window)**	**Day -1 or Day 1**	**Day 1**	**Daily Until Hospital Discharge**	**Day 15[a] (±2 days)**	**Day 29[a] (±3 days)**	**Day 60 (±4 days) (Follow-up)**	**Day 90 (±6 days) (End of Study)**
ELIGIBILITY							
Informed Consent	X						
Demographics	X						
Relevant medical history[b]	X						
Review SARS-CoV-2 diagnostic tests	X						
Inclusion and exclusion criteria	X						
12-lead Electrocardiogram	X						
STUDY INTERVENTION							
Randomisation		X					
Administration of acalabrutinib (twice daily, every 12 ± 3 hours)		X	Days 2-10 while hospitalised[c]				
Treatment with SoC		X	X				
STUDY PROCEDURES							
Clinical frailty score	X						
Diagnostic imaging (X-ray and/or computed tomography)	X						
Physical examination (including presenting symptoms, height, weight)	X						
Targeted physical examination (focused on lung auscultation)			X				
Vital signs, including temperature, pulse rate, blood pressure, respiratory rate, SpO_2		X[d]	X	X	X		
Clinical assessments[e]		X[d]	X	X	X		
Targeted medication review (including use of vasopressors)		X[d]	X	X	X		
Adverse event evaluation		X	X	X	X	X	X

	Screening	Baseline					
**Day (± Window)**	**Day -1 or Day 1**	**Day 1**	**Daily Until Hospital Discharge**	**Day 15[a] (±2 days)**	**Day 29[a] (±3 days)**	**Day 60 (±4 days) (Follow-up)**	**Day 90 (±6 days) (End of Study)**
Disease-related co-infection evaluation (including microbiologic/infectious agent assessment/results; bacteria, viral, fungi)		X	X				
Survival status		X	X	X	X	X	X
Blood gases and FiO_2 at worst PO_2[f]	X	X	X	X			
SAFETY LABORATORY							
Haematology, chemistry, liver function tests, coagulation[g]	X[h]	X[d,i]	Days 3, 5, 8, 11 (all ±1 day) while hospitalised				
NT-proBNP	X[h]	X[d,i]	Days 3, 5, 8, 11 (all ±1 day) while hospitalised				
Pregnancy test for females of childbearing potential	X[h]						X
RESEARCH LABORATORY							
Blood (SST) for exploratory inflammatory cytokine analysis		X	Days 3, 5, and 8	X	X		
Blood (sodium heparin tube) for PBMC phenotyping[j]		X	Days 3, 5, and 8	X	X		
Blood (EDTA) for SARS-CoV-2 PCR (qualitative and quantitative)		X	Days 3, 5, 8, 11 (all ±1 day) while hospitalised	X	X		
Oropharyngeal/nasal swab for SARS-CoV-2 PCR (qualitative and quantitative)		X	Days 3, 5, 8, 11 (all ±1 day) while hospitalised	X	X		

	**Screening**	**Baseline**					
**Day (± Window)**	**Day -1 or Day 1**	**Day 1**	**Daily Until Hospital Discharge**	**Day 15[a] (±2 days)**	**Day 29[a] (±3 days)**	**Day 60 (±4 days) (Follow-up)**	**Day 90 (±6 days) (End of Study)**
Saliva for SARS CoV-2 PCR (qualitative and quantitative)		X	Days 3, 5, 8, 11 (all ±1 day) while hospitalised	X	X		
Blood (SST) for SARS-CoV-2 serology research (host response)		X	Day 8	X	X	X	
Blood sample (PAXGENE) for transcriptome analysis (host genome)[k]		X	Day 8	X			
Blood (EDTA) host genome (host DNA)[k]		X					
Mid-turbinate nasal swab viral genome[k]		X					
Blood for acalabrutinib pharmacodynamics		X[l]	Day 3[m]				
Blood for acalabrutinib pharmacokinetics			Day 3[n]				

EDTA=ethylenediaminetetraacetic acid; FiO2=fraction of inspired oxygen; NT-proBNP=N terminal pro hormone B type natriuretic peptide; PBMC=peripheral blood mononuclear cell; PCR=polymerase chain reaction; PO_2=partial pressure of oxygen; SARS CoV-2=severe acute respiratory syndrome coronavirus 2; SoC=standard of care; SpO2=oxygen saturation; SST = serum separator tube.

[a] These visits will be performed even if a patient has already been discharged. If discharged prior to scheduled visit, in person visits are preferred, but recognising that quarantine and other factors may limit the patient's ability to return to the study centre, these visits may be conducted by telephone or with a home visit by study staff. For visits conducted by telephone, it will not be possible to perform some scheduled assessments (eg, vital signs). The Day 29 assessments will also be performed, where possible, for patients who discontinue the study prematurely.

[b] Medical history includes estimated date and time of first symptoms and number of co morbidities (eg, respiratory, cardiovascular, metabolic, malignancy, endocrine, gastrointestinal, immunologic, renal).

[c] See Section 5.2 of this sub-protocol and Section 7.1 of the Master Protocol regarding circumstances requiring early discontinuation of acalabrutinib.

[d] Baseline assessments should be performed prior to study drug administration.

[e] Includes ordinal score, National Early Warning Score 2 (NEWS2), oxygen requirement, noninvasive or invasive ventilator requirement, including start and stop of low or high flow oxygen supply or of any form of ventilation etc.

[f] If done as part of SoC, blood gases results to be fully recorded with date and time.

[g] For parameters, see Table 5 of the Master Protocol.

[h] Laboratory tests performed in the 48 hours prior to enrolment will be accepted for determination of eligibility.

[i] Any laboratory tests performed as part of routine clinical care within the specified visit window can be used for safety laboratory testing.

[j] Samples collected for immediate laboratory processing and frozen storage.

[k] Samples collected dependent on capacity of study centre, need for reduced study burden on staff, and potentially limited access to patients.

[l] Whole blood will be collected predose and 4 hours postdose on Day 1 for PBMCs.
[m] Whole blood will be collected predose on Day 3 for PBMCs.
[n] Predose, 0.5, 1, 2, 4, and 6 hours post dose on Day 3.

2.0 BACKGROUND/RATIONALE IN SUPPORT OF ACALABRUTINIB IN COVID-19

Coronavirus disease 2019 (COVID-19) is a new pandemic disease caused by the severe acute respiratory syndrome coronavirus 2 (SARS-CoV-2). In a retrospective study of 191 patients with COVID-19, sepsis was the most frequently observed complication, followed by respiratory failure, acute respiratory distress syndrome (ARDS), heart failure, and septic shock. While sepsis might be directly caused by SARS-CoV-2 infection, further research is needed to investigate the pathogenesis of COVID-19 illness. Most COVID-19 cases (~80%) are mild respiratory illnesses; 5% to 15% require hospitalisation (mostly due to pneumonia) and can progress quickly to severe acute lung injury and ARDS,[1,2,3] which is associated with high mortality.

Normally, human coronaviruses are detected and cleared by the immune system, but a subset of patients experience increased severity of symptoms.[4,3] These symptoms have been associated with the loss of control of the virally induced immune response .[5] In these cases, the inflammatory response is hypothesised to be a major pathogenic mechanism of ARDS through modulation of pulmonary macrophages and dendritic cells [6,7,8,9] and/or neutrophils.[10] Putative inflammatory mediators include interleukin (IL)-1β, IL-6, IL-8, IL-10, tumour necrosis factor alpha (TNFα), and monocyte chemoattractant protein-1 (MCP-1).[11,10,9] During the acute phase of coronavirus infection, T cells are the critical mediators of clearance of infection, while B cells generate a protective humoral response[12,13]. Generation of antibodies against coronaviruses is not always protective. In a mouse model, anti-spike immunoglobulins (specific to SARS-COV) could skew the inflammation-resolving response which lead to severe acute lung injury in mice.[14]

Bruton's tyrosine kinase (Btk) is a Tec family non-receptor protein kinase, expressed in B cells, myeloid cells, osteoclasts, mast cells and platelets. The function of Btk in signalling pathways activated by the engagement of the B-cell receptor has been well established.[15] Btk is also involved in the following biologic processes: Fc gamma receptor signalling in myeloid cells, mast cell degranulation, and signalling through Toll-like receptors (TLRs) in macrophages and neutrophils. Specifically, Btk is required for TLR 7/8 signalling, which recognise single strand RNA viruses such as coronaviruses, signal through Btk in macrophages.[16]

Recently, Btk inhibition has been shown to rescue mice from lethal influenza A virus-induced acute lung injury by significantly decreasing lung inflammation and macrophage/monocyte mediated cytokines/chemokines (TNFα, IL-1β, IL-6, MCP-1, etc) in the lung homogenates. These results suggest that Btk inhibition may represent a new immunomodulatory treatment for virally induced lung damage driven by excessive inflammation.[17] Additionally, in a murine model of sepsis, acalabrutinib has been shown to ameliorate cardiac dysfunction by suppressing pro-inflammatory cytokines/chemokines associated with sepsis.[18] Patients with haematologic malignancies treated with acalabrutinib have shown significant reduction of several

cytokines/chemokines including pro-inflammatory markers such as: TNFα ($p < 0.001$), IL-10 ($p < 0.001$), MCP-1 ($p < 0.01$), MIP-1beta ($p < 0.001$), MIP-1 alpha ($p < 0.001$), IL-16 ($p < 0.001$), thymus- and activation-regulated chemokine (TARC; $p < 0.001$), CXCL13 ($p < 0.001$), Granzyme A ($p < 0.001$), [19,20] and IL-6 ($p < 0.05$) (data on file). Several of these cytokines/chemokines have been shown to be associated with more severe illness in COVID-19 patients. We hypothesise that acalabrutinib treatment will inhibit cells that produce pro-inflammatory cytokines/chemokines, will lead to reduced inflammation of the lungs in patients with COVID-19, and mitigate the pathophysiologic response that leads to the most severe morbidity and mortality associated with viral infection.

Together, strong scientific evidence justifies a clinical study in this patient population. The purpose of this Phase 2 study is to evaluate the preliminary efficacy and safety of adding acalabrutinib to standard of care (SoC) for patients hospitalised or admitted to the intensive care unit due to COVID-19 infection.

2.1 Dose Justification for Acalabrutinib

Acalabrutinib 100 mg twice daily (bid) has been evaluated in various indications (ie, B-cell malignancies and solid tumours) alone and in combination with anti-CD20 antibodies, chemotherapy, a phosphatidylinositol-3-kinase (PI3K) inhibitor, and an anti-programmed cell death 1 antibody. No dose-limiting toxicities have been identified for acalabrutinib alone or when given in combination with these agents. For all these indications, acalabrutinib was administered daily until disease progression; some patients have been receiving acalabrutinib for > 5 years. The long-term safety experience of chronic administration of acalabrutinib 100 mg bid monotherapy and in combination with other agents, supports the proposed dosage of acalabrutinib 100 mg bid for acute treatment. In addition, correlative studies—in patients with chronic lymphocytic leukaemia treated with acalabrutinib 200 mg once daily (qd) or 100 mg bid—show bid dosing maintained higher Btk occupancy and achieved more potent nuclear factor (NF)-kappaB pathway inhibition compared with qd dosing .[21] Activation of NF-kappaB occurs in the lungs of patients with ARDS and may contribute to the increased expression of proinflammatory mediators.[22] Therefore, bid dosing is proposed for this study to ensure maximum target engagement.

3.0 STUDY POPULATION

3.1 Enrolment and Screening

Enrolment, screening, and first dose of acalabrutinib must take place within 24 hours of admission to the hospital.

3.2 Eligibility Criteria

Overall inclusion and exclusion criteria are presented in Sections 5.1 and 5.2 of the Master Protocol, respectively. The following sections detail variations to those criteria that are specific to this sub-protocol.

3.2.1 Inclusion Criteria

Inclusion criterion 3 from the Master Protocol, related to contraception, will be replaced with the following:

3. a) Male subjects:

- A male subject must agree to use contraception as detailed in Appendix 3 of this protocol during the treatment period and for at least 12 weeks after the last dose of study treatment and refrain from donating sperm during this period.

b) Female subjects:

- A female subject is eligible to participate if she is not pregnant (see Appendix 3), not breastfeeding, and at least 1 of the following conditions applies:

  i) Not a woman of childbearing potential (WOCBP) as defined in Appendix 3.
  OR
  ii) A WOCBP who agrees to follow the contraceptive guidance in Appendix 3 during the treatment period and for at least 12 weeks after the last dose of study treatment.

3.2.2 Exclusion Criteria

Additional exclusion criteria that are specific to the sub-protocol are as follows:

X1. Unable to take acalabrutinib by mouth

X2. Profound neutropenia and/or profound thrombocytopenia as defined as an absolute neutrophil count < 500/µL and platelet count < 50,000/µL respectively at screening per local laboratory

4.0 STUDY ASSESSMENTS AND PROCEDURES

In addition to the study assessments and procedures described in Section 8.0 of the Master Protocol, assessments specific to the sub-protocol will be performed as described in the following sections. The SoA for this sub-protocol is presented in Section 1.2.

4.1 Safety Assessments

4.1.1 Adverse Events

Refer to Section 8.4 and Appendix 4 in the Master Protocol for information on definitions and procedures for recording, evaluating, follow-up, and reporting of adverse events (AEs) and serious adverse events (SAEs).

All AEs should be managed according to physician judgement and applicable local/national guidelines. Section 7.1 of the Master Protocol describes the criteria (including AEs) where a patient should be withdrawn from study treatment.

4.1.1.1 Adverse Events from Approved Indications

Adverse events (AEs) are known to be associated with acalabrutinib are listed below

a) **Occurring in more than 1 in 10 people:**

Infections, headache, diarrhoea, bruising and increased tendency to bruise, bleeding, musculoskeletal and joint pain, nausea, fatigue, skin rashes, leukopaenia, constipation, anaemia, dizziness, vomiting, abdominal pain

b) **Occurring in more than 1 in 100 people:**

Thrombocytopenia, epistaxis, asthenia, atrial fibrillation, atrial flutter

4.1.1.2 Adverse Events of Special Interest

The following are defined as AESIs for acalabrutinib in this protocol:

- Ventricular arrhythmias including ventricular tachycardia, ventricular fibrillation and frequent ectopic ventricular systoles, eg, bigeminy and trigeminy.

4.1.2 Clinical Safety Laboratory Assessments

See Table 5 in the Master Protocol for the list of clinical laboratory tests to be performed for this sub-protocol, and to the SoA (Section 1.2) for the timing and frequency.

4.2 Pharmacokinetic Assessments

Blood samples for PK analysis will be collected as specified in the SoA (Section 1.2). The samples will be analysed for acalabrutinib and its active metabolite, ACP-5862. Sample handling

will be provided in laboratory manual. The exact time of acalabrutinib dose, dosing route, and exact time of PK collection must be recorded.

4.3 Pharmacodynamic Assessments

Blood samples for PD analysis will be collected as specified in the SoA (Section 1.2). These samples will be used to measure Btk occupancy in peripheral blood mononuclear cells (PBMCs) as a measure of PD marker for acalabrutinib.

5.0 STUDY TREATMENT

5.1 Treatment Plan

See SoA (Section 1.2) and Section 5.2.

5.2 Study Drug Administration

Patients will take acalabrutinib 100-mg capsules bid by mouth. Capsules will be taken with 8 ounces (approximately 240 mL) of water. The capsules should be swallowed intact and patients should not attempt to open capsules or dissolve them in water. Acalabrutinib can be taken with or without food.

If vomiting occurs after taking acalabrutinib, the patient should not retake acalabrutinib until the next scheduled dose.

Acalabrutinib treatment should begin on Day 1 and continue until Day 10 as per the SoA (Section 1.2). Patients should only be discharged at least 24 hours after the last dose and review of safety laboratory results. If a patient recovers sufficiently to be discharged before Day 11 (or is expected to) then acalabrutinib treatment should be discontinued early, so that the 24-hour minimum period between last dose and discharge is maintained. Patients who are unable to take acalabrutinib by mouth for more than 24 hours must be permanently discontinued.

5.3 Dose Modifications and Toxicity Management

In general, if a patient experiences a Common Terminology Criteria for Adverse Events Grade 1 or Grade 2 AE (see Appendix 4 of the Master Protocol for definition of these grades), no dose modification is required. Acalabrutinib therapy should be modified for the following AEs:

Event	Acalabrutinib Dose Modification
Grade 4 neutrophil count decrease (absolute neutrophil count [ANC] < 500/µL)	• Hold acalabrutinib and introduce granulocyte colony-stimulating factor (G-CSF); continue to monitor ANC.<br>• Acalabrutinib should only be resumed once neutropenia has resolved to Grade 1 or baseline. If this has not occurred within 3 days of adverse event onset, discontinue acalabrutinib.
Presence of significant bleeding with or without thrombocytopenia	Discontinue acalabrutinib
Grade 4 platelet count decreases if it does not improve to Grade ≤2 with platelet transfusion	Discontinue acalabrutinib
Grade 3 or 4 nausea, vomiting, or diarrhoea, if persistent despite optimal antiemetic and/or antidiarrheal therapy	Discontinue acalabrutinib
Any other unmanageable Grade 3 or Grade 4 toxicity	Discontinue acalabrutinib

Clinical judgment should be used to determine appropriate management of the patient during any AE.

Acalabrutinib may be held for a maximum of 3 consecutive days from expected dose due to toxicity. Any other clinically important events where dose delays may be considered appropriate by the Investigator, as well as continuation of therapy beyond 3 days, must be discussed with the Medical Monitor.

5.4 Concomitant Medications

5.4.1 Premedications

No specific premedications or supporting medications are required in conjunction with acalabrutinib administration.

5.4.2 Prohibited or Restricted Concomitant Therapy

a) Systemic corticosteroids exceeding 7.5 mg/day for treatment of COVID-19 disease or underlying conditions is prohibited. Investigators should discuss and seek agreement with the Medical Monitor with use of corticosteroids > 7.5 mg/day during the study. It is permissible to administer systemic corticosteroids for treatment or prevention of hypersensitivity reactions.

b) Immunomodulatory drugs, eg, anti-cytokines, Btk inhibitors, Janus kinase inhibitors, PI3K inhibitors within 30 days of first dose of acalabrutinib are prohibited. Investigators should discuss and seek agreement with the Medical Monitor for individual agents

c) Warfarin (or equivalent vitamin K antagonists) within 7 days of first dose of acalabrutinib are prohibited.

d) Strong cytochrome P450 (CYP) 3A inhibitors or inducers: Drug-drug interactions may occur with some of the drugs being used as SoC (eg, drugs that are strong inducers or strong inhibitors of CYP3A). The concomitant use of strong inhibitors of CYP3A (see Appendix 2) should be avoided. If a patient requires a strong CYP3A inhibitor while on treatment with acalabrutinib, acalabrutinib treatment should be discontinued. Conversely, concomitant administration of a strong inducer of CYP3A has the potential to decrease exposure of acalabrutinib and could reduce efficacy. Therefore, the concomitant use of strong CYP3A inducers should be avoided. If a patient requires a strong CYP3A inducer while on treatment with acalabrutinib, acalabrutinib treatment should be discontinued. For additional information on drugs with potential drug-drug interactions, refer to Section 5.4.3.

e) Proton-pump inhibitors (PPIs): The effect of agents that reduce gastric acidity (antacids or PPIs) on acalabrutinib absorption was evaluated in a healthy volunteer study (ACE-HV-004). Results from that study indicate that patients should avoid the use of calcium carbonate-containing drugs or supplements for a period of at least 2 hours before and at least 2 hours after taking acalabrutinib. Use of omeprazole, esomeprazole, lansoprazole or any other PPIs while taking acalabrutinib is not recommended due to a potential decrease in study drug exposure. Although the effect of H2-receptor antagonists (such as famotidine or ranitidine) on acalabrutinib absorption has not been evaluated, if treatment with an H2 receptor antagonist is required, the H2-receptor antagonist should be taken approximately 2 hours after an acalabrutinib dose. Refer to Appendix 2 for a list of PPIs.

5.4.3 Acalabrutinib Drug-drug Interaction Guidance in the Presence of Life-threatening COVID-19 Infection

Drug-drug interaction recommendations provided for acalabrutinib in this protocol are made with respect to the presence of life-threatening COVID-19 infection and ability to achieve PD Btk receptor occupancy steady-state in target B-cell and monocytic populations. Therefore, the Sponsor recommends that all eligible patients with COVID-19 begin dosing with acalabrutinib 100 mg bid. The duration of acalabrutinib therapy will be limited to a maximum of 10 days. Table 1 provides strong or moderate CYP3A inhibitor(s), strong CYP3A inducer(s), and acid reducing agent guidance for patients with COVID-19. Refer to Appendix 2 for a list of common CYP3A inhibitors/inducers and gastric acid reducing medicines.

Table 1 Guidance on Acalabrutinib Use with CYP3A Inhibitors, CYP3A Inducers and agents that reduce Gastric Acid production and pH

	**Co-administered Medicines**	**Guidance**
CYP3A Inhibitor	Strong CYP3A inhibitor	Patients required to take strong Cytochrome P450 (CYP)3A inhibitors at baseline will be excluded from this study.<br>Consider alternative therapies to strong CYP3A inhibitors. If strong CYP3A inhibitors cannot be avoided, monitor patients closely for adverse reactions. For patients who experience an intolerable adverse event (ie, Grade 3 to 4) attributed to acalabrutinib therapy, reduce the dose to 100 mg once daily.
	Moderate CYP3A inhibitor	Monitor patients closely for adverse reactions if taking moderate CYP3A inhibitors. For patients who experience an intolerable adverse event (ie, Grade 3 to 4) attributed to acalabrutinib therapy, reduce the dose to 100 mg once daily.
CYP3A Inducer	Strong CYP3A inducer	Consider alternative therapies to strong CYP3A inducers. If these inducers cannot be avoided, increase acalabrutinib dose to 200 mg twice daily.
Gastric Acid Reducing Medicines	Proton-pump inhibitors (PPI)	Avoid concomitant use. If PPI concomitant use cannot be avoided, dose modification of acalabrutinib is not necessary; however, administration with an acidic beverage (eg, Coca-Cola Original Taste™) may improve acalabrutinib absorption.
	H_2-receptor antagonists	Take acalabrutinib 2 hours before taking a H2-receptor antagonist. If concomitant H_2-receptor antagonists are administered continuously, use above PPI recommendation.
	Antacids	Separate dosing by at least 2 hours.

The clinical pharmacology of acalabrutinib has been studied in patients with hematologic malignancies and healthy volunteers to provide CYP3A and acid-reducing agent recommendations for patients with chronic lymphocytic leukaemia and mantle cell lymphoma and have been modified for this limited duration study. In acalabrutinib clinical studies, approximately 60 patients received concomitant administration of a strong CYP3A inhibitor with acalabrutinib. Table 2 provides a summary of the duration of concomitant combination of strong CYP3A inhibitors with acalabrutinib in patients with haematologic malignancies.

Table 2 Preliminary Experience Obtained During Clinical Studies of Acalabrutinib Co-Administration with Strong CYP3A Inhibitors

Strong CYP3A Inhibitor	Number of Patients	Range (Minimum – Maximum; days)	Acalabrutinib Dose
CLARITHROMYCIN	42	1 – 34	100 mg bid
CLARITHROMYCIN	1	150	100 mg qd
CLARITHROMYCIN	2	2 – 17	200 mg bid
CLARITHROMYCIN	2	4 – 11	200 mg qd
ITRACONAZOLE	2	1 – 13	100 mg bid
VORICONAZOLE	9	2 – 78	100 mg bid
VORICONAZOLE	1	7	175 mg qd
VORICONAZOLE	1	2	200 mg bid
VORICONAZOLE	1	86	200 mg qd

bid=twice daily; CYP= Cytochrome P450; qd=once daily.

5.4.3.1 Active Substances That May Increase Acalabrutinib Plasma Concentrations

CYP3A Inhibitors (Appendix 2)

Co-administration with a strong CYP3A inhibitor (200 mg itraconazole qd for 5 days) increased acalabrutinib maximum observed concentration (C_{max}) and area under the concentration-time curve (AUC) by 3.7-fold and 5.1-fold in healthy subjects (N=17), respectively.

Alternative therapies that do not strongly inhibit CYP3A activity should be considered. In patients requiring strong CYP3A inhibitors (eg, ketoconazole, conivaptan, clarithromycin, indinavir, itraconazole, ritonavir, telaprevir, posaconazole, voriconazole), acalabrutinib treatment should be discontinued.

5.4.3.2 Active Substances That May Decrease Acalabrutinib Plasma Concentrations

CYP3A Inducers (Appendix 2)

Co-administration of a strong CYP3A inducer (600 mg rifampin qd for 9 days) decreased acalabrutinib C_{max} and AUC by 68% and 77% in healthy subjects (N=24), respectively.

Alternative therapies to strong inducers of CYP3A activity (eg, phenytoin, rifampin, carbamazepine) should be considered. St. John's wort, which may unpredictably decrease acalabrutinib plasma concentrations, should be avoided. If these inducers cannot be avoided, acalabrutinib treatment should be discontinued.

Gastric Acid Reducing Medications (Appendix 2)

Acalabrutinib solubility decreases with increasing pH. Co-administration of acalabrutinib with an antacid (1 g calcium carbonate) decreased acalabrutinib AUC by 53% in healthy subjects. Co-administration with a PPI (40 mg omeprazole for 5 days), decreased acalabrutinib AUC by 43%.

If treatment with an acid reducing agent is required, use of an antacid (eg, calcium carbonate), or an H2-receptor antagonist (eg, ranitidine or famotidine) should be considered. For use with antacids, dosing should be separated by at least 2 hours. For H2-receptor antagonists, acalabrutinib should be taken 2 hours before taking the H2-receptor antagonist.

Due to the long-lasting effect of PPIs, separation of doses with PPIs may not eliminate the interaction with acalabrutinib. In patients requiring treatment with PPIs (eg, omeprazole, esomeprazole, lansoprazole, dexlansoprazole, rabeprazole, or pantoprazole), acalabrutinib treatment should be discontinued.

Dose modification of acalabrutinib is not necessary when co-administered with gastric acid reducing medications.

5.4.4 Use in Patients with Renal Impairment

The effect of dialysis on acalabrutinib plasma concentrations has not been studied. Acalabrutinib is rapidly absorbed, metabolized, and distributed. The plasma protein binding is 97.5% and is noncovalent (potentially dialysable). However, acalabrutinib covalently binds to the target, Btk, and will not be dialysable. As such, it is unlikely that a clinically meaningful lowering of total Btk occupancy in target cell populations will be impacted. If patients with COVID-19 enrolled in this study require acute haemodialysis, it is recommended to dose acalabrutinib 100 mg and pause haemodialysis for 2 to 4 hours after acalabrutinib administration to allow for absorption and distribution to target cell populations.

5.4.5 Use in Patients with Hepatic Impairment

Acalabrutinib clinical safety has not been evaluated in patients with severe hepatic impairment. If acalabrutinib is administered to patients with hepatic impairment, patients should be monitored carefully for AEs and recommendations for dose modifications in Section 5.3 should be followed.

The PK acalabrutinib in patients with hepatic impairment has been studied. Briefly, the AUC of acalabrutinib increased 1.9-fold in patients with mild hepatic impairment (Child-Pugh class A), 1.5-fold in patients with moderate hepatic impairment (Child-Pugh class B) and 5.3-fold in patients with severe hepatic impairment (Child-Pugh class C) compared with patients with normal liver function. No clinically relevant PK difference in ACP-5862 was observed in patients with severe hepatic impairment (Child-Pugh Class C) compared with patients with normal liver function. No clinically relevant PK differences in acalabrutinib and ACP-5862 were

observed in patients with mild or moderate hepatic impairment (total bilirubin ≤ upper limit of normal [ULN] and aspartate aminotransferase [AST] > ULN, or total bilirubin > ULN and any AST) relative to patients with normal hepatic function (total bilirubin and AST within ULN).

5.5 Study Drug Information

Acalabrutinib is (S)-4-(8-amino-3-(1-but-2-ynoylpyrrolidin-2-yl)-imidazo[1,5-α]pyrazin-1-yl)-N-(pyridin-2-yl)-benzamide. Acalabrutinib is manufactured according to current Good Manufacturing Process regulations and will be supplied by the Sponsor. Acalabrutinib drug product is provided as hard gelatin capsules for oral administration. The acalabrutinib capsules contain 100 mg of acalabrutinib. The acalabrutinib capsules are packaged in opaque high-density polyethylene plastic bottles with labels bearing the appropriate label text as required by governing regulatory agencies. All formulation excipients are compendial and are commonly used in oral formulations.

Acalabrutinib should be stored according to the instructions on the label affixed to the package of the drug product.

If a drug shipment arrives damaged or if there are any other drug complaints, a Product Complaint Form should be completed and emailed or faxed to the Sponsor or the Sponsor’s representative. Refer to the pharmacy manual and the Investigator’s Brochure for additional information regarding the drug product to be used in this study.

6.0 REFERENCES

1. Huang C, Wang Y, Li X, et al. Clinical features of patients infected with 2019 novel coronavirus in Wuhan, China. Lancet. 2020;395:497-506.

2. Wu Z, McGoogan JM. Characteristics of and important lessons from the coronavirus disease 2019 (COVID 19) outbreak in China: summary of a report of 72 314 cases from the Chinese Center for Disease Control and Prevention. JAMA. 2020 Feb 24. doi: 10.1001/jama.2020.2648. [Epub ahead of print].

3. Zhou F, Yu T, Du R, et al. Clinical course and risk factors for mortality of adult inpatients with COVID 19 in Wuhan, China: a retrospective cohort study. Lancet. 2020;395:1054-62.

4. Channappanavar R, Perlman S. Pathogenic human coronavirus infections: causes and consequences of cytokine storm and immunopathology. Semin Immunopathol. 2017;39:529-39.

5. Li G, Fan Y, Lai Y, et al. Coronavirus infections and immune responses. J Med Virol. 2020;92:424-32.

6. Channappanavar R, Fehr AR, Vijay R, et al. Dysregulated type I interferon and inflammatory monocyte macrophage responses cause lethal pneumonia in SARS CoV infected mice. Cell Host Microbe 2016;19:181-93.

7. Huang KJ, Su IJ, Theron M, et al. An interferon gamma related cytokine storm in SARS patients. J Med Virol. 2005;75:185-94.

8. Wong CK, Lam CW, Wu AK, et al. Plasma inflammatory cytokines and chemokines in severe acute respiratory syndrome. Clin Exp Immunol. 2004;136:95-103.

9. Yoshikawa T, Hill T, Li K, et al. Severe acute respiratory syndrome (SARS) coronavirus induced lung epithelial cytokines exacerbate SARS pathogenesis by modulating intrinsic functions of monocyte derived macrophages and dendritic cells. J Virol. 2009;83:3039-48.

10. Herold S, Becker C, Ridge KM, et al. Influenza virus induced lung injury: pathogenesis and implications for treatment. Eur Respir J. 2015;45:1463-78.

11. Chen G, Wu D, Guo W, et al. Clinical and immunologic features in severe and moderate Coronavirus Disease 2019. J Clin Invest 2020; In Press.

12. Zhao J, Li K, Wohlford-Lenane C, et al. Rapid generation of a mouse model for Middle East respiratory syndrome. Proc Natl Acad Sci U S A. 2014;111:4970-5.

13. Zhao J, Zhao J, Van Rooijen N, et al. Evasion by stealth: inefficient immune activation underlies poor T cell response and severe disease in SARS-COV-infected mice. PLoS Pathog. 2009;5:31000636.

14. Liu L, Wei Q, Lin Q, et al. Anti-spike IgG causes severe acute lung injury by skewing macrophage responses during acute SARS-CoV infection. JCI Insight. 2019;4. pii: 123158.

15. Buggy JJ, Elias L. Bruton tyrosine kinase (BTK) and its role in B cell malignancy. Int Rev Immunol 2012;31:119-32.

16. Page TH, Urbaniak AM, Espirito Santo AI, et al. Bruton's tyrosine kinase regulates TLR7/8 induced TNF transcription via nuclear factor κB recruitment. Biochem Biophys Res Commun. 2018;499:260-6.

17. Florence JM, Krupa A, Booshehri LM, et al. Inhibition of Bruton's tyrosine kinase rescues mice from lethal influenza induced acute lung injury. Am J Physiol Lung Cell Mol Physiol 2018;315:L:52-L58.

18. O'Riordan CE, Purvis GSD, Collotta D, et al. Bruton's tyrosine kinase inhibition attenuates the cardiac dysfunction caused by cecal ligation and puncture in mice. Front Immunol. 2019 Sep 6;10:2129. doi: 10.3389/fimmu.2019.02129.

19. Byrd JC, Harrington B, O'Brien S, et al. Acalabrutinib (ACP 196) in relapsed chronic lymphocytic leukemia. N Engl J Med. 2016;274:323-32.

20. Covey T, Gulranjani M, Cheung J, et al. Pharmacodynamic evaluation of acalabrutinib in relapsed/refractory and treatment naive patients with chronic lymphocytic leukemia (CLL) in the phase 1/2 ACE CL 001 study. Blood 2017;130(Supplement 1):1741.

21. Sun CCL, Nierman PK, Kendall EK, et al. Clinical and biological implications of target occupancy in CLL treated with the BTK inhibitor acalabrutinib. Blood. 2020 Mar 20. pii: blood.2019003715. doi: 10.1182/blood.2019003715. [Epub ahead of print].

22. Moine P, McIntyre R, Schwartz MD, et al. NF κB regulatory mechanisms in alveolar macrophages from patients with acute respiratory distress syndrome. Shock 2000;13:85-91.

7.0 APPENDICES

Appendix 1 Abbreviations

Abbreviation	Definition
AE	Adverse event
AESI	Adverse event of special interest
ARDS	Acute respiratory distress syndrome
AST	Aspartate aminotransferase
AUC	Area under the concentration-time curve
bid	Twice daily
Btk	Bruton’s tyrosine kinase
C_{max}	Maximum observed concentration
COVID-19	Coronavirus disease 2019
CYP	Cytochrome P450
IL	Interleukin
MCP-1	Monocyte chemoattractant protein-1
NF	Nuclear factor
PBMC	Peripheral blood mononuclear cell
PD	Pharmacodynamic(s)
PI3K	Phosphatidylinositol-3-kinase
PK	Pharmacokinetic(s)
PPI	Proton-pump inhibitor
qd	Once daily
SARS-CoV-2	Severe acute respiratory syndrome coronavirus 2
SoA	Schedule of Activities
SoC	Standard of care
TARC	Thymus- and activation-regulated chemokine
TLR	Toll-like receptor
TNFα	Tumour necrosis factor alpha
ULN	Upper limit of normal

Appendix 2 Examples of Coadministered Drugs That Need Additional Consideration

The lists of drugs in these tables are not exhaustive. Any questions about drugs not on this list should be addressed to the Medical Monitor of this study.

Table 3 CYP3A Inhibitors

Strong inhibitors of Cytochrome P450 (CYP)3A	Moderate inhibitors of CYP3A
Boceprevir	aprepitant
clarithromycin [a]	cimetidine
cobicistat [a]	ciprofloxacin
conivaptan [a]	clotrimazole
danoprevir and ritonavir [b]	crizotinib
diltiazem [a]	cyclosporine
elvitegravir and ritonavir [b]	dronedarone [a]
grapefruit juice	erythromycin
Idelalisib	fluconazole
indinavir and ritonavir [b]	fluvoxamine
itraconazole [a]	imatinib
Ketoconazole	tofisopam
lopinavir and ritonavir [a,b]	verapamil [a]
Nefazodone	
nelfinavir [a]	
paritaprevir and ritonavir and (ombitasvir and/or dasabuvir) [b]	
Posaconazole	
ritonavir [a,b]	
saquinavir and ritonavir [a,b]	
telaprevir [a]	
tipranavir and ritonavir [a,b]	
Troleandomycin	
Voriconazole	

[a] Inhibitor of P-glycoprotein.

[b] Ritonavir is usually given in combination with other anti-HIV or anti-hepatitis C virus drugs in clinical practice. Caution should be used when extrapolating the observed effect of ritonavir alone to the effect of combination regimens on CYP3A activities.

Table 4 CYP3A Inducers

Strong inducers of CYP3A	Moderate inducers of CYP3A
Carbamazepine	bosentan
Enzalutamide	efavirenz
Mitotane	etravirine
Phenytoin	modafinil
Rifampin	
St. John's wort [a]	

[a] The effect of St. John's wort varies widely and is preparation-dependent.

Source: US FDA. Drug development and drug interactions: table of substrates, inhibitors and inducers. http://www.fda.gov/Drugs/DevelopmentApprovalProcess/DevelopmentResources/DrugInteractionsLabeling/ucm093664.htm#inVivo. Accessed 23 July 2019.

Table 5 Other Drugs Needing Additional Considerations

Proton pump inhibitors	H2-receptor antagonists
Dexlansoprazole	cimetidine
Esomeprazole	famotidine
Lansoprazole	nizatidine
Omeprazole	ranitidine
Rabeprazole	
Pantoprazole	

Source: US FDA. Established pharmacologic class text phrase. https://www.fda.gov/downloads/drugs/guidancecomplianceregulatoryinformation/lawsactsandrules/ucm428333.pdf. Accessed 23 July 2019.

Appendix 3 Contraceptive Guidance

Definitions:

Woman of Childbearing Potential (WOCBP)

A woman is considered fertile following menarche and until becoming postmenopausal unless permanently sterile (see below).

Women in the following categories are not considered WOCBP

1. Premenarchal
2. Premenopausal female with 1 of the following:
   a) Documented hysterectomy.
   b) Documented bilateral salpingectomy.
   c) Documented bilateral oophorectomy.
   Note: Documentation can come from the study center personnel's: review of the subject's medical records, medical examination, or medical history interview.
3. Postmenopausal female:
   a) A postmenopausal state is defined as no menses for 12 months without an alternative medical cause. A high follicle stimulating hormone (FSH) level in the postmenopausal range may be used to confirm a postmenopausal state in women not using hormonal contraception or hormonal replacement therapy (HRT). However, in the absence of 12 months of amenorrhea, a single FSH measurement is insufficient.
   b) Females on HRT and whose menopausal status is in doubt will be required to use 1 of the non-estrogen hormonal highly effective contraception methods if they wish to continue their HRT during the study. Otherwise, they must discontinue HRT to allow confirmation of postmenopausal status before study enrollment.

Contraception Guidance

Male subjects

- Male subjects with female partners of childbearing potential are eligible to participate if they agree to ONE of the following:
  - Are abstinent from penile-vaginal intercourse as their usual and preferred lifestyle (abstinent on a long-term and persistent basis) and agree to remain abstinent.
  - Agree to use a male condom plus partner use of a contraceptive method with a failure rate of <1% per year as described in the table below when having penile-vaginal intercourse with a woman of childbearing potential who is not currently pregnant.
- In addition, male subjects must refrain from donating sperm for the duration of the study and for 12 weeks after the last dose of study treatment.

- Male subjects with a pregnant or breastfeeding partner must agree to remain abstinent from penile-vaginal intercourse or use a male condom during each episode of penile penetration for the duration of the study and for 12 weeks after the last dose of study treatment.

Female subjects

Female subjects of childbearing potential are eligible to participate if they agree to use a highly effective method of contraception consistently and correctly as described in the table below.

Highly Effective Contraceptive Methods

**Highly Effective Contraceptive Methods That Are User Dependent** [a]<br>*Failure rate of <1% per year when used consistently and correctly.*
Combined (oestrogen and progestogen containing) hormonal contraception associated with inhibition of ovulation[b]<br>• Oral.<br>• Intravaginal.<br>• Transdermal.
Progestogen only hormonal contraception associated with inhibition of ovulation<br>• Oral.<br>• Injectable.
**Highly Effective Methods That Are User Independent** [a]
Implantable progestogen only hormonal contraception associated with inhibition of ovulation[b]<br>• Intrauterine device (IUD).<br>• Intrauterine hormone-releasing system (IUS).<br>Bilateral tubal occlusion.
**Vasectomized partner**<br>*A vasectomized partner is a highly effective birth control method provided that the partner is the sole male sexual partner of the WOCBP and the absence of sperm has been confirmed. If not, an additional highly effective method of contraception should be used.*
**Sexual abstinence**<br>*Sexual abstinence is considered a highly effective method only if defined as refraining from heterosexual intercourse during the entire period of risk associated with the study treatment. The reliability of sexual abstinence needs to be evaluated in relation to the duration of the study and the preferred and usual lifestyle of the subject.*
NOTES:<br>[a] Typical use failure rates may differ from those when used consistently and correctly. Use should be consistent with local regulations regarding the use of contraceptive methods for subjects participating in clinical studies.<br>[b] Hormonal contraception may be susceptible to interaction with the study treatment, which may reduce the efficacy of the contraceptive method. In this case, 2 highly effective methods of contraception should be utilized during the treatment period and for at least 12 weeks after the last dose of study treatment.

Appendix 4 Signature of Investigator

PROTOCOL TITLE: A Multicentre, Seamless, Phase 2/3 Adaptive Randomisation Platform Study to Assess the Efficacy and Safety of Multiple Candidate Agents for the Treatment of COVID-19 in Hospitalised Patients

SUB-PROTOCOL NO: ACCORD-2-004

SUB-PROTOCOL FOR CANDIDATE AGENT acalabrutinib

VERSION: Protocol Amendment 01

This sub-protocol is a confidential communication of the Sponsor. I confirm that I have read this sub-protocol, I understand it, and I will work according to this sub-protocol, in conjunction with the Master Protocol for the overall platform study. I will also work consistently with the ethical principles that have their origin in the Declaration of Helsinki and that are consistent with Good Clinical Practices and the applicable laws and regulations. Acceptance of this document constitutes my agreement that no unpublished information contained herein will be published or disclosed without prior written approval from the Sponsor.

Instructions to the Investigator: Please SIGN and DATE this signature page. PRINT your name, title, and the name of the study centre in which the study will be conducted. Return the signed copy to the CRO/Sponsor.

I have read this sub-protocol in its entirety and agree to conduct this part of the study accordingly:

Signature of Investigator: ___________________________ Date: ________

Printed Name: ____________________________

Investigator Title: ____________________________

Name/Address of Centre: ____________________________

____

____
