# Supplementary material for: ACCORD: A Multicentre, Seamless, Phase 2 Adaptive Randomisation Platform Study to Assess the Efficacy and Safety of Multiple Candidate Agents for the Treatment of COVID-19 in Hospitalised Patients: A structured summary of a study protocol for a randomised controlled trial
Source: Trials. 2020 Jul 31;21:691. doi: 10.1186/s13063-020-04584-9 (PMC7393340; doi:10.1186/s13063-020-04584-9)
Supplement: Supplementary file 1 — Additional file 1. [file 13063_2020_4584_MOESM1_ESM.zip › ACCORD-2-005_Heparin_SubprotocolR0.pdf]

TITLE PAGE

Master Protocol Title: ACCORD-2: A Multicentre, Seamless, Phase 2 Adaptive Randomisation Platform Study to Assess the Efficacy and Safety of Multiple Candidate Agents for the Treatment of COVID-19 in Hospitalised Patients

Master Protocol Number: ACCORD-2-005

Sub-protocol for Candidate Agent: Inhaled Unfractionated Heparin (UFH)

Study Phase: 2

Sponsor Name: University Hospital Southampton NHS Foundation Trust

Legal Registered Address: **Southampton General Hospital**
Level E, Laboratory & Pathology Block, SCBR – MP138
Tremona Road
Southampton SO16 6YD, UK

Regulatory Agency Identifying Number(s): EudraCT 2020-001736-95

IRAS Number: 282769

RHM Number: MEDI711

Date of Sub-protocol: 07 May 2020

Version: Final

Chief Investigator Signatory:

I have read this sub-protocol in its entirety and agree to conduct the study accordingly:

S.D. Singh 8 MAY 2020

Professor Dave Singh MD, FERS, FBPhS
Professor of Clinical Pharmacology and Respiratory Medicine & Honorary Respiratory Consultant

Date

TABLE OF CONTENTS

TABLE OF TABLES....4

SUB-PROTOCOL SUMMARY....5
1.1 Overview of Sub-protocol....5
1.2 Schedule of Activities....6

2.0 BACKGROUND/RATIONALE IN SUPPORT OF INHALED HEPARIN IN COVID-19....10
2.1 Dose Justification for Inhaled Heparin....11

3.0 STUDY POPULATION....13
3.1 Eligibility Criteria....13

4.0 STUDY ASSESSMENTS AND PROCEDURES....14
4.1 Safety Assessments....14
4.1.1 Adverse Events....14
4.2 Pharmacodynamic Assessments....14

5.0 STUDY TREATMENT....15
5.1 Study Drug Administration....15
5.2 Dose Modifications and Toxicity Management....16
5.2.1 Discontinuation of Study Drug....16
5.2.2 Rescue Medicine....16
5.3 Concomitant Medications....16
5.4 Study Drug Information....16

6.0 REFERENCES....17

7.0 APPENDICES....19
Appendix 1 Abbreviations....20
Appendix 2 Clinical Laboratory Tests....21
Appendix 3 Signature of Investigator....23

TABLE OF TABLES

Table 1 Safety Laboratory Assessments 22

SUB-PROTOCOL SUMMARY

1.1 Overview of Sub-protocol

In addition to the Master Protocol, this sub-protocol contains information specific to the candidate agent inhaled unfractionated heparin (UFH), including:

- an inhaled UFH specific Schedule of Activities (SOA) (Section 1.2);
- the background of the candidate agent and dose justification are explained (Section 2.0);
- potential risks and adverse events of special interest (AESIs) for inhaled UFH are detailed (Section 4.1);
- additional pharmacodynamic (PD) sample (Section 4.2);
- the formulation, dose, and route of administration for inhaled UFH are explained (Section 5.0);
- specific safety laboratory assessments for inhaled UFH (Appendix 2, Table 1).

This sub-protocol describes the approach for study conduct of the inhaled UFH sub-protocol within the ACCORD-2 platform study.

1.2 Schedule of Activities

	Screening	Baseline					
**Day (± Window)**	**Day -1 or Day 1**	**Day 1**	**Daily Until Hospital Discharge**	**Day 15[a] (±2 days)**	**Day 29[a] (±3 days)**	**Day 60[a] (±4 days) (Follow-up)**	**Day 90 (±6 days) (End of Study)**
ELIGIBILITY							
Informed consent	X						
Demographics	X						
Relevant medical history[b]	X						
Review SARS-CoV-2 diagnostic criteria	X						
Inclusion and exclusion criteria	X						
12-lead Electrocardiogram	X						
STUDY INTERVENTION							
Randomisation		X					
Administration of inhaled heparin		Every 6 hours, daily, up to 21 days					
Treatment with SoC		X	X				
STUDY PROCEDURES							
Clinical frailty score	X						
Diagnostic imaging (X-ray and/or computed tomography)	X						
Physical examination (including presenting symptoms, height, weight)	X						

	Screening	Baseline					
**Day (± Window)**	**Day -1 or Day 1**	**Day 1**	**Daily Until Hospital Discharge**	**Day 15[a] (±2 days)**	**Day 29[a] (±3 days)**	**Day 60[a] (±4 days) (Follow-up)**	**Day 90 (±6 days) (End of Study)**
Targeted physical examination (focused on lung auscultation)			X				
Vital signs, including temperature, pulse rate, blood pressure, respiratory rate, SpO_2		X[c]	X	X	X		
Clinical assessments[d]		X[c]	X	X	X		
Targeted medication review (including use of vasopressors)		X[c]	X	X	X		
Adverse event evaluation		X	X	X	X	X	X
Disease-related co-infection evaluation (including microbiologic/infectious agent assessment/results; bacteria, viral, fungi)		X	X				
Survival status		X	X	X	X	X	X
Blood gases and FiO_2 at worst PO_2[e]	X	X	X	X			
SAFETY LABORATORY							
Haematology, chemistry, liver function tests, coagulation[f]	X[g]	X[c,h]	Days 3, 5, 8, 11 (all ±1 day) while hospitalised				
Pregnancy test for females of childbearing potential	X[g]						

	**Screening**	**Baseline**					
**Day (± Window)**	**Day -1 or Day 1**	**Day 1**	**Daily Until Hospital Discharge**	**Day 15[a] (±2 days)**	**Day 29[a] (±3 days)**	**Day 60[a] (±4 days) (Follow-up)**	**Day 90 (±6 days) (End of Study)**
RESEARCH LABORATORY							
Blood (SST) for exploratory inflammatory cytokine analysis		X	Day 8	X	X		
Blood (sodium heparin tube) for PBMC phenotyping[i]		X	Day 8	X	X		
Blood (EDTA) for SARS-CoV-2 PCR (qualitative and quantitative)		X	Days 3, 5, 8, 11 (all ±1 day) while hospitalised	X	X		
Oropharyngeal/nasal swab for SARS-CoV-2 PCR (qualitative and quantitative)		X	Days 3, 5, 8, 11 (all ±1 day) while hospitalised	X	X		
Saliva SARS-CoV-2 PCR (qualitative and quantitative)		X	Days 3, 5, 8, 11 (all ±1 day) while hospitalised	X	X		
Blood (SST) for SARS-CoV-2 serology research (host response)		X	Day 8	X	X	X	
Blood (PAXGENE) for transcriptome analysis (host genome)[j]		X	Day 8	X			
Blood (EDTA) host genome (host DNA)[j]		X					
Mid-turbinate nasal swab viral genome[j]		X					
Blood (1mL) sodium citrate sample for heparin Anti-Xa		X	Days 3, 5, 8, 11 (all ±1 day) while hospitalised	X	X		

EDTA=ethylenediaminetetraacetic acid; FiO_2=fractional inspired oxygen; PBMC=peripheral blood mononuclear cell; PCR=polymerase chain reaction; PO_2= partial pressure of oxygen; RT PCR=reverse transcription polymerase chain reaction; SARS-CoV-2= severe acute respiratory syndrome coronavirus 2; SoC=standard of care; SpO_2=oxygen saturation; SST=serum separator tube; UFH=unfractionated heparin.

[a] These visits will be performed even if a patient has already been discharged. If discharged prior to scheduled visit, in-person visits are preferred, but recognising that quarantine and other factors may limit the patient's ability to return to the clinic, these visits may be conducted by phone or with a home visit by study staff. For visits conducted by telephone, it will not be possible to perform some scheduled assessments (eg, vital signs). The Day 29 assessments will also be performed, where possible, for patients who discontinue the study prematurely.

[b] Medical history includes estimated date and time of first symptoms and number of co-morbidities (eg, respiratory, cardiovascular, metabolic, malignancy, endocrine, gastrointestinal, immunologic, renal).

[c] Baseline assessments should be performed prior to study drug administration.

[d] Includes ordinal score, National Early Warning Score 2 (NEWS2), oxygen requirement, noninvasive or invasive ventilator requirement, including start and stop of low- or high-flow oxygen supply or of any form of ventilation etc.

[e] If done as part of standard of care (SoC), blood gases results to be fully recorded with date and time.

[f] For parameters, see Table 1 in Appendix 2.

[g] Laboratory tests performed in the 48 hours prior to enrolment will be accepted for determination of eligibility.

[h] Any laboratory tests performed as part of routine clinical care within the specified visit window can be used for safety laboratory testing.

[i] Samples collected for immediate laboratory processing and frozen storage.

[j] Samples collected dependent on capacity of study centre, need for reduced study burden on staff, and potentially limited access to patients.

2.0 BACKGROUND/RATIONALE IN SUPPORT OF INHALED HEPARIN IN COVID-19

Heparin is widely used as an anticoagulant; however, there is now a wide range of evidence that heparin can exhibit broad anti-inflammatory activity both experimentally and clinically, often independent of its anticoagulant activity. This includes an ability to neutralise the activity of a wide range of proinflammatory mediators.[1] In addition, there are now a number of clinical studies (outlined below) that have evaluated the beneficial effects of inhaled UFH in clinical intensive care unit (ICU) settings in burn patients and in patients with severe chronic obstructive pulmonary disease (COPD) [2,3,4,5] and emphasises the importance of using systemic low molecular weight heparin as an anticoagulant in COVID-19 infected patients, which should be given early in treatment.[6]

There is preliminary clinical evidence from China that systemic heparin has shown some benefit at reducing the impact of COVID-19 in critically ill patients.[4,5,6] In addition, several other studies in patients with acute exacerbations of chronic obstructive airways disease have shown a reduction in ventilator free days following inhaled UFH (25,000 IU).[2,8,9] Additionally, clinical benefit has been observed in patients with COPD following inhaled UFH at doses up to 75,000IU twice daily (bid).[10,3] Notably none of these studies have reported any adverse effects from bleeding following inhaled UFH, suggesting this may be a relatively safe way of treating COVID-19 patients.

The potential anti-viral effects of inhaled UFH, were previously reviewed.[1] Critically, UFH inhibits the binding of the severe acute respiratory syndrome coronavirus 2 (SARS-CoV-2) spike (S) protein to the specific angiotensin converting enzyme receptor-2 (ACE-2) receptor.[11] ACE-2 facilitates viral entry into target cells[12] and is expressed in pulmonary alveolar and bronchial epithelial cells.[13]

Additionally, glycosaminoglycans (GAGs) are an attractive target for viruses because of their wide spread distribution on the surface of most human cells, where the initial interactions with viruses occur.[14] Multiple studies have provided evidence that UFH competes with heparan sulphate proteoglycans (HSPG) for binding to surface proteins on viruses, including types of coronavirus, and limits their adhesion to cellular targets and thus their infectivity. [14]

In patients with SARS-CoV-2 airway infection, it was found that sputum samples retain SARS-CoV-2 for 39 days after throat swabs are negative.[15] Therefore, the mucolytic effects of inhaled UFH are also likely to improve clearance of virus from the airways.

The purpose of this study is to investigate the therapeutic effects and safety of inhaled UFH in patients with COVID-19 as there are currently no vaccines, no effective medicines, and only symptomatic treatments available. The benefit/risk to the patient is acceptable as data show that no acute or chronic toxicological effects have been associated with the inhaled route for any dose

of heparin investigated in humans. Further details are provided in the current version of the Investigator's Brochure. Overall, these data support the conclusion that the multiple pharmacological effects of inhaled UFH may have therapeutic benefit in patients with COVID-19.

2.1 Dose Justification for Inhaled Heparin

Heparin sodium 25,000 IU/6hours (equivalent to 25 mg delivered to the lungs/6 hours, assuming a specific activity of 200 IU/mg and that nebulised administration delivers 20% of the total dose to the lung [data on file]) for up to 21 days, or until the patient has no respiratory symptoms.

Since the discovery of heparin in 1916, there have been many toxicological studies of inhaled UFH in rodents and other small animals indicating the safety of this approach. Lung tissue from dogs that received intrapulmonary heparin (10 to15 mg/kg by intratracheal instillation) for a year showed no signs of haemorrhage, anaemia, or ulcerative lesions.[16] When heparin was instilled intratracheally in rabbits using a microspray device every 48 hours in escalating doses of 0.2, 2, 20, 100, and 200 mg/kg, signs of bleeding were seen only at the top 2 doses.[17]

No acute or chronic toxicological effects have been associated with the inhaled route for any dose of heparin in man. In healthy human subjects, 300 to 1500 mg heparin was administered as an aerosol with no sign of toxicity.[16] No incidence of pulmonary haemorrhage associated with inhaled UFH was reported in any study in a total of 536 patients with smoke inhalation injury, acute lung injury, asthma, and allergy.[18] Not even pulmonary lavage with intratracheal instillation of 200 to250 mg/kg of heparin to treat alveolar proteinosis induced bleeding into the lungs or was associated with lung damage a year later.[16] Inhaled UFH does not readily cross the bronchial mucosa. In dogs, mice, rats, and man, doses of intrapulmonary heparin > 8mg/kg are required for detectable intravenous (IV) anticoagulation[16,17] Furthermore, inhaled UFH did not induce allergic reactions in any study, not even when followed up over long periods of up to 485 days. In a study of the effect of inhaled UFH in patients with idiopathic pulmonary fibrosis, 750 mg as the nebuliser fill dose was deemed to be the threshold dose above which effects on systemic coagulation could first be detected.[19] In a compassionate phase of the study, patients continued to use inhaled UFH for up to 100 weeks, without side effects.

Previous studies[1] have indicated inhalation of heparin treats local inflammation, mucus hypersecretion, and lung injury, without systemic anticoagulation (20 studies, 536 patients)[18] and is safe and effective in patients with smoke inhalation injury, acute lung injury, asthma and allergy, cystic fibrosis,[20] and COPD. In another clinical study in patients with moderate to severe COPD, approximately, 60 mg UFH delivered to the lung significantly improved lung function, exercise capacity, and dyspnoea with no evidence for toxicity or adverse side effects at this dose.[3]

For COVID-19, inhaled UFH may offer benefit for patients to reduce the complications arising from a cytokine storm and to prevent the magnitude of the effects of a cytokine storm in patients with moderate to severe COVID-19 driven disease. The recent data suggest that inhaled UFH may also bind the Spike 1 protein that the COVID-19 virus uses to enter cells and may provide an additional anti-viral effect in patients with COVID-19. Those with pneumonic consolidation and consequent increased sputum production will be treated by the mucolytic effect of inhaled UFH.[3,21]

3.0 STUDY POPULATION

3.1 Eligibility Criteria

No additional inclusion and exclusion criteria are required for this sub-protocol; only those presented in the Master Protocol are required.

4.0 STUDY ASSESSMENTS AND PROCEDURES

In addition to the study assessments and procedures described in Section 8.0 of the Master Protocol, assessments specific to the sub-protocol will be performed as described in the following sections. The SoA for this sub-protocol is presented in Section 1.2.

4.1 Safety Assessments

4.1.1 Adverse Events

4.1.1.1 Adverse Events from Approved Indications

Inhaled UFH is not approved for any indication. Intravenous heparin is approved and is generally well-tolerated. Adverse reactions reported per the approved labelling for IV heparin include, haemorrhage, heparin-induced thrombocytopenia (HIT) and heparin-induced thrombocytopenia and thrombosis (HITT), thrombocytopenia, heparin resistance, and hypersensitivity; however, these events have not been reported with inhaled UFH due to reduced systemic absorption.

Patients receiving inhaled UFH can expect to expectorate larger volumes of sputum, reflecting the pharmacological effect of a mucolytic, and although this is not an AE, patients may score this effect negatively on visual analogue scales reflecting body image or social marginalization.[20] Therefore, patients should be advised of this likely effect and that it is a beneficial effect (Investigator's Brochure).

Additionally, possible adverse effects associated with inhaled UFH include:

- Headache
- Epistaxis
- Systemic anticoagulation (this is not expected at the inhaled dose used)
- Haemoptysis (although for all the reasons given above this is unlikely to be associated with inhaled UFH and is as likely to be seen in the placebo group)

4.2 Pharmacodynamic Assessments

Blood sodium citrate sample for heparin Anti-Xa assay will be collected on Day 1, 3, 5, 8 ±1 day) while hospitalised, Day 15, and Day 29 (See Section 1.2).

5.0 STUDY TREATMENT

5.1 Study Drug Administration

Heparin sodium will be provided in 1-mL ampoules of 25,000 IU/mL without preservative (Monoparin 25,000 IU/mL solution for injection, Wockhardt UK).

Heparin sodium will be administered as a nebulised aerosol dose of 25,000 IU heparin every 6 hours diluted to 4 mL in 0.9% saline via an Aerogen Solo (Aerogen, Ireland) vibrating mesh aerosol drug nebulizer. It has been estimated that 20% of the heparin dose is administered to the lungs by this method using Anderson cascade impactor (data on file).

Doses will be made up immediately before use as follows: one 1mL ampoule of Heparin sodium 25,000 IU/mL without preservative will be added to the nebulizer chamber followed by 4.0 mLs of Sodium Chloride Injection BP 0.9%. The chamber will be assembled and swirled to mix the heparin thoroughly.

Nebulised Heparin Sodium 25,000 IU will be delivered using air or oxygen every 6 hours, daily for up to 21 days, or until the patient has no respiratory symptoms.

The mode of delivery (via mask, mouthpiece, or endotracheal tube) will be chosen by the clinical team depending on the patient's needs for oxygen and ventilatory support.

5.2 Dose Modifications and Toxicity Management

There is no need for dose modification in either renal or hepatic impairment because no acute or chronic toxicological effects have been associated with the inhaled route for any dose of heparin investigated in man. Studies in healthy adults have shown no detectable systemic absorption of inhaled UFH (evaluated by blood coagulation time) at the dosage proposed.

5.2.1 Discontinuation of Study Drug

Discontinuation of the study drug should be considered by the Investigator when a patient meets one of the following conditions:

- Bronchospasm which cannot be ameliorated by pre-treatment with bronchodilators.
- Sudden hypoxia; oxygen saturation should be monitored throughout nebulisation of UFH.

5.2.2 Rescue Medicine

Although allergic reaction has not been reported in any study, anaphylaxis is a theoretical possibility. Should it occur, it should be treated per standard of care.

5.3 Concomitant Medications

There are no contraindications to concomitant medications.

5.4 Study Drug Information

Clinical heparin marketed in the UK is an animal polysaccharide extracted from porcine intestinal mucosa. Its clinical effects derive from multiple sulfur trioxide ($SO_{3)}$ groups which make it the most negatively charged polyanionic molecule in biology. Heparin’s licensed use as an anticoagulant (ATC BO1AB0 in the BO1A group of anti-thrombotic agents) derives from its ability to bind and potentiate antithrombin, the anionic plasma coagulation inhibitor protein. Unfractionated heparin, however, is a mixture of polysaccharide chains with molecular masses ranging from 5,000 to 50,000 Da, giving it a broad repertoire of positively-charged targets. Of particular relevance, heparin has been shown to bind pro-inflammatory molecules including cytokines interleukin (IL)-4, IL-5, IL-6 and IL-8, chemokines, adhesion molecules cytotoxic peptides, and tissue-degrading enzymes. Heparin also inhibits mast cell and neutrophil activation.

Heparin Sodium 25,000 IU/mL without preservative (Monoparin 25,000 IU/mL Solution for injection, Wockhardt UK) also contains as inactive excipients: water for injections, sodium hydroxide solution 3M, and hydrochloric acid 3M.

6.0 REFERENCES

1. Mulloy B, Hogwood J, Gray E, Lever R, Page CP. Pharmacology of heparin and related drugs. Pharmacol Rev 2016;68:76-141.

2. McIntire A, Harris S, J Whitten, et al. Outcomes following the use of nebulised heparin after inhalation injury (HIHI study). J Burn Care Res 2017;38: 45-52.

3. Shute JK, Calzetta L, Cardaci V, di Toro S, Page CP, Cazzola M. Inhaled nebulised unfractionated heparin improves lung function in moderate to very severe COPD: A pilot study. Pulm Pharmacol Ther. 2018; 48:88-96.

4. Zhang Y, Cao W, Xiao M, et al. [Clinical and coagulation characteristics of 7 patients with critical COVID-2019 pneumonia and acro-ischemia]. Zhonghua Xue Ye Xue Za Zhi. 2020 Mar 28;41(0):E006. doi: 10.3760/cma.j.issn.0253-2727.2020.0006. [Epub ahead of print] Chinese.

5. Tang N, Bai H, Chen X, Gong J, Li D, Sun Z.Anticoagulant treatment is associated with decreased mortality in severe coronavirus disease 2019 patients with coagulopathy Thromb Haemost. 2020 Mar 27. doi: 10.1111/jth.14817. [Epub ahead of print]. PMID:32220112.

6. Li T, Lu H, Zhang W. Clinical observation and management of COVID-19 patients. Emerg Microbes Infect. 2020 Dec;9(1):687-690. doi: 10.1080/22221751.2020.1741327. PMID:32208840.

7. Chimeri L et al, Nebulized Heparin Attenuates Pulmonary Coagulopathy and Inflammation Through Alveolar Macrophages in a Rat Model of Acute Lung Injury. Throm Haem 2017; 117: 2125-2134.

8. Glas GJ, Serpa Neto A, Horn J, et al. Nebulized heparin for patients under mechanical ventilation: an individual patient data meta-analysis. Ann Intensive Care. 2016;6(1):33.

9. Dixon B, Schultz MJ, Smith R, Fink JB, Santamaria JD, Campbell DJ. Nebulized heparin is associated with fewer days of mechanical ventilation in critically ill patients: a randomized controlled trial. Crit Care. 2010;14(5):R180.

10. Ashoor TM, Hasseb AM, Esmat IM. Nebulized heparin and salbutamol versus Salbutamol alone in acute exacerbation of chronic obstructive pulmonary disease requiring mechanical ventilation: a double blind randomised controlled trial. Korean J Anesthesiol. 2020 Feb 28. doi: 10.4097/kja.19418. [Epub ahead of print]

11. Mycroft-West C, Su D, Elli S, Guimond S, et al. The 2019 coronavirus (SARS-CoV-2) surface protein (Spike) S1 Receptor Binding Domain undergoes conformational change upon heparin binding. BioRxiv 2020.

12. Hoffmann M, Kleine-Weber H, Schroeder S, et al. SARS-CoV-2 Cell Entry Depends on ACE2 and TMPRSS2 and Is Blocked by a Clinically Proven Protease Inhibitor. Cell. 2020 Mar 4. [Epub ahead of print].

13. Hamming I, Timens W, Bulthuis ML, Lely AT, Navis G, van Goor H. Tissue distribution of ACE2 protein, the functional receptor for SARS coronavirus. A first step in understanding SARS pathogenesis. J Pathol. 2004 Jun;203(2):631-7.

14. Cagno V, Tseligka ED, Jones ST, Tapparel C. Heparan Sulfate Proteoglycans and Viral Attachment: True Receptors or Adaptation Bias? Viruses. 2019 Jul 1;11(7).

15. Chen C, Gao G, Xu Y,et al. SARS-CoV-2-Positive Sputum and Feces After Conversion of Pharyngeal Samples in Patients With COVID-19. Ann Intern Med. 2020 Mar 30. [Epub ahead of print].

16. Jacques LB, Mahadoo J, Kavanagh LW. Intrapulmonary heparin. Lancet 1976; 308: 1157-1161.

17. Williams PD, Tyrrell DJ, Storm NE, Holme KR. Use of a precise intratracheal delivery system to compare the acute tolerance of heparin and the heparinoid GM2000 in rabbits. Toxicology Mechanisms and Methods 1997; 7: 1-7.

18. Monagle K, Ryan A, Hepponstall M, et al. Inhalational use of antithrombotics in humans: Review of the literature. Thrombosis Res 2015;136:1059-1066.

19. Markart P, Nass R, Ruppert C, et al. Safety and tolerability of inhaled heparin in idiopathic pulmonary fibrosis. J Aerosol Med Pulm Drug Deliv. 2010;23:161-72.

20. Serisier DJ, Shute JK, Hockey PM, Higgins B, Conway J, Carroll MP. Inhaled heparin in cystic fibrosis. Eur Respir J. 2006;27:354-8.

21. Shute JK, Puxeddu E, Calzetta L. Therapeutic use of heparin and derivatives beyond anticoagulation in patients with bronchial asthma or COPD. Curr Opin Pharmacol. 2018; Jun;40:39-45. doi: 10.1016/j.coph.2018.01.006. Epub 2018 Feb 20. Review.

7.0 APPENDICES

Appendix 1 Abbreviations

Abbreviation	Definition
ACE-2	Angiotensin converting enzyme receptor-2
AE	Adverse event
AESI	Adverse event of special interest
COPD	Chronic obstructive pulmonary disease
IL	Interleukin
IV	Intravenous
PD	Pharmacodynamic
SARS-CoV-2	Severe acute respiratory syndrome coronavirus 2
SOA	Schedule of Activities
UFH	Unfractionated heparin

Appendix 2 Clinical Laboratory Tests

The tests to be performed for the inhaled unfractionated heparin (UFH) arm are detailed in Table 1.

Local laboratory results are only required in the event that the central laboratory results are not available in time for either study treatment administration and/or response evaluation. If a local sample is required, it is important that the sample for central analysis is obtained at the same time. Additionally, if the local laboratory results are used to make either a study treatment decision or response evaluation, the results must be entered into the case report from.

Additional tests may be performed at any time during the study as determined necessary by the Investigator or required by local regulations.

Changes to some laboratory parameters are anticipated for any patients moving on to extracorporeal membrane oxygenation therapy.

Investigators must document their review of each laboratory safety report.

Table 1 Safety Laboratory Assessments

**Laboratory Assessments**	**Parameters**
Haematology	Platelet Count<br>Haemoglobin<br>White blood cell count with differential:<br>Neutrophils<br>Lymphocytes<br>Monocytes<br>Eosinophils<br>Basophils
Coagulation	D-dimer test (if possible)<br>Fibrinogen<br>Activated partial thromboplastin time (aPTT)<br>Prothrombin time (PT)<br>International Normalised Ratio (INR)<br>Activated partial thromboplastin time (aPTT) ratio
Clinical Chemistry	Potassium<br>Sodium<br>Calcium<br>Magnesium<br>Phosphate<br>Alkaline phosphatase<br>Bicarbonate<br>Creatinine<br>Creatine kinase (MB fraction)<br>Glucose<br>Total bilirubin<br>Aspartate aminotransferase (AST)<br>Alanine aminotransferase (ALT)<br>Gamma-glutamyl transferase (GGT)<br>C-reactive protein<br>Ferritin<br>Triglycerides<br>Lactate dehydrogenase (LDH)<br>Troponin

Appendix 3 Signature of Investigator

PROTOCOL TITLE: A Multicentre, Seamless, Phase 2 Adaptive Randomisation Platform Study to Assess the Efficacy and Safety of Multiple Candidate Agents for the Treatment of COVID-19 in Hospitalised Patients

PROTOCOL NO: ACCORD-2-005

SUB-PROTOCOL FOR CANDIDATE AGENT INHALED UNFRACTIONATED HEPARIN

VERSION: Original Protocol

This sub-protocol is a confidential communication of the Sponsor. I confirm that I have read this sub-protocol, I understand it, and I will work according to this sub-protocol, in conjunction with the Master Protocol for the overall platform study. I will also work consistently with the ethical principles that have their origin in the Declaration of Helsinki and that are consistent with Good Clinical Practices and the applicable laws and regulations. Acceptance of this document constitutes my agreement that no unpublished information contained herein will be published or disclosed without prior written approval from the Sponsor.

Instructions to the Investigator: Please SIGN and DATE this signature page. PRINT your name, title, and the name of the study centre in which the study will be conducted. Return the signed copy to the CRO/Sponsor.

I have read this sub-protocol in its entirety and agree to conduct this part of the study accordingly:

Signature of Investigator: ___________________________ Date: ________

Printed Name: ____________________________

Investigator Title: ____________________________

Name/Address of Centre: ____________________________

____

____
